# Keto-Polyethylene Material from Pd(II)-Catalyzed Copolymerization with Continuous Carbon Monoxide Feed

**DOI:** 10.1021/acscatal.5c00935

**Published:** 2025-05-02

**Authors:** Steffen Iberl, Maria Voccia, Ida Ritacco, Lukas Odenwald, Maximilian Baur, Laura Falivene, Lucia Caporaso, Stefan Mecking

**Affiliations:** † Chair of Chemical Materials Science, Department of Chemistry, University of Konstanz, 78464 Konstanz, Germany; ‡ Department of Chemistry, University of Salerno, Fisciano, Salerno 848048, Italy

**Keywords:** keto-modified polyethylene, HDPE-like, copolymerization, late transition metal, catalyst, density functional theory, material properties

## Abstract

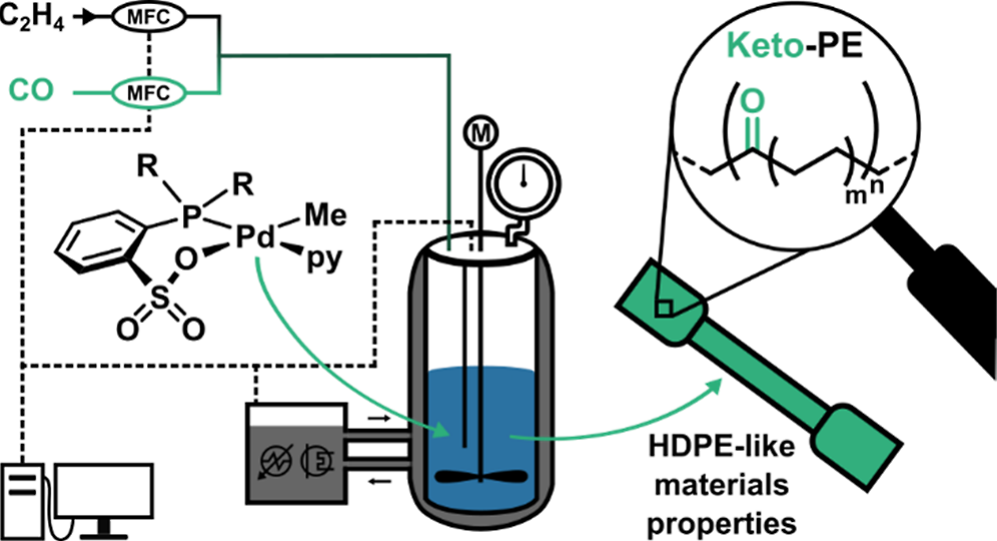

Pd(II) phosphinosulfonate catalysts were employed in the nonalternating copolymerization of ethylene and carbon monoxide to produce keto-polyethylenes with high-density polyethylene-like materials properties. The different reactivities of the two monomers were addressed with a customized reactor setup that allows the feeding of ethylene and CO at very different feed ratios and automatic repressurization to replenish consumed monomers upon reaching a pressure threshold. Four literature-known catalysts were screened and the keto group microstructure of the resulting keto-PEs aligned well with the activation free energy differences (ΔΔG^‡^) of the alternating and nonalternating pathways, calculated via density functional theory. **Pd-2** with a 2′,6′-dimethoxy-1,1′-biphenyl-substituted phosphine motif was the most active catalyst, yielding copolymers with the highest molecular weight (around 30–40 kg mol^–1^). Consequently, **Pd-2** was subjected to further optimization of the E/CO copolymerization to obtain HDPE-like materials. Tensile-testing specimens of keto-PEs with 0.5 and 1.4 mol % of keto groups were obtained via melt pressing and exhibited mechanical properties on par with the HDPE reference material.

## Introduction

**Scheme 1:**
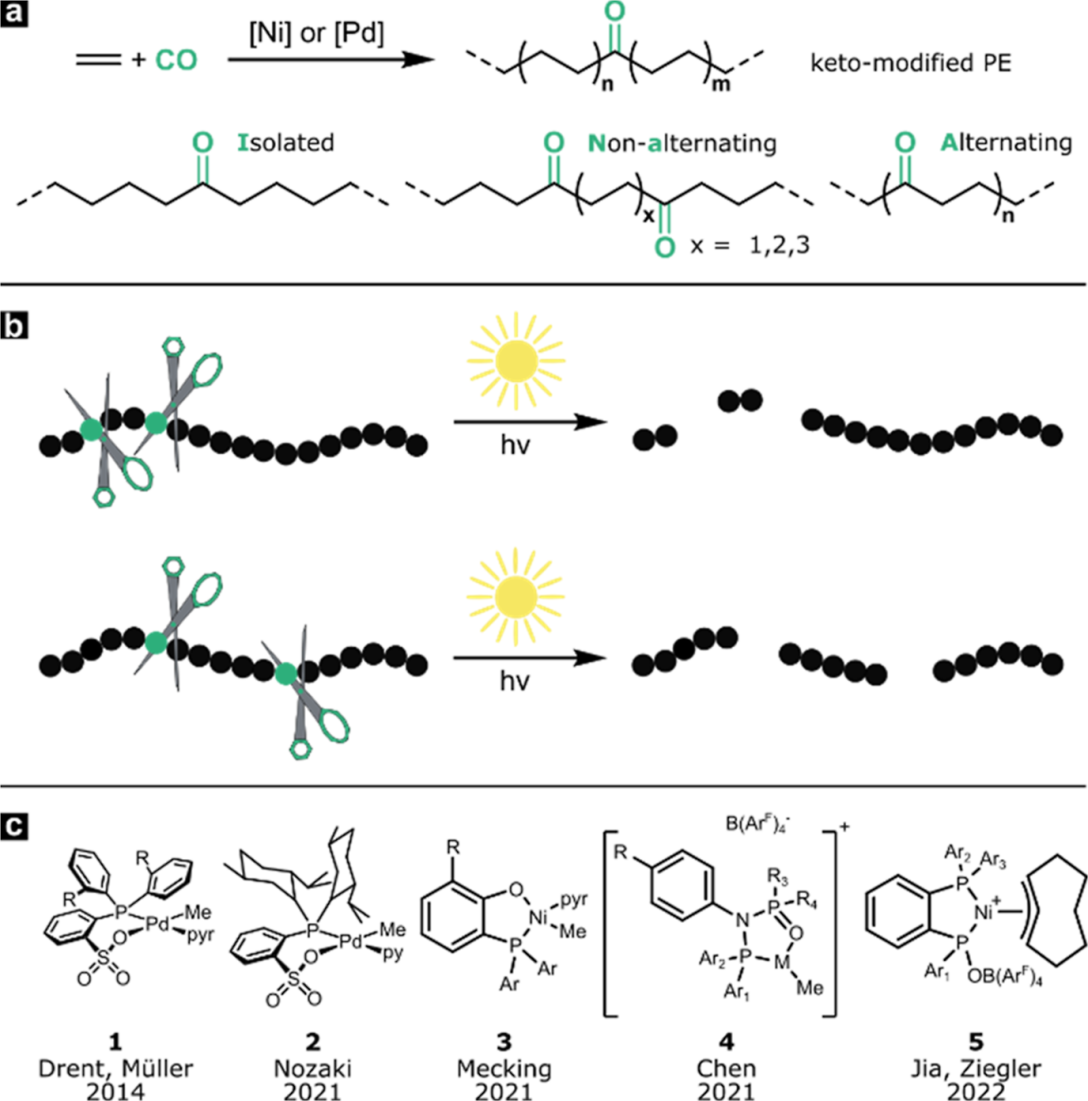
(a) Nonalternating Copolymerization of Ethylene and Carbon Monoxide toward Keto-Modified Polyethylene; (b) Influence of the Keto Group Microstructure on the Chain Length of the Products from UV-Light-Induced Chain Cleavage; (c) Pd/Ni Complexes Capable of Catalyzing the Nonalternating Ethylene/CO Copolymerization

Polyethylene is the most abundantly used synthetic polymer of our time.^1^ It is so popular because it combines excellent processability and low production costs with versatile mechanical properties.^2,3^ As a downside, mismanaged polyethylene waste accumulates in virtually any environment where it can persist for decades or centuries. Even with a future comprehensive implementation of an efficient waste collection system, a leakage of plastics to the environment must be considered. A decreased environmental persistency of polyethylene is therefore desirable. This can be achieved by the introduction of low densities of functional groups in the polymer chain to enable eventual breakdown.^4−8^ An incorporation of carbon monoxide comonomer during transition-metal-catalyzed polyethylene chain growth can generate photodegradable in-chain keto units (see Scheme 1a).^9,10^

A long-standing challenge in catalytic polymerization has been the high propensity for incorporation of CO over ethylene due to its strong binding to the active centers and low barriers of insertion. This results in the formation of high-melting alternating polyketones rather than keto-modified polyethylenes.^11−13^ A lower incorporation of carbon monoxide with mainly isolated and nonalternating keto groups instead of alternating motifs is desired to maintain the thermal and material properties of high-density polyethylene (see Scheme 1a).^10,14,15^ Additionally, the UV-light-induced chain scission of keto-PE with mostly isolated and evenly distributed keto groups leads to a more uniform chain length distribution which is supposed to be beneficial for further biodegradation of the cleavage products as the maximum chain length is shorter compared to a polymer with the same overall molecular weight and keto content but with a more alternating structure (see Scheme 1b).^14^ A significant advance was recently achieved with Ni(II)-catalyzed nonalternating copolymerization, which can yield high molecular weight copolymers (up to *M*_w_ 400,000 g mol^–1^; *M*_n_ 200,000 g mol^–1^) with desirable low keto contents (e.g., 1 mol %) (see Scheme 1c, **3**). These are processable via injection-molding and possess tensile properties akin to high-density polyethylene (HDPE).^10^ In this regard, the materials from Ni(II) catalysis are singular to date. This raises the question whether the generation of keto-PE materials is limited to this approach or whether they can be achieved more broadly by catalysis with, e.g., other metals. In our opinion, the relevant benchmark for HDPE-like materials is actual materials testing along with melt processing, as fundamental characteristics such as ductility cannot be predicted from molecular characterization methods. The most versatile catalytic system for the incorporation of polar vinyl monomers is Pd(II) phosphinosulfonate complexes (see Scheme 1c, **1**).^16−20^ These are also capable of nonalternating ethylene/CO copolymerization, as observed by Drent et al. early on.^21^ More recently, cationic diphosphazane monoxide Ni(II) and Pd(II) catalysts (see Scheme 1c, **4**) along with cationic diphosphine Ni(II) catalysts (see Scheme 1c, **5**) have also been found to copolymerize ethylene and carbon monoxide in a nonalternating fashion.^22−24^

**Scheme 2:**
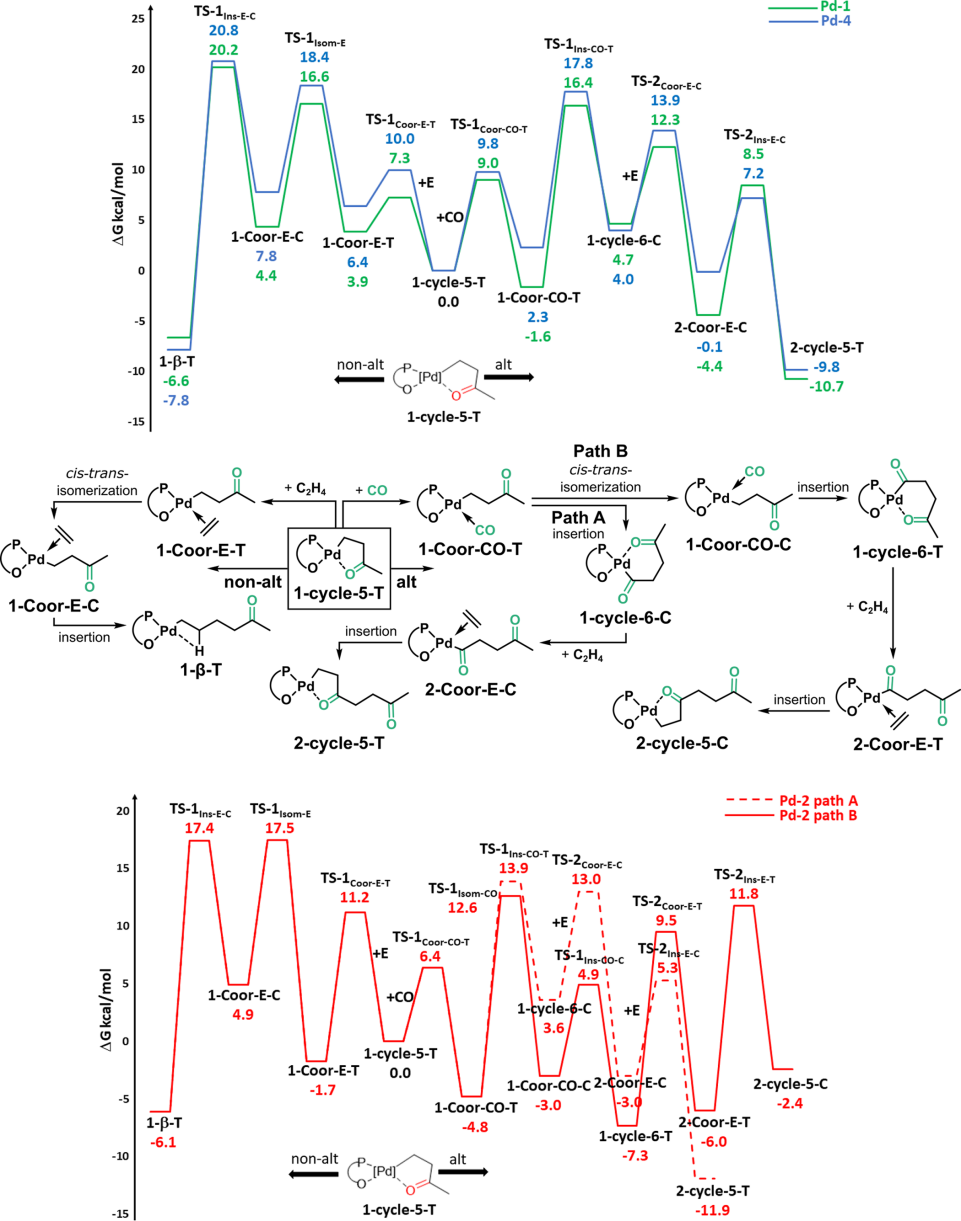
Free Energies (Δ*G*_tol_ in kcal/mol) of the Key Steps for Nonalternating and Alternating Carbon Monoxide Incorporation with Catalysts **Pd-1** (Green), **Pd-2** (Red), and **Pd-4** (Blue) The labels 1-* refer to species involved in monomer incorporation (E or CO) from **1-cycle5-T** and the 2-* to the next (second) monomer (CO + E) incorporation.

In the aforementioned studies of Pd(II) phosphinosulfonates, Drent and co-workers observed subsequent ethylene insertions in addition to the formation of alternating motifs, which resulted in a decreased melting point.^21^ Later, Müller and co-workers were able to obtain polyethylene with lower CO incorporations down to 1.5 mol %, resulting in a PE-like melting point of *T*_m_ = 125 °C. However, these materials suffer from low molecular weights and brittleness.^25^ Nozaki et al. revealed in parallel to our work that *P*-menthyl-substituted Pd(II) phosphinosulfonate catalysts (see Schemes 1c, **2**) can afford keto-polyethylenes with exclusively isolated keto units in the chain, that also possess high molecular weights (up to *M*_w_ 120,000 g mol^–1^; *M*_n_ 60,000 g mol^–1^). In this study, low concentrations of CO were provisioned elegantly from metal carbonyls present in the reaction mixture to yield keto-modified polyethylene with up to 3.9 mol % of keto groups.^14^ Alternatively, carbon dioxide (CO_2_) can be converted to carbon monoxide in a tandem reaction. This approach requires compatibilization of the conditions required for the conversion of CO_2_ to CO with those necessary for copolymerization. Liu, Miller, and co-workers utilized the electrochemical reduction of carbon dioxide at Pd electrodes in a nonaqueous medium for this purpose. A vial-in-vial approach yielded nonalternating copolymers with CO incorporations down to 3 mol % within a small window of low current densities to reduce the rate of electrochemical CO_2_ reduction and concomitantly the rate of CO generation.^26^ Tang, Zhou, and co-workers photochemically reduced carbon dioxide at higher temperatures which releases lower amounts of carbon monoxide and enabled nonalternating copolymers with CO incorporations between 0.2 and 5.0 mol %. The materials exhibited high amounts of isolated keto groups and HDPE-like thermal properties.^27^ However, all methods to supply carbon monoxide in a controllable fashion require additional chemical compounds or complex tandem approaches which come together with different compromises regarding suitable reaction conditions.^14,26,27^

The direct feeding of CO rather than its generation from other reagents during the copolymerization appears more practical and enables variation and knowledge of the actual monomer feed concentrations. A possible approach to the challenge of feeding low CO concentrations relative to the ethylene feed is premixing of the gases in a high-pressure vessel, like a high-pressure piston pump which then can serve as a reservoir.^10^ A more versatile and general approach is the direct feeding of both monomers. However, reliable feeding of both monomers at very different flow rates is challenging to achieve.

We now report that the appropriate choice of Pd(II) phosphinosulfonate catalyst, enabled by controlled monomer feeding and rationalized by theoretical analysis, can yield keto-polyethylenes with HDPE-like materials properties.

## Results and Discussion

### Benchmarking of Catalyst Performance and Copolymer Microstructures

**Figure 1:**
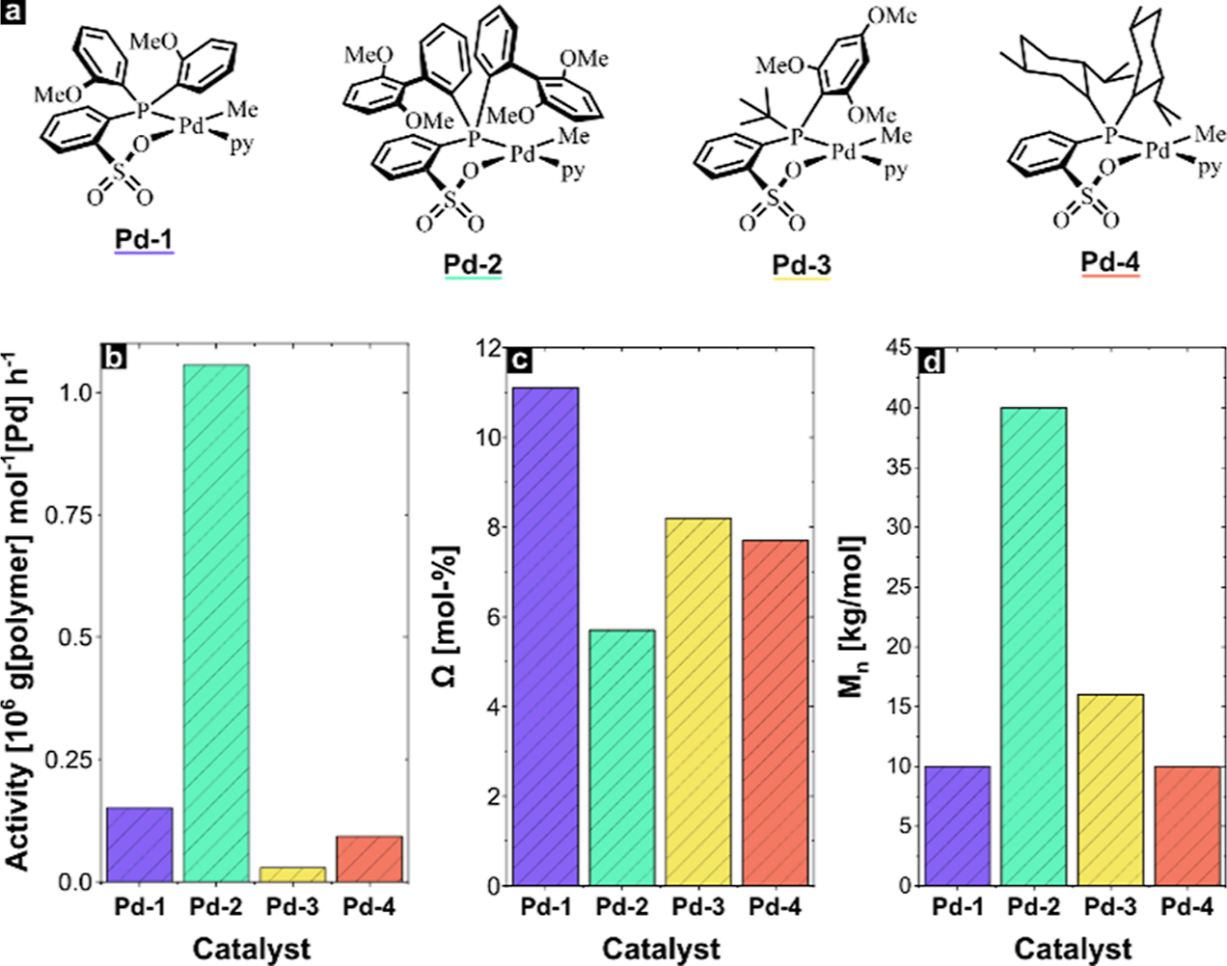
(a) Structures of the Pd(II) phosphinosulfonate catalysts (**Pd-1–Pd-4**) used in E/CO copolymerization reactions and a comparison of (b) catalyst activities, (c) keto group incorporations, and (d) copolymer molecular weights from these initial copolymerization reactions (lower section).

We selected four previously reported Pd(II) phosphinosulfonate catalysts **Pd-1**–**Pd-4** (see Figure 1), based on (1) their performance in ethylene-polar vinyl comonomer copolymerizations with the anticipation that this translates into sufficient tolerance toward CO to enable the targeted nonalternating E/CO copolymerization and (2) their ability to polymerize ethylene to high molecular weights, the latter being a prerequisite for achieving HDPE-like ductility.^17,18,21,28−30^ The catalysts were screened under identical conditions to compare their tolerance to CO and the microstructure of the resulting copolymers (**KPE1**–**KPE4**) including molecular weight, CO incorporation, and the distribution of the keto groups throughout the backbone (see Table 1). All four catalysts were active toward E/CO mixtures and produced nonalternating copolymers. **Pd-2** stands out as the most active catalyst (37.7 × 10^3^ mol [C_2_H_4_] mol^–1^ [Pd] h^–1^). All four catalysts incorporated keto groups into the apolar hydrocarbon backbone in a nonalternating manner (≤5% of incorporated keto groups were present as alternating motifs). By using a monomer feed with 0.13 mol % of CO (in terms of relative pressure, 40 bar of ethylene and 0.05 bar of CO), **Pd-1** incorporated the highest density of keto groups (11.1 mol %), while **Pd-2** incorporated the least (5.4 mol %). Both the quantity of keto group incorporation and the microstructure of the polymer backbone significantly influence the copolymer properties. The precise microstructure of the keto groups was elucidated by ^1^H NMR spectroscopy (see Figure S21). Copolymer **KPE4** exhibited the highest percentage of isolated keto groups with no indication of alternating E/CO segments. Although isolated keto groups were the most prominent motif in all four copolymers, the portion of these groups clearly decreased in the order **KPE4** > **KPE3** > **KPE1** > **KPE2**. Also, under the reaction conditions studied here, Nozaki’s catalyst, **Pd-4** is clearly superior in generating isolated keto motifs.

**Table 1:** Copolymerization Results in the Presence of Pd(II) Phosphinosulfonate Catalysts **Pd-1**–**Pd-4**

no.	catalyst	*n*_cat_ (μmol)	*t* (min)	yield (mg) (activity^a^)	Ω^b^(mol %)	I/NA/A^c^ (%)	*M*_n_ (kg mol^–1^) (*M*_w_/*M*_n_)^d^
**KPE1**	**Pd-1**	5.0	5	63 (0.151)	11.1	65/31/4	10 (1.5)
**KPE2**	**Pd-2**	0.5	5	44 (1.056)	5.7	60/35/5	40 (1.8)
**KPE3**	**Pd-3**	5.0	30	69 (0.028)	8.2	78/20/2	16 (1.2)
**KPE4**	**Pd-4**	5.0	15	93 (0.074)	7.7	90/10/0	10 (1.4)

a Copolymerization conditions: 200 mL toluene, 90 °C, 40 bar ethylene, 0.05 bar CO, KPE: keto-modified polyethylene; activity given in 10^6^ g [polymer] mol^–1^ [Pd] h^–1^.

b CO incorporation determined by ^1^H NMR spectroscopy at 383 K in C_2_D_2_Cl_4_.

c Ratio of isolated/nonalternating/alternating keto groups determined by ^13^C NMR spectroscopy at 383 K in C_2_D_2_Cl_4_.

d Determined by gel permeation chromatography (GPC) in 1,2-dichlorobenzene at 160 °C linear calibration versus polyethylene standards.

As another key characteristic, molecular weights of the formed polymers were determined by SEC, vs PE standards. **KPE-2** stands out with a molecular weight of *M*_n_ = 40 kg mol^–1^, compared to the polymers obtained with the other catalysts under the same polymerization conditions studied here (see Table 1). Based on this, and its high activity, **Pd-2** was chosen for further experimental studies (vide infra).

### Alternating vs Nonalternating CO Incorporation Illuminated by Density Functional Theory (DFT)

**Figure 2:**
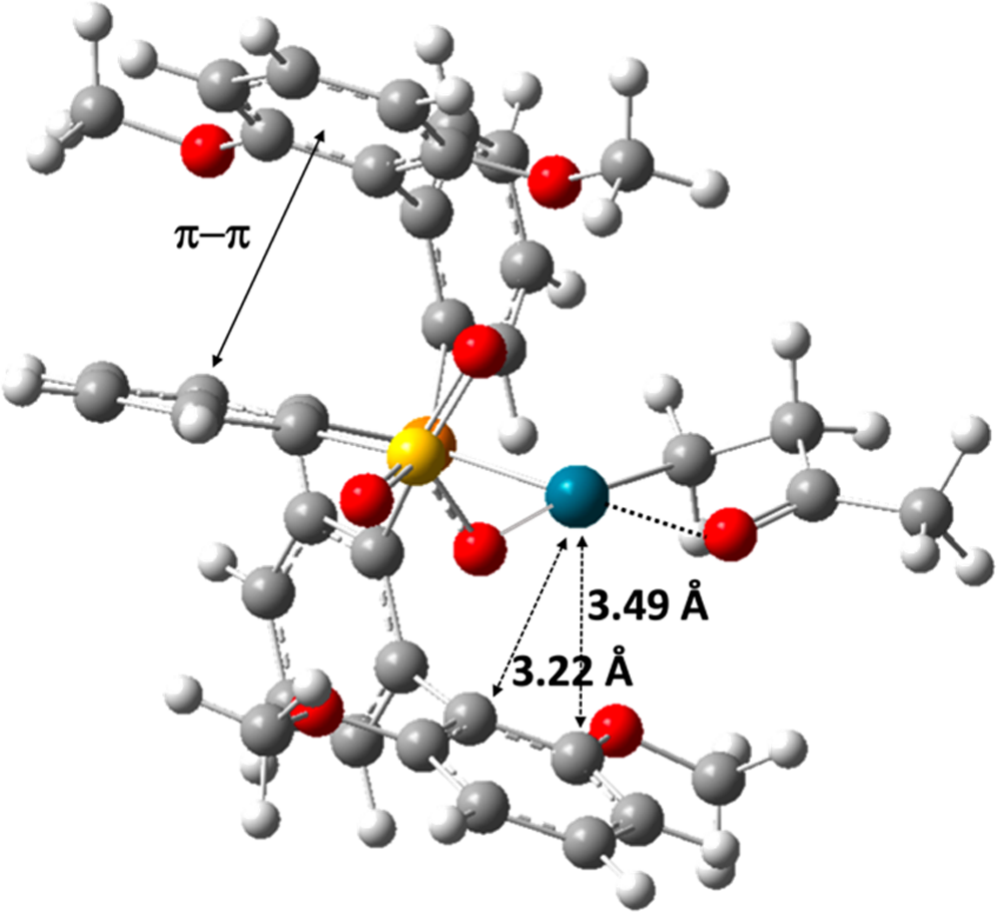
Geometry of the chelate **1-cycle5-T** intermediate for **Pd-2**.

To rationalize the differences in the keto-PE microstructures obtained with various catalysts in the above back-to-back experimental study, the mechanism of ethylene/CO copolymerization was studied using DFT calculations for selected Pd(II) phosphinosulfonate complexes, specifically **Pd-2** and **Pd-4** (see Scheme 2). These calculations aimed to elucidate the effect of the phosphine substituents—R = 2′,6′-dimethoxy-(1,1′-biphenyl) for **Pd-2** and R = menthyl for **Pd-4**—on the copolymer microstructure, with comparisons made with the previously studied **Pd-1**.^31^ In line with previous studies, the five-membered intermediate **1-cycle5-T** was used as the zero-point energy reference. This chelate features the growing alkyl chain trans to the oxygen atom of the ligand, representing the starting point for both copolymerization pathways, which leads to the formation of nonalternating keto-modified polyethylene and alternating polyketone segments.^31^ As reported for Ni(II) phosphinophenolate complexes, a favorable η^2^ interaction of the 2,6-(OMe)_2_C_6_H_3_ ring on the phosphine moiety with the metal center was observed for **Pd-2**.^31^ Additionally, **Pd-2** exhibits a stabilizing π–π interaction between the second bis-phenyl substituent on the phosphorus donor and the aromatic ring in the ligand backbone (see Figure 2).

From **1-cycle5-T**, the coordination of ethylene via **TS-1**_**Coor-E-T**_ to form **1-Coor-E-T** occurs through the opening of the metal···O interaction, with a free energy barrier of about 10 kcal/mol for both complexes **Pd-2** and **Pd-4**. The resulting **1-Coor-E-T** intermediate is disfavored by approximately 6 kcal/mol for **Pd-4**, similar to what was previously reported for **Pd-1**.^31^ In contrast, it is 1.7 kcal/mol more stable for **Pd-2**, likely due to the higher electron density at the palladium center resulting from the metal–aryl interaction as supported by charge analysis on **1-cycle-5-T**, which shows a higher charge on Pd in **Pd-2** (−0.15) compared to **Pd-1** (−0.3). This metal–aryl interaction strengthens the binding of ethylene by enhancing back-donation from the metal to the olefin. From **1-Coor-E-T**, the favored nonalternating chain growth pathway for all catalysts proceeds via isomerization through **TS-1**_**Isom-E**_, forming the less stable π-complex **1-Coor-E-C**. Monomer insertion then occurs via **TS-1**_**Ins-E-C**_, leading to an intermediate stabilized by a β-agostic interaction. While **Pd-4** behaves similarly to **Pd-1** throughout the nonalternating chain growth pathway—with the determining insertion energy at about 20 kcal/mol—the TS energy for monomer insertion is reduced to 17.4 kcal/mol for **Pd-2**.^31^ This reduction is again attributed to the η^2^ interaction between the aryl ring and the metal center, which stabilizes the monomer insertion. In conclusion, the results indicate that the menthyl substituent on the phosphine moiety in **Pd-4** does not significantly influence the nonalternating reaction pathway, as the determining step’s energy closely matches that of **Pd-1**.^31^ However, the 2′,6′-dimethoxy-(1,1′-biphenyl) substituents in **Pd-2** facilitate ethylene insertion along the nonalternating pathway, as evidenced by the lower TS energy (17.4 kcal/mol for **Pd-2** vs 20.2 kcal/mol for **Pd-1**).^31^

Starting from **1-cycle5-T**, instead of ethylene coordination, carbon monoxide can coordinate via **TS-1**_**Coor-CO-T**_, initiating the alternating chain growth pathway. For **Pd-4**, the reaction overcomes an energy barrier of nearly 10 kcal/mol (close to that calculated for **Pd-1**), leading to a disfavored **1-Coor-CO-T** intermediate that suffers from steric interactions between the chain and the hindered menthyl-substituted phosphorus (see SI).^31^ The preferred pathway follows CO insertion through **TS-1**_**Ins-CO-T**_ at 17.8 kcal/mol, producing the six-membered chelate species, **1-cycle6-C**.^31^ From **1-cycle6-C**, **Pd-4** coordinates ethylene trans to the oxygen atom via **TS-2**_**Coor-E-C**_, with energy values similar to **Pd-1** (12.3 kcal/mol for **Pd-1** and 13.9 kcal/mol for **Pd-4**).^31^ The subsequent ethylene insertion occurs via **TS-2**_**Ins-E-C**_ at 7.2 kcal/mol, resulting in the favored **2-cycle5-T** intermediate. The overall free energy barrier from **1-cycle5-T** to **TS-1**_**Ins-CO-T**_ along the alternating pathway for **Pd-4** is 17.8 kcal/mol, a value comparable to **Pd-1** (16.4 kcal/mol), indicating that **Pd-4** behaves similarly to Drent’s catalyst also along the alternating pathway.^31^

**Figure 3:**
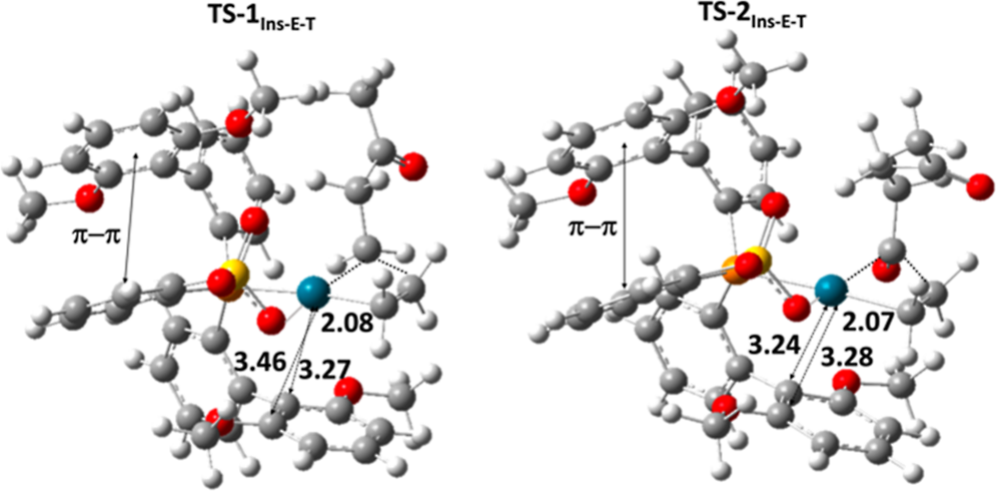
Geometries of **TS-1**_**Ins-E-T**_ and **TS-2**_**Ins-E-T**_ for **Pd-2**.

Interestingly, after a favored path (**1-Coor-CO-T** intermediate is almost 5 kcal/mol lower than the zero-point energy) through a low-energy **TS-1**_**Coor-CO-T**_, **Pd-2** instead proceeds via CO isomerization through **TS-1**_**Isom-CO-T**_ at 12.6 kcal/mol (see Scheme 2, path B). From **1-Coor-CO-C**, CO insertion proceeds via **TS-1**_**Ins-CO-C**_, producing the six-membered chelate species, **1-cycle6-T**, with a corresponding free energy barrier of 7.9 kcal/mol. As previously reported for **Pd-1**, the resulting chelate **1-cycle6-T** is highly stable also for **Pd-2**, more than 7 kcal/mol lower in energy than the initial five-membered chelate species.^31^ However, from **1-cycle6-T**, ethylene coordinates trans to the phosphorus atom via **TS-2**_**Coor-E-T**_ at only 9.5 kcal/mol, and ethylene insertion occurs via **TS-2**_**Ins-E-T**_ at 11.8 kcal/mol, yielding the stable five-membered chelate complex **2-cycle5-C**. It is noteworthy that for **Pd-1** and **Pd-4** this pathway which involves a CO isomerization step was ruled out due to the extremely high energy of the subsequent **TS-2**_**Ins-E-T**_ transition state (up to 25 kcal/mol for both catalysts, as shown in Scheme S1 for **Pd-1** and Scheme S2 for **Pd-4**). In contrast, for **Pd-2**, all ethylene insertion steps are stabilized by the interaction between the aromatic ring of the ligand and the metal. This interaction becomes stronger in **TS-2**_**Ins-E-T**_ because the metal more readily accepts electron density due to the presence of the acyl chain instead of the alkyl chain (compare distances of 3.24 and 3.28 Å for **TS-2**_**Ins-E-T**_ with 3.46 and 3.27 Å for **TS-1**_**Ins-E-T**_ in Figure 3). Even along the nonalternating pathway, the transition state for monomer insertion in the trans position to phosphorus is stabilized by this aromatic ring–metal interaction, reducing the energy to 23 kcal/mol, whereas for the other catalysts, this step requires at least 27 kcal/mol. However, for all catalysts along the nonalternating pathway, monomer insertion from the cis position to phosphorus is preferred.

Comparing the rate-determining energy barriers along the two pathways—**TS-1**_**Ins-E-C**_ vs **TS-1**_**Ins-CO-T**_ for **Pd-1** and **Pd-4** and **TS-1**_**Isom-E**_ vs **TS-2**_**Isom-CO**_ for **Pd-2**—the calculated ΔΔ*G*^⧧^(nonalt)–(alt) values are 3.8, 4.9, and 3.0 kcal/mol for **Pd-1**, **Pd-2**, and **Pd-4**, respectively.^31^ These results are consistent with experimental findings showing the formation of nonalternating keto groups and alternating motifs during pressure reactor copolymerization with these catalysts (see Table 2). The calculations suggest a slightly higher preference for the nonalternating pathway with **Pd-4** compared to **Pd-1**, which aligns with experimental results within the computational margin of error (see Table 2).^31^ On the other hand, the greater tendency of **Pd-2** to follow the less desirable alternating pathway can be attributed to electronic factors arising from its modified phosphine ligand structure. The favorable interaction between the aryl ring of the phosphine moiety and the metal stabilizes all of the key species along the reaction pathways. However, this additional remote substituent on the phosphorus donor significantly lowers the energy profile of the alternating pathway, particularly facilitating the insertion transition state when ethylene is positioned trans to phosphorus. This occurs due to a stronger stabilizing effect from the metal–ligand aryl ring interaction, ultimately enhancing the accessibility to an alternative route along the alternating pathway and reducing the overall kinetic barrier for this mechanism.

**Table 2:** Experimental Relative Ratios of Isolated, Nonalternating, and Alternating CO Incorporation Events Compared to the Theoretical ΔΔ*G*^⧧^ Obtained from DFT Calculations

catalyst	I/NA/A (%)	ΔΔ*G*^⧧^ (kcal/mol)
**Pd-1**(31)	65/31/4	3.8
**Pd-2**	60/35/5	4.9
**Pd-4**	90/10/0	3.0

### Optimization for E/CO Copolymerization to Achieve HDPE-like Copolymers

After performing the initial catalyst benchmarking in a pressure reactor setup with constant pressure, a more advanced pressure reactor setup was designed to achieve highly reproducible and automated nonalternating copolymerization performance. Key to this is to balance the highly different monomer reactivities by reliably dosing a low concentration of CO in a large excess of ethylene while always preventing a full depletion of CO. This was achieved by a software-controlled feeding system that can precisely and individually dose both monomers over a wide range of ratios and flow rates to cover all phases of the reaction. Further, this feed system allows efficient counteracting of pressure decreases by replenishing consumed monomers in the desired ratio. Thereby, a constant reactor pressure and the presence of both monomers during all reaction phases are ensured. Additionally, a quickly responding thermostat controls the internal reactor temperature (*T* ≥ 90 °C) and ensures a constant polymerization temperature even for very active polymerizations with a rapidly released heat of polymerization. For a more detailed description of the pressure reactor setup, see Supporting Information.

**Figure 4:**
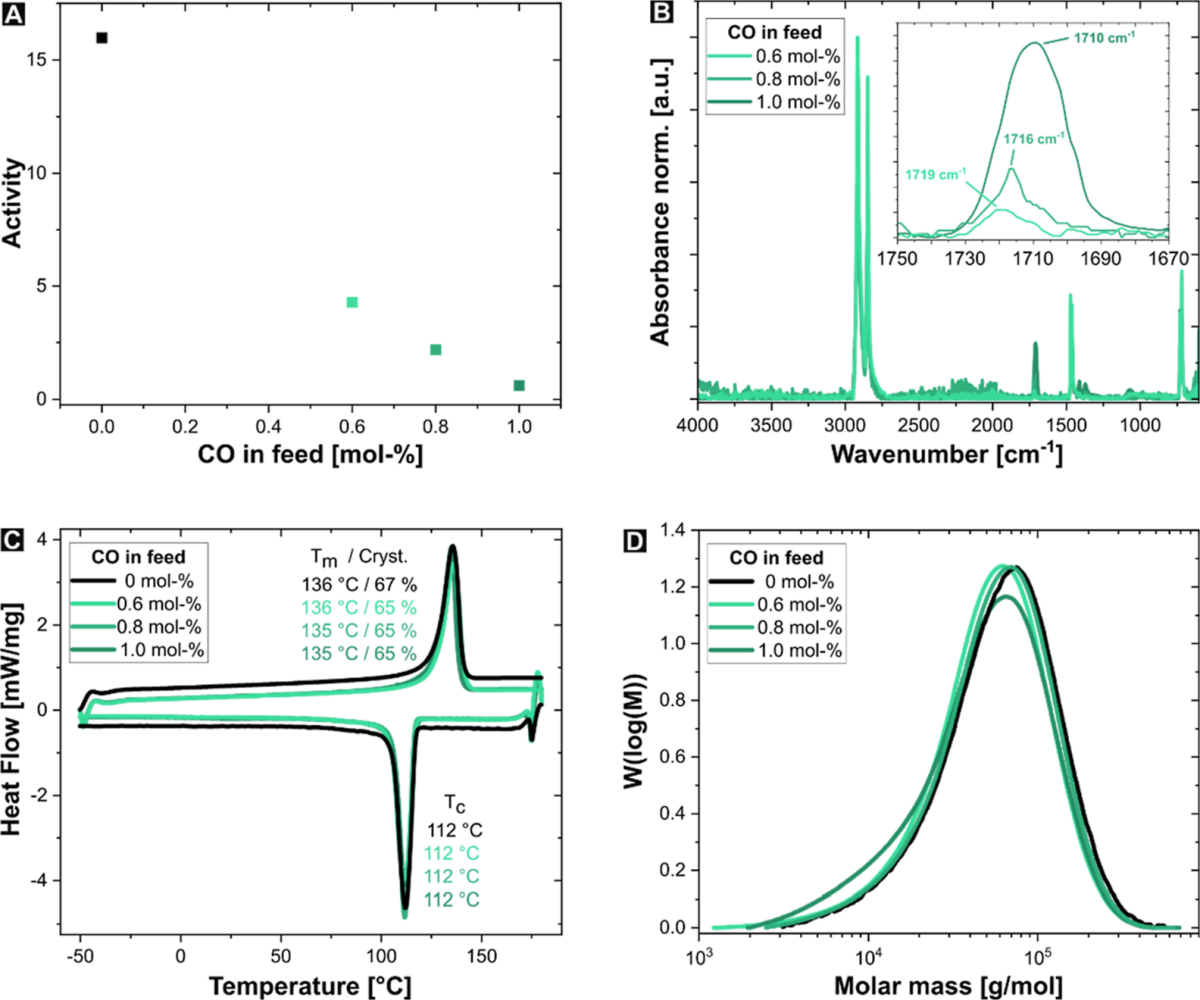
Dependence of the catalytic activity of **Pd-2** on the carbon monoxide content in the monomer feed (A), IR spectra (B), DSC curves (C), and GPC traces (D) of the copolymers **KPE5**–**KPE7**, obtained with 0.6, 0.8, and 1.0 mol % CO in the monomer feed, compared to the reference material **PE1**.

This advanced pressure reactor setup was used to further investigate the catalytic activity of **Pd-2**. The influence of carbon monoxide concentration in the monomer mixture was screened by subjecting catalyst **Pd-2** to feeds containing varying ratios of ethylene and carbon monoxide (0.6, 0.8, or 1.0 mol % CO, see Table 3). For comparison, an ethylene homopolymerization was conducted under similar conditions. The catalyst remained active in all three copolymerization experiments, although its activity and productivity gradually decreased with increasing carbon monoxide content (see Figure 4A). This result is expected, as the coordination of previously incorporated keto groups toward the metal center forms five-membered chelate intermediates (see Figure 2) which slow the polymerization compared to ethylene homopolymerization. As expected from previous studies, the amount of incorporated keto groups increased with the rising CO content in the monomer feed, as revealed by IR spectroscopy (see Figure 4B).^10^ The IR band for the C=O stretching vibration of the incorporated keto groups shifted to lower wavenumbers (1719 cm^–1^, 1716 cm^–1^, and 1710 cm^–1^, respectively) with higher CO incorporation, also indicating a decreased selectivity for isolated keto groups. This observation is further supported by ^1^H NMR spectroscopic analysis, which showed a declining proportion of isolated keto groups with an increasing amount of CO in the feed. While 94% of the keto groups were incorporated in an isolated manner in **KPE5**, only 50% of the keto groups were isolated in **KPE7**, which has a significantly higher CO content. However, the proportion of alternating motifs remains low, at just 14%, preserving the HDPE-like thermal properties. All copolymers exhibited thermal properties comparable to the reference homopolymer **PE1**, with melting points ranging from 135 to 136 °C and crystallinity around 66% (see Table 3, **KPE5**, **KPE6**, and **KPE7** and Figure 4C). While the presence of carbon monoxide in the monomer mixture led to a slight decrease in molecular weight, there was no clear correlation between the amount of CO in the feed and molecular weight (30–39 kg mol^–1^ for the copolymers compared to 41 kg mol^–1^ for **PE1**, see Figure 4D). Note that this differs from the CO response of Ni(II) phosphinophenolate catalysts, where the presence of CO results in significantly increased polymer molecular weights.

**Table 3:** Copolymerization Results of the Optimization of Conditions to Achieve HDPE-like Copolymers with Pd-2

no.	*T* (°C)	CO in the feed (mol %)	yield (g)	activity^a^	Ω^b^(mol %)	I/NA/A^c^ (%)	*M*_n_ (kg/mol) (*M*_w_/*M*_n_)^d^	*T*_m_ (°C)^e^ [cryst. (%)]
**PE1**^f^	90		3.99	15.98			41 (1.9)	136 (67)
**KPE5**	90	0.6	8.57	4.29	0.3 (0.3)	94/6/0	35 (1.9)	136 (66)
**KPE6**	90	0.8	4.38	2.19	0.7 (0.8)	90/9/1	39 (1.9)	136 (66)
**KPE7**	90	1.0	1.21	0.61	2.6 (2.0)	50/37/14	30 (2.2)	135 (66)
**KPE8**	100	0.8	4.63	2.32	1.0 (0.9)	89/10/0	23 (1.9)	134 (70)

a Copolymerization conditions: 100 mL toluene, 90 °C, 2 μmol precatalyst **Pd-2**, 1000 rpm, 10 bar, 60 min; activity given in 10^6^ g [polymer] mol^–1^ [Pd] h^–1^.

b CO incorporation determined by IR spectroscopy (CO incorporation determined by ^1^H NMR spectroscopy).

c Ratio of isolated/nonalternating/alternating keto groups in the polymer backbone, determined by ^1^H NMR spectroscopy.

d Determined by GPC in 1,2-dichlorobenzene at 160 °C linear calibration versus polyethylene standards.

e Determined by DSC (10 K min^–1^), second heating cycle.

f 0.25 μmol precatalyst **Pd-2**.

The catalyst remained active in ethylene/CO copolymerization as the temperature was increased from 90 (**KPE6**) to 100 °C (**KPE8**), leading to a slight increase in both the catalyst activity and productivity. The primary difference between the two copolymers obtained at the different temperatures is the decreased molecular weight (39 kg mol^–1^ vs 23 kg mol^–1^), this trend being expected given the higher rate of β-H elimination at elevated temperatures.^32^ The keto content increased slightly, from 0.7 to 1.0 mol %, while maintaining a high selectivity of 89% for isolated keto groups. The melting point decreased slightly for **KPE8** compared to **KPE6** (134 °C vs 136 °C), whereas the crystallinity increases marginally (70% vs 66%), both of which are attributed to the lower molecular weight.

**Figure 5:**
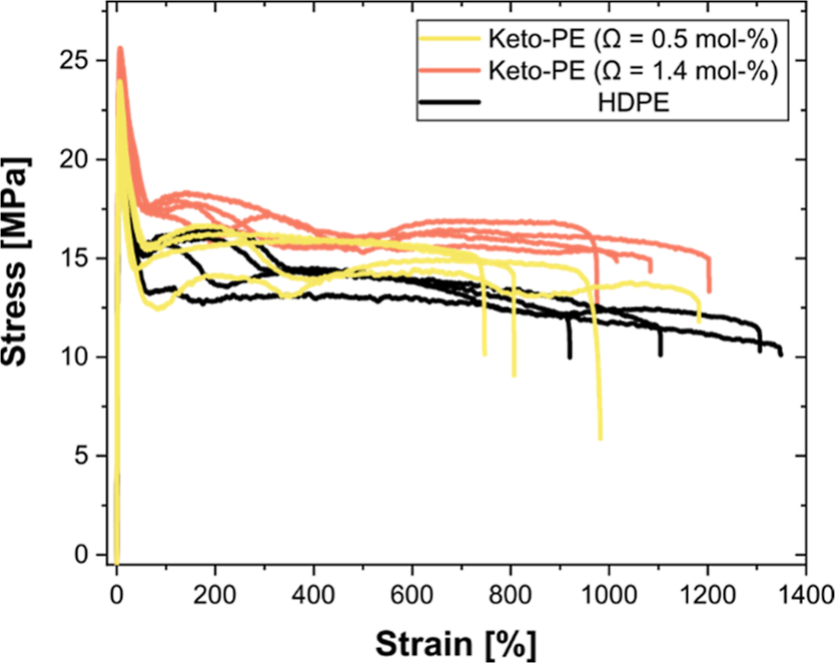
Tensile-testing results of keto-polyethylenes with 0.5 mol % and 1.4 mol % of keto groups and a high-density polyethylene reference material obtained with catalyst **Pd-2**.

Tensile tests on melt-pressed specimens of two keto-PEs, containing 0.5 and 1.4 mol % of keto groups, respectively, and with a *M*_n_ of 40 kg mol^–1^ (see Table S1 for the complete microstructure analysis), demonstrated that these materials are on par with HDPE produced with **Pd-2** under similar polymerization conditions (see Figure 5). All measured values including Young’s modulus, yield strength, elongation at yield point, and the elongation at break are, within experimental accuracy, similar to the HDPE reference (see Table S2 and Figure 5).

## Conclusions

Pd(II) phosphinosulfonate catalysts are part of a small selection of catalysts known to copolymerize ethylene and carbon monoxide in a nonalternating manner. Reports on the materials properties of the resulting polymers were so far limited to studies of brittle wax-like materials.^10^ Our combined experimental and theoretical study demonstrates that ductile HDPE-like materials properties can also be achieved with Pd(II) phosphinosulfonate catalysts, enabled by controlled feeding of the monomers. Despite the lower-energy pathway for the undesired alternating incorporation opened by the presence of the 2′,6′-dimethoxy-(1,1′-biphenyl) phosphine substituent of catalyst **Pd-2**, isolated keto motifs still prevail. This combined with the high molecular weight of the copolymer and enhanced activity of the catalyst facilitates the synthesis of the target materials. This underlines that conceptually, the catalytic synthesis of processable and ductile keto-PE materials is not limited to Ni(II) catalysis. Given the wide range of Pd-catalyzed reactions, these findings could broaden the scope of accessible materials. For example, Jian and co-workers obtained aldehyde-end-capped keto-PEs with low amounts of keto groups by introducing hydrogen into the copolymerization mixture.^33^

## Supporting Information Available

Experimental procedures, additional polymerization data, characterization data, spectra, results of tensile testing, computational details of DFT, calculations of alternative alternating path B for **Pd-1** and **Pd-4**, and Cartesian coordinates (xyz) of all calculated intermediates and transition states (PDF)
