# Supplementary material for: Keto-Polyethylene Material from Pd(II)-Catalyzed Copolymerization with Continuous Carbon Monoxide Feed
Source: ACS Catal. 2025 May 2;15(10):8259–67. doi: 10.1021/acscatal.5c00935 (PMC12090214; doi:10.1021/acscatal.5c00935)
Supplement: Supplementary file 1 — cs5c00935_si_001.pdf [file cs5c00935_si_001.pdf]

Supplementary Materials for

Keto-Polyethylene Material from Pd(II)-Catalyzed Copolymerization with Continuous Carbon Monoxide Feed

Steffen Iberl[‡,§], Maria Voccia[†,§], Ida Ritacco[†], Lukas Odenwald[‡], Maximilian Baur[‡], Laura Falivene[†], Lucia Caporaso[†,*] and Stefan Mecking[‡,*].

[‡] Chair of Chemical Material Science, Department of Chemistry, University of Konstanz, 78464 Konstanz, Germany.

[†] Department of Chemistry, University of Salerno, 848048 Fisciano, Salerno, Italy.

* Corresponding authors: Stefan Mecking, stefan.mecking@uni-konstanz.de and Lucia Caporaso, lcaporaso@unisa.it.

Contents

1. Experimental Procedures 3
 1.1 General Considerations 3
  1.1.1 Solvents and Reagents 3
  1.1.2 Analytical Methods 3
 1.2 Synthesis of the Phosphine Sulfonate Pd(II) Catalysts 5
  1.2.1 General Procedure for the Precatalyst Synthesis 5
  1.2.2 NMR Characterization of **Pd-1** 5
  1.2.3 NMR Characterization of **Pd-2** 7
  1.2.4 NMR Characterization of **Pd-3** 8
  1.2.5 NMR Characterization of **Pd-4** 10
 1.3 Reactor Setup for Preliminary Catalyst Screening 11
 1.4 Reactor Setup for Convenient Non-Alternating Ethylene/CO Copolymerization 11
 1.5 General Polymerization Procedures 13
2. Additional Polymerization Data 14
3. Characterization of Polyethylenes and Ethylene/Carbon Monoxide Copolymers 15
 3.1 Infrared Spectroscopy 15
 3.2 Nuclear Magnetic Resonance Spectroscopy 16
  3.2.1 Exemplary ^{1}H-NMR Spectrum of a Polyethylene 16
  3.2.2 Exemplary ^{1}H-NMR Spectrum of a Keto-Polyethylene 17
  3.2.3 Calculation of Molecular Weight *via* ^{1}H-NMR Spectroscopy 17
  3.2.4 Microstructure Analysis *via* ^{1}H-NMR Spectroscopy 18
 3.3 Gel-Permeation Chromatography 21
 3.4 Differential Scanning Calorimetry 22
4. Tensile Testing 23
5. Density Functional Theory 24
 5.1 Computational Details 24
 5.2 Alternative Alternating Pathway for **Pd-1** and **Pd-4** 24
 5.3 Steric analysis of **1-Coor-CO-T** for **Pd-4** 25
 5.4 Cartesian Coordinates 26
6. References 59

1. Experimental Procedures

1.1 General Considerations

All manipulations of oxygen or moisture sensitive compounds were performed under a nitrogen inert gas atmosphere using conventional SCHLENK or glovebox techniques.

1.1.1 Solvents and Reagents

Solvents were dried and degassed using standard laboratory techniques. THF was distilled from sodium benzophenone ketyl, benzene from sodium and pyridine from CaH_2. Pentane, dichloromethane and toluene were dried and degassed by passing through columns equipped with alumina and BASF R3-11 catalyst. Acetonitrile was supplied from VWR Chemicals and dried over molecular sieves (4 Å) overnight. TMEDA was sourced from Thermo Fisher Scientific and distilled from KOH. $PdCl_2$ was generously provided by Umicore as a highly subsidized material. 1.6 M MeLi in diethyl ether and 2.5 M n-BuLi in hexanes were obtained from ThermoFisher Scientific. Carl Roth provided methyl t-butyl ether and anhydrous Na_2SO_4. All other commercially available reagents and starting materials were purchased from Merck. $CDCl_3$ was supplied by Merck, and all other deuterated solvents were obtained from Eurisotop. Ethylene (grade 3.5) and carbon monoxide (grade 4.7) were purchased from Air Liquide and used as received.

1.1.2 Analytical Methods

NMR spectra were recorded either on a Bruker Avance III HD 400 (^{1}H-NMR: 400 MHz, ^{13}C-NMR: 101 MHz, ^{31}P-NMR: 162 MHz), a Bruker Avance III 400 (^{1}H-NMR: 400 MHz, ^{13}C-NMR: 101 MHz, ^{31}P-NMR: 162 MHz) or on a JEOL JNM-ECZ500R (^{1}H-NMR: 500 MHz, ^{13}C-NMR: 126 MHz, ^{31}P-NMR: 202 MHz). ^{1}H-NMR chemical shifts were referenced to the solvent's residual proton signals ($CDCl_3$: 7.26 ppm, CD_2Cl_2: 5.32 ppm, C_6D_6: 7.16 ppm, $C_2D_2Cl_4$: 6.00 ppm). ^{13}C-NMR spectra were referenced to the carbon signal of the deuterated solvent. ^{31}P-NMR was referenced to an internal standard. All NMR spectra of polyethylene and keto-polyethylene materials were obtained using 1,1,2,2-tetrachloroethane-d_2 as solvent at 373 K or 383 K. MestReNova software (version 14.2.0-26256) by Mestrelab Research S.L. was used for data evaluation.

ATR-IR spectroscopy of the keto-modified polyethylenes was measured on a Perkin Elmer Spectrum 100 instrument. The Perkin Elmer Spectrum IR software (version 10.7.2.1630) was used for interactive baseline correction. The carbon monoxide content was calculated from the IR spectra by the ratio of the integral of the C=O signal (integration area: 1651-1770 cm^{-1}, depending on the baseline of the spectrum, usually peak of the C=O signal ± 65 cm^{-1}) and the integral of the C-H signal of the polyethylene backbone (integration area: 2740-3030 cm^{-1}). The obtained ratio was referenced against linear polyketones with a known carbon monoxide incorporation which were obtained *via* ADMET copolymerization and hydrogenation of the obtained unsaturated polyketone (see Figure S1).[1] Origin 2022 (version 9.9.0.225) was used for the integration of the IR signals.

GPC was measured on a PolymerChar GPC-IR instrument equipped with PSS Polefin Linear XL columns (3 x 30 cm), a viscometer and an IR-dual wavelength detector (for methylene and methyl units) in 1,2-dichlorobenzene with a flow rate of 1 mL min^{-1} at 160 °C.

DSC was measured on a DSC 204 F1 manufactured by Netzsch (software: Netzsch Proteus Thermal Analysis, version 6.1.0) with a bicyclic temperature program from -50 to 180 °C with heating and cooling rates of 10 K min^{-1} in closed 40 µL alumina pans under a nitrogen atmosphere. Data reported are from second heating cycles.

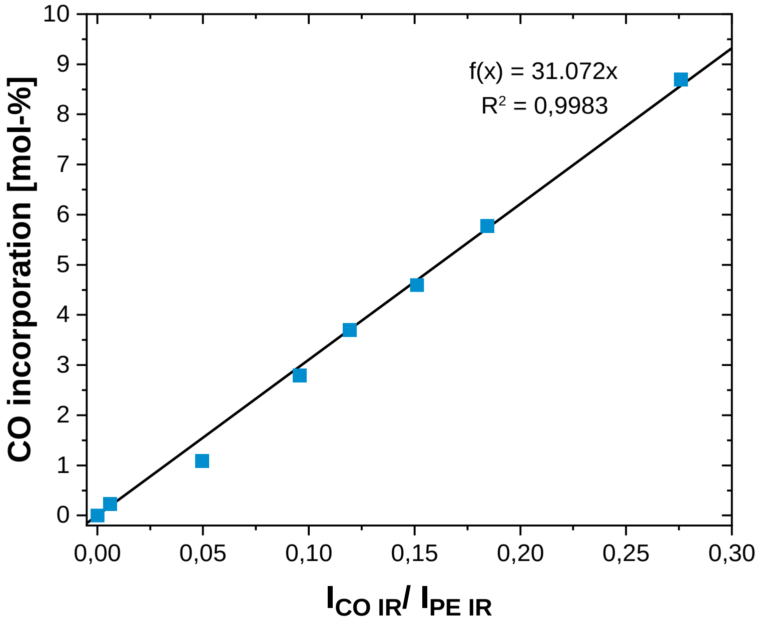

Figure S1. Calibration curve for the calculation of the keto content of keto-polyethylenes from IR spectroscopy, calibration obtained from linear polyketones with a known carbon monoxide incorporation which were obtained via ADMET copolymerization and hydrogenation of the obtained unsaturated polyketone.[1]

A custom-made mold with stamps was used to produce melt-pressed tensile testing specimens according to DIN EN ISO 527-2, type 5B (see Figure S2, A and B). 200 mg of precipitated polymer were placed into each mold and the polymer was compressed with a hammer to prevent air bubbles in the specimen. The mold was then sealed with a corresponding stamp and Teflon tape. A metal plate was placed on top and screw clamps were used to further compress the polymer. The screws were tightened by hand followed by and additional turn with a wrench. The entire setup was placed in a custom-made reactor setup, which was then sealed, evacuated and placed into a preheated heating block at 160 °C for two hours. After this period, the setup was allowed to cool down slowly overnight.

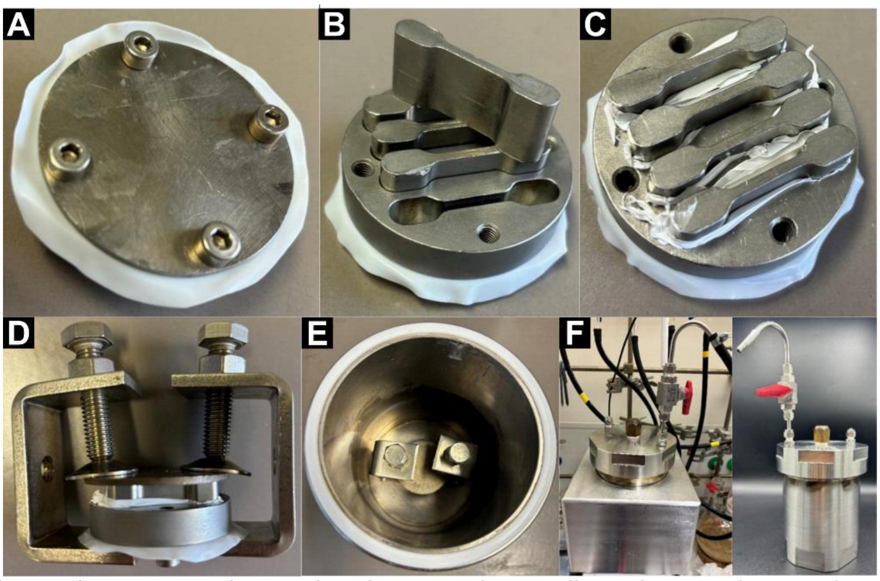

Figure S2. Melt-pressing setup and procedure for preparing tensile testing specimens: A) Mold preparation, B) image of the mold with tensile specimen-shaped stamps, C) mold after filling with polymer, D) mold with applied pressure after clamp closure, E) mold placed inside a stainless-steel reactor and F) reactor sealed and positioned in a heating block.

Before tensile testing with a Zwick Z005/1446 Retroline tC II, all samples were preconditioned at room temperature. A crosshead speed of 5 mm min^{-1} was used for tensile testing of the injection-molded samples (ISO 527-2, type 5A). The Young's modulus was measured at a crosshead speed of 0.5 mm min^{-1}. The Zwick Roell testXpert software version 11.0 was used for data evaluation.

1.2 Synthesis of the Phosphine Sulfonate Pd(II) Catalysts

1.2.1 General Procedure for the Precatalyst Synthesis

All phosphine sulfonate ligands and the corresponding Pd(II) precatalysts were synthesized according to literature-known procedures: **Pd-1**[2], **Pd-2**[3–5], **Pd-3**[6] and **Pd-4**[7]. [(tmeda)$PdMe_2$] which was required as Pd(II) precursor for the syntheses of all precatalysts was obtained from $PdCl_2$ according to literature.

In a glove box, 100 mg of the respective phosphine sulfonate ligand (1.0 equiv.) and [(tmeda)$PdMe_2$] (1.1 equiv.) were dissolved in 2 mL of anhydrous benzene. Anhydrous pyridine (20 equiv.) were added and the solution was stirred for 90 minutes. The mixture was lyophilized, yielding a colorless powder whose purity was checked by ^{1}H- and ^{31}P-NMR analysis. If NMR analysis showed impurities (typically pyridine and/or [(tmeda)$PdMe_2$]), the catalyst was washed with pentane and then redissolved in benzene and lyophilized again. This yielded the catalysts as pure, colorless powder in good yields.

1.2.2 NMR Characterization of **Pd-1**

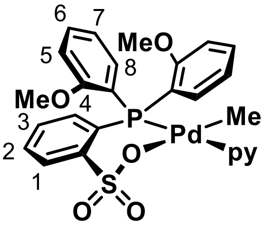

Pd-1

*Figure S3. Structure of precatalyst **Pd-1**.*

^{1}H-NMR (400 MHz, CD_2Cl_2, 300 K) δ [ppm]: 8.76 (d, 2H, $^{3}J_{HH}$ = 5.4 Hz, *o*-py), 8.06 (dd, 1H, $^{3}J_{HH}$ = 7.8 Hz, $^{4}J_{HH}$ = 4.7 Hz, H-1), 7.88 (t, 1H, $^{3}J_{HH}$ = 7.6 Hz, *p*-py), 7.67 – 7.58 (m, 2H, H-6), 7.54 (t, 2H, $^{3}J_{HH}$ = 7.8 Hz, *m*-py), 7.51 – 7.43 (m, 3H, H-2, H-8), 7.35 – 7.26 (m, 2H, H-3, H-4), 7.03 (t, 2H, $^{3}J_{HH}$ = 7.5 Hz, H-7), 6.98 (dd, 2H, $^{3}J_{HH}$ = 8.3, $^{4}J_{HH}$ = 4.7 Hz, H-5), 3.66 (s, 6H, -OMe), 0.24 (d, 3H, $^{3}J_{PH}$ = 2.8 Hz, Pd-CH_3).

^{31}P-NMR (162 MHz, CD_2Cl_2, 300 K) δ [ppm]: 21.4.

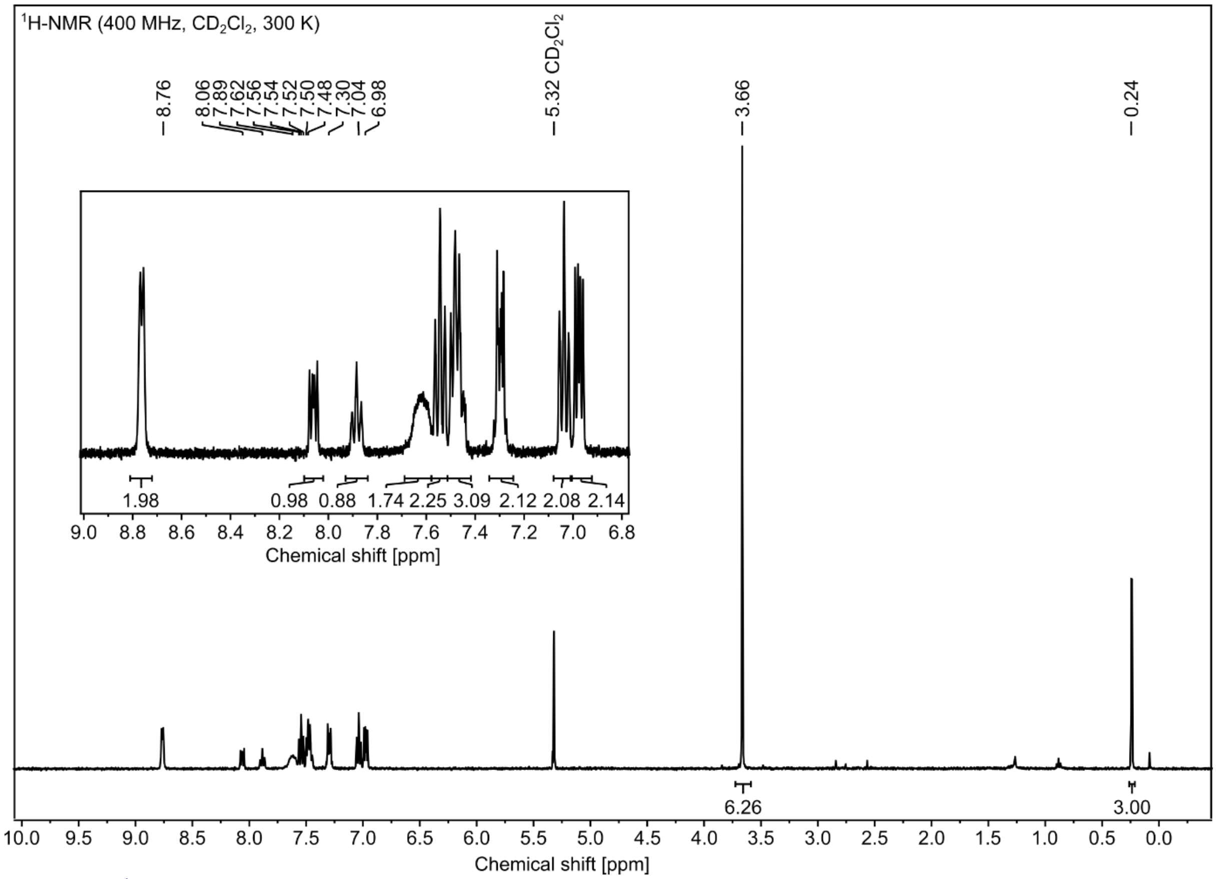

*Figure S4. ^{1}H-NMR spectrum of **Pd-1** (400 MHz, CD_2Cl_2, 300 K).*

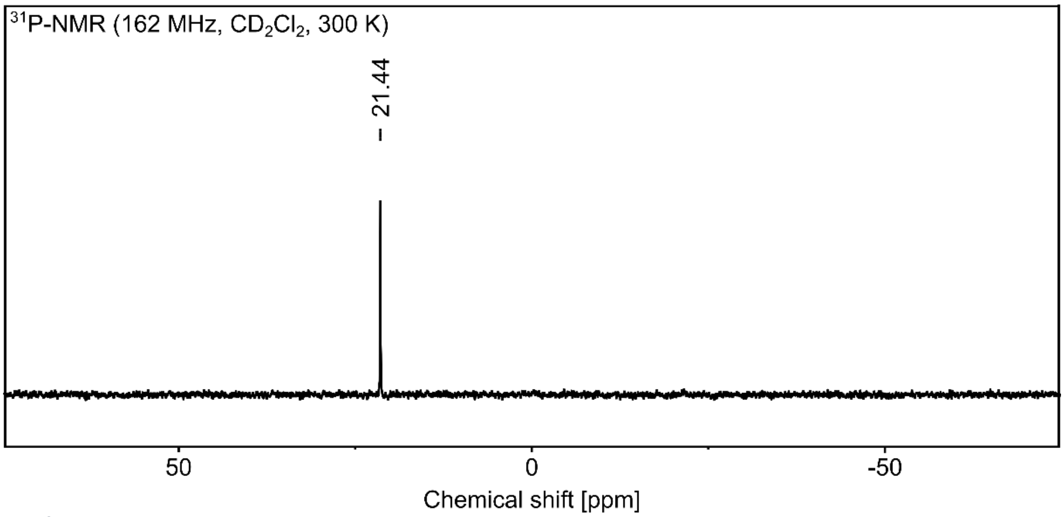

*Figure S5. ^{31}P-NMR spectrum of **Pd-1** (162 MHz, CD_2Cl_2, 300 K).*

1.2.3 NMR Characterization of **Pd-2**

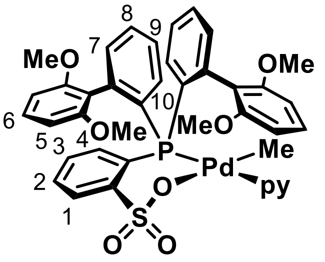

Pd-2

*Figure S6. Structure of precatalyst **Pd-2**.*

^{1}H-NMR (500 MHz, CD_2Cl_2, 298 K) δ [ppm]: 8.54 – 8.50 (m, 2 H, *o*-pyr), 7.83 (tt, $^3J_{HH}$ = 7.6 Hz, $^4J_{HH}$ = 1.6 Hz, 1 H, *p*-pyr), 7.77 – 7.70 (m, 3 H, H-1, H-10), 7.47 – 7.39 (m, 5 H, H-4, H-8, *m*-pyr), 7.33 (tt, $^3J_{HH}$ = 7.7 Hz, $^3J_{HH}$ = 1.5 Hz, H-9), 7.16 (tt, $^3J_{HH}$ = 7.6 Hz, $^4J_{HH}$ = 1.4 Hz, 1 H, H-2), 7.10 (ddd, $^3J_{HH}$ = 7.7 Hz, $^3J_{HH}$ = 4.4 Hz, $^4J_{HH}$ = 1.5 Hz, 2 H, H-7), 7.02 – 6.95 (m, 3 H, H-3, H-6), 6.37 (d, $^3J_{HH}$ = 8.2 Hz, 2 H, H-5), 6.23 (d, $^3J_{HH}$ = 8.3 Hz, 2 H, H-5), 3.61 (s, 6 H, OMe), 3.34 (s, 6 H, OMe), 0.18 (d, $^3J_{HH}$ = 2.2 Hz, 3 H, Pd-Me).

^{31}P-NMR (202 MHz, CD_2Cl_2, 298 K) δ [ppm] 21.3.

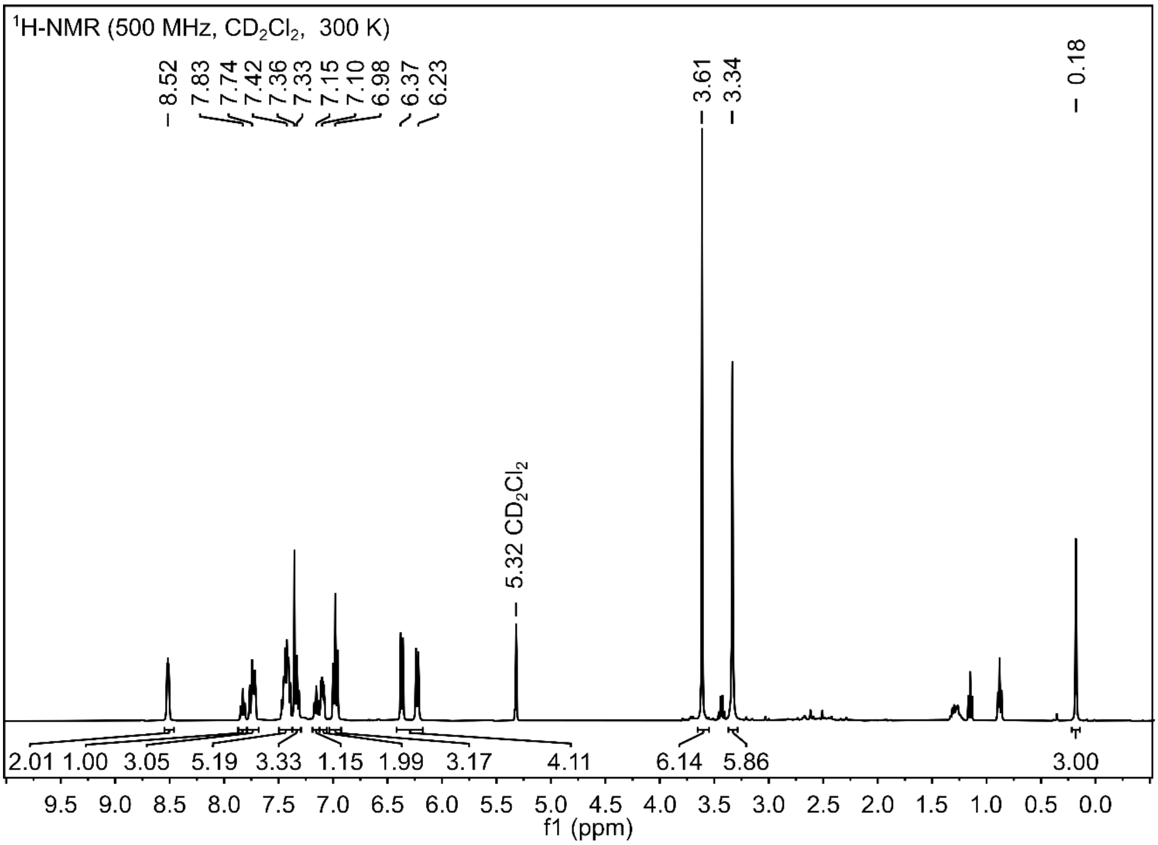

*Figure S7. ^{1}H-NMR spectrum of **Pd-2** (500 MHz, CD_2Cl_2, 300 K).*

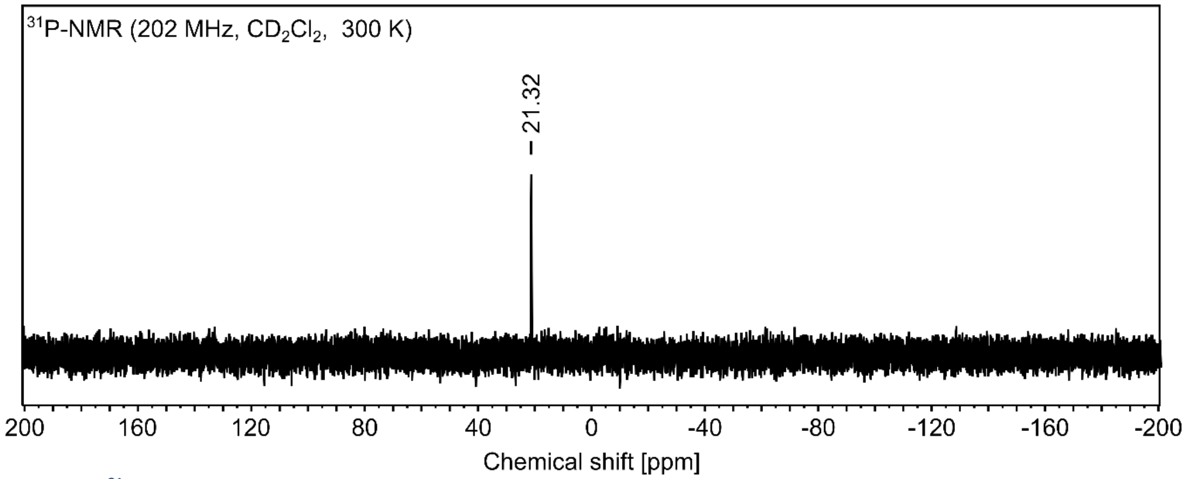

*Figure S8. ^{31}P-NMR spectrum of **Pd-2** (202 MHz, CD_2Cl_2, 300 K).*

1.2.4 NMR Characterization of **Pd-3**

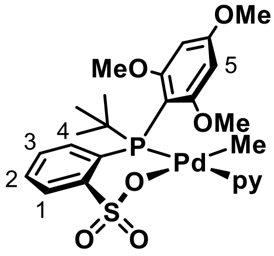

Pd-3

*Figure S9. Structure of precatalyst **Pd-3**.*

^{1}H-NMR (400 MHz, CD_2Cl_2, 300 K) δ [ppm]: 8.85 (vd, 2H, $^{3}J_{HH}$ = 4.3, *o*-py), 8.15 (dd, 1H, $^{3}J_{HH}$ = 8.0 Hz, $^{4}J_{HH}$ = 4.4 Hz, H-1), 7.86 (t, 1H, $^{3}J_{HH}$ = 7.7 Hz, *p*-py), 7.52 – 7.39 (m, 3H, *m*-py, H-2), 7.26 – 7.19 (m, 2H, H-3, H-4), 6.19 (s, 1H, H-5), 6.04 (s, 1H, H-5), 3.89 (s, 3H, *p*-OMe), 3.81 (s, 3H, , *o*-OMe), 3.45 (s, 3H, *o*-OMe), 1.49 (d, 9 H, $^{3}J_{PH}$ = 16.1 Hz, -tBu), 0.17 (m, 3H, $^{3}J_{PH}$ = 2.7 Hz, Pd-Me).

^{31}P-NMR (162 MHz, CD_2Cl_2, 300 K) δ [ppm]: 43.5.

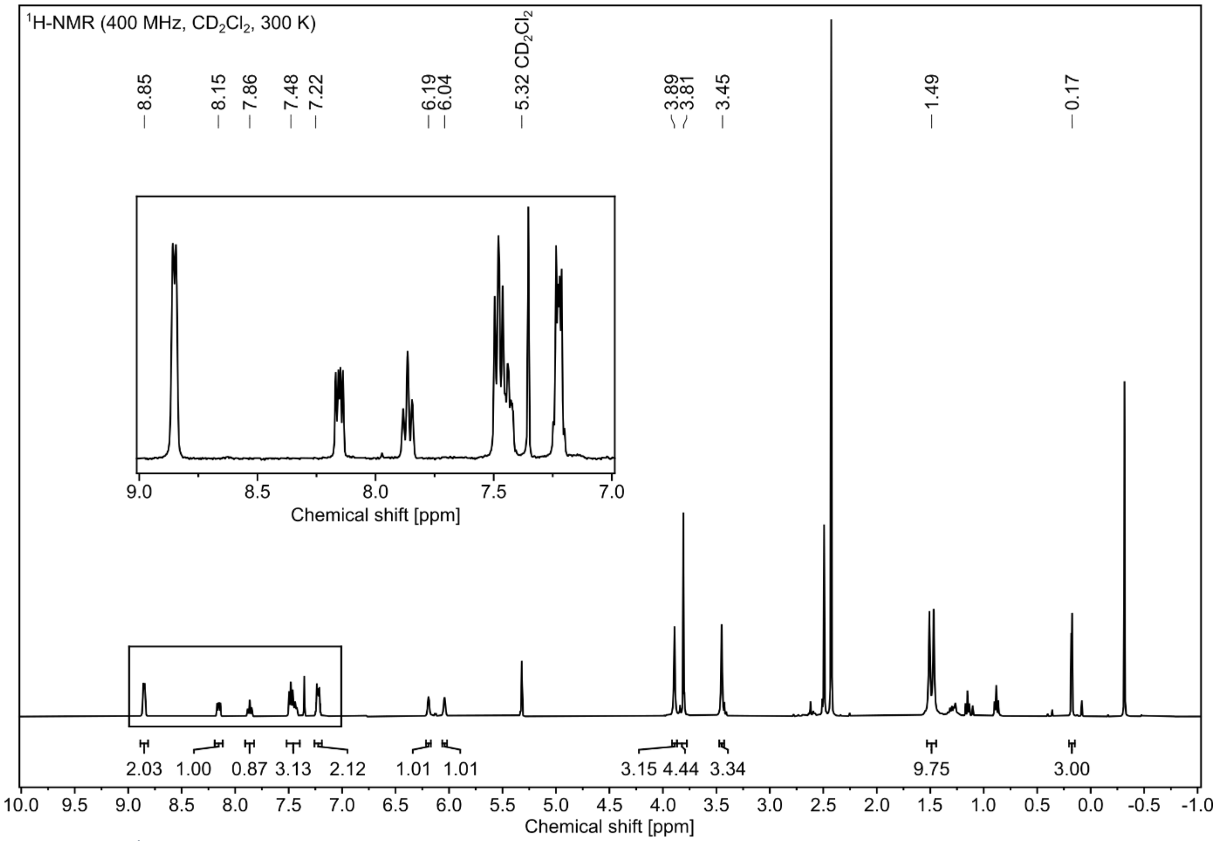

*Figure S10. ^{1}H-NMR spectrum of **Pd-3** (400 MHz, CD_2Cl_2, 300 K).*

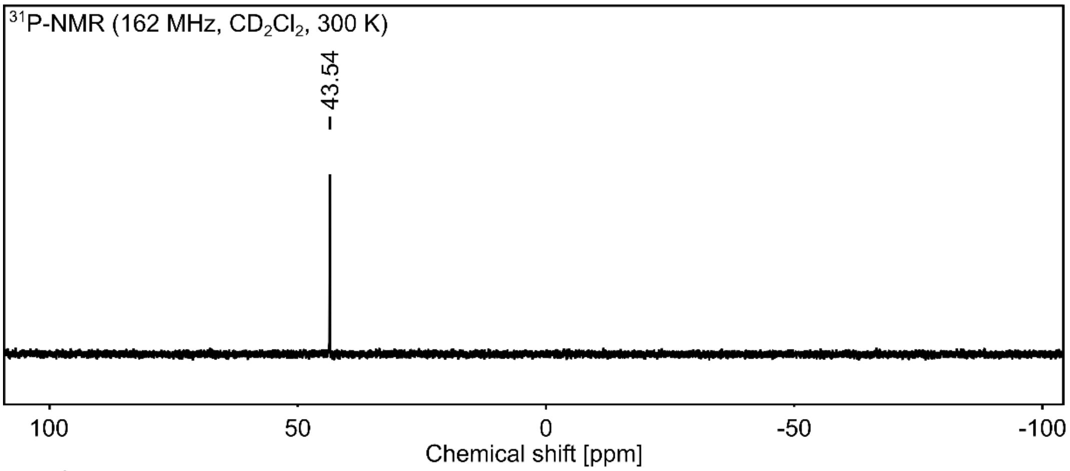

*Figure S11. ^{31}P-NMR spectrum of **Pd-3** (162 MHz, CD_2Cl_2, 300 K).*

1.2.5 NMR Characterization of **Pd-4**

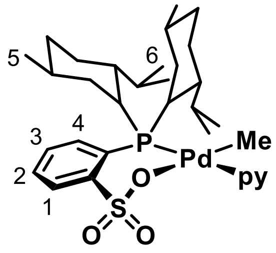

Pd-4

*Figure S12. Structure of precatalyst **Pd-4**.*

^{1}H-NMR (400 MHz, CD_2Cl_2, 300 K) δ [ppm]: 8.78 (dt, 2H, $^{3}J_{HH}$ = 6.5 Hz, 1.8 Hz, *o*-py), 8.19 (dd, 1H, $^{3}J_{HH}$ = 7.6 Hz, $^{4}J_{HH}$ = 4.0 Hz, H-1), 7.89 (tt, 1H, $^{3}J_{HH}$ = 7.7, $^{4}J_{HH}$ = 1.7 Hz, *p*-py), 7.84 (td, 1H, $^{3}J_{HH}$ = 7.9, $^{4}J_{HH}$ = 1.5 Hz, H-2), 7.56 – 7.44 (m, 4H, *m*-py, H-3, H-4), 4.25 (s, 1H, $H_{Menthyl}$), 2.59 – 2.46 (m, 1H, $H_{Menthyl}$), 2.40 (t, 1H, *J* = 11.1 Hz, $H_{Menthyl}$), 2.20 – 2.15 (m, 1H, $H_{Menthyl}$), 2.08 (p, 1H, *J* = 7.0 Hz, $H_{Menthyl}$), 2.02 – 1.94 (m, 1H, $H_{Menthyl}$), 1.86 – 1.75 (m, 3H, $H_{Menthyl}$), 1.71 - 1.60 (m, 2H, $H_{Menthyl}$), 1.55 – 1.24 (m, 3H, $H_{Menthyl}$), 1.24 – 1.00 (m, 4H, $H_{Menthyl}$),0.97 (d, 3H, $^{3}J_{HH}$ = 6.3 Hz, H-5 or H-6), 0.94 (d, 3H, $^{3}J_{HH}$ = 6.3 Hz, H-5 or H-6), 0.84 (d, 3H, $^{3}J_{HH}$ = 6.7 Hz, H-5 or H-6), 0.81 – 0.76 (m, 6H, H-5 or H-6), 0.65 (d, 3H, $^{3}J_{HH}$ = 2.1 Hz, H-5 or H-6), 0.06 (d, 3H, $^{3}J_{HH}$ = 6.7 Hz, Pd-Me).

^{31}P-NMR (162 MHz, CD_2Cl_2, 300 K) δ [ppm]: 21.2.

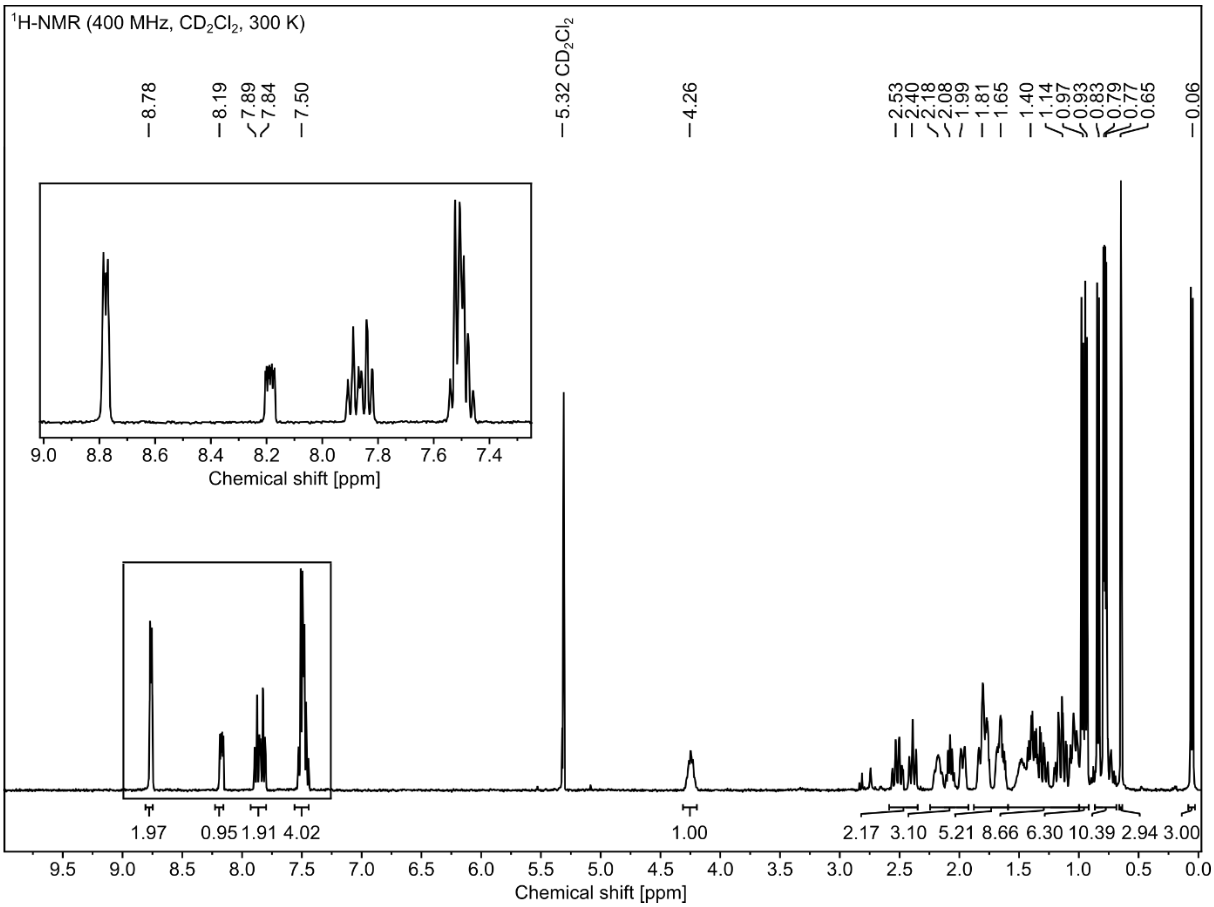

*Figure S13. ^{1}H-NMR spectrum of **Pd-4** (400 MHz, CD_2Cl_2, 300 K).*

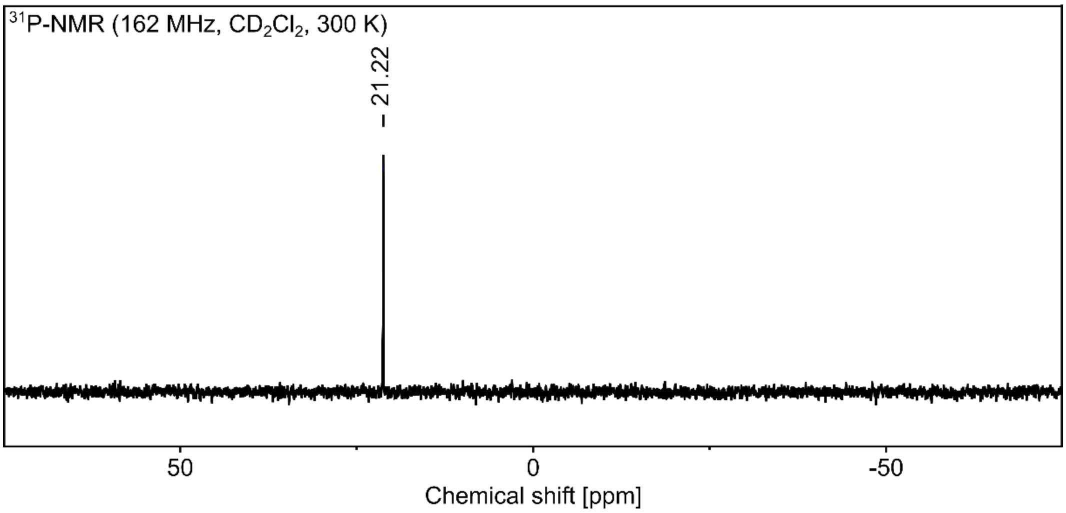

*Figure S14. ^{31}P-NMR spectrum of **Pd-4** (162 MHz, CD_2Cl_2, 300 K).*

1.3 Reactor Setup for Preliminary Catalyst Screening

Preliminary ethylene-carbon monoxide copolymerizations were conducted in a Büchi Ecoclave® reactor with a 600 mL vessel. The reactor was equipped with a heating and cooling jacket connected to a thermostat, a mechanical stirrer (Büchi Cyclone c075dc) with a three-blade agitator, a nitrogen/vacuum supply, a thermocouple dipping into the reaction mixture controlling the thermostat and a liquid dosing Pump (Knauer P 4.1s). A Bronkhorst MassFlow apparatus consisting of two flow meters (up to 20 g L^{-1} and 200 g L^{-1} ethylene), a pressure meter and a compressed air-driven badger valve was used to work under constant pressure. All gas valves and devices were connected to a HiTec Zang LabBox and operated by HiTec Zang LabVision® software (ver. 2.13). Prior to all polymerization experiments, the reactor was evacuated and heated (thermostat temperature: 90 °C). When the internal reactor temperature exceeded 60 °C, the reactor was flushed with nitrogen and evacuated three times. Then the temperature was adjusted to the desired reaction temperature. 200 mL of dry and degassed toluene were added, stirring was started with 100 rpm and the system was equilibrated for 5 min to reach the desired (internal) temperature. The system was pressurized with ^{13}C-labeled carbon monoxide (starting from between 0.99 to 1.02 bar nitrogen pressure, adding the desired amount of carbon monoxide). The CO supply was disconnected, and the system was pressurized to a total pressure of 10 bar using an ethylene mass flow regulator. The precatalyst was dissolved in 4 mL of dichloromethane and added to the reactor via the liquid dosing pump with 10 mL per minute. The reaction time was started upon begin of addition. The tubing of the pump was flushed with toluene for 3 min to assure the complete addition of the precatalyst. The reaction was kept under constant pressure for the given reaction time and then the pressure was released. The reaction mixture was added to 600 ml of MeOH, and the precipitated polymer was filtered off. The residue was washed with methanol and then dried at 60 °C/30 mbar overnight.

1.4 Reactor Setup for Convenient Non-Alternating Ethylene/CO Copolymerization

Key factor to control the copolymerization of ethylene and carbon monoxide (CO) in a non-alternating fashion, is to consider and balance the highly different monomer reactivity ratios.[8,9] Due to its stronger binding affinity to the metal center, CO is preferred for insertion during the catalytic cycle. This is compensated by employing only a very low ratio of CO in a large excess of ethylene. Additionally, high polymerization temperatures (> 80 °C) kinetically compensate for the difference in coordination[9] and insertion affinities of CO and ethylene.[10,11] On the other hand, the polymerization rate is reduced by the presence of already small amounts of CO,

when compared to only ethylene homopolymerization.[2,12,13] CO depletion would lead to a sudden increase in exothermic polymerization activity and result in uncontrolled reaction conditions. Therefore, it is important to ensure the presence of CO and avoid CO depletion at all times during the copolymerization. Therefore, a continuous and responsive monomer supply with a controlled and low amount of CO is necessary to replenish consumed monomers indicated by a decrease in reaction pressure. One method to ensure constantly low amounts of CO is the use of chemical CO surrogates, for example methyl formate or metal carbonyls, which were successfully used for ethylene/CO copolymerization.[14–16] We have demonstrated previously, that the use of a gas-feed mix with < 1 mol% CO in an high flow of ethylene can be used for achieving desired copolymerization conditions without the need for a chemical CO-surrogate.[12,17] Here, continuously feeding premixed CO and ethylene gas replenished consumed monomers and enabled polymerization times of up to 4 h.[12]

An advanced copolymerization setup (see Figure S15 and Figure S16) was developed which can dose both monomers individually over a wide range of ratios and flow rates to cover all phases of the reaction. This consists of a Büchi Miniclave® Drive autoclave with a 300 mL stainless steel vessel equipped with a Cyclone 75D magnetically coupled pitched blade stirrer. Reactor temperature is constantly monitored via software automatization with LabVision® by Hitec Zhang (TIR) and controlled by a Julabo CF41 Thermostat. The continuous feed of monomers is enabled by two separate monomer feed streams which only combine before entering the reactor vessel, thus providing high flexibility in CO/ethylene ratios. Each monomer feed line comprises of two parallel operating El-Flow® mass flow controllers by Bronkhorst (FIRC1 – FIRC4). One to control high flow rates and the other to ensure exact dosing at low flow rates of the respective monomer gas. Fully automized input for the mass flow controllers is given by Hitec Zhang's LabVision software, which constantly monitors the internal reactor pressure (PIRC) and rapidly responds to any pressure changes by adjusting the flow rates. This advanced copolymerization setup allows to maintain stable pressure, CO monomer content and reactor temperature at all times during a copolymerization. An optional connection to the CO-mass flow controllers enables further input to the monomer gas feed, such as conveniently switching to ^{13}CO, which can then be used for labelling under identical polymerization conditions.

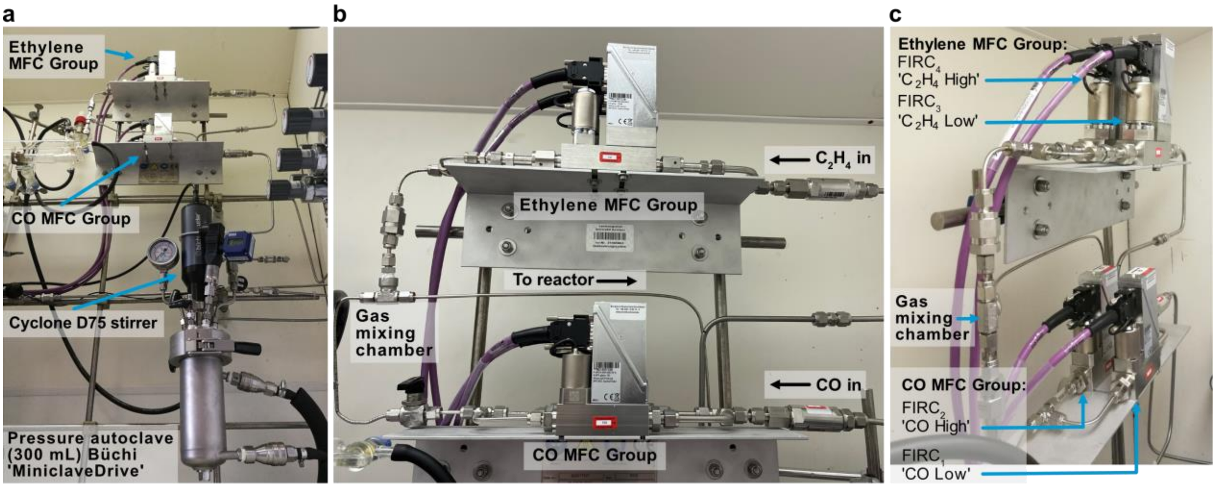

*Figure S15. Main components of the copolymerization reactor setup. **a**: Full view on the setup showing the reactor vessel and the two mass flow control groups (MFCs) above. **b**: Front view on the two MFC groups for gas supply (top: ethylene; bottom: CO). **c**: Side view on the two MFC groups, showing all four individual MFCs ($FIRC_{1-4}$).*

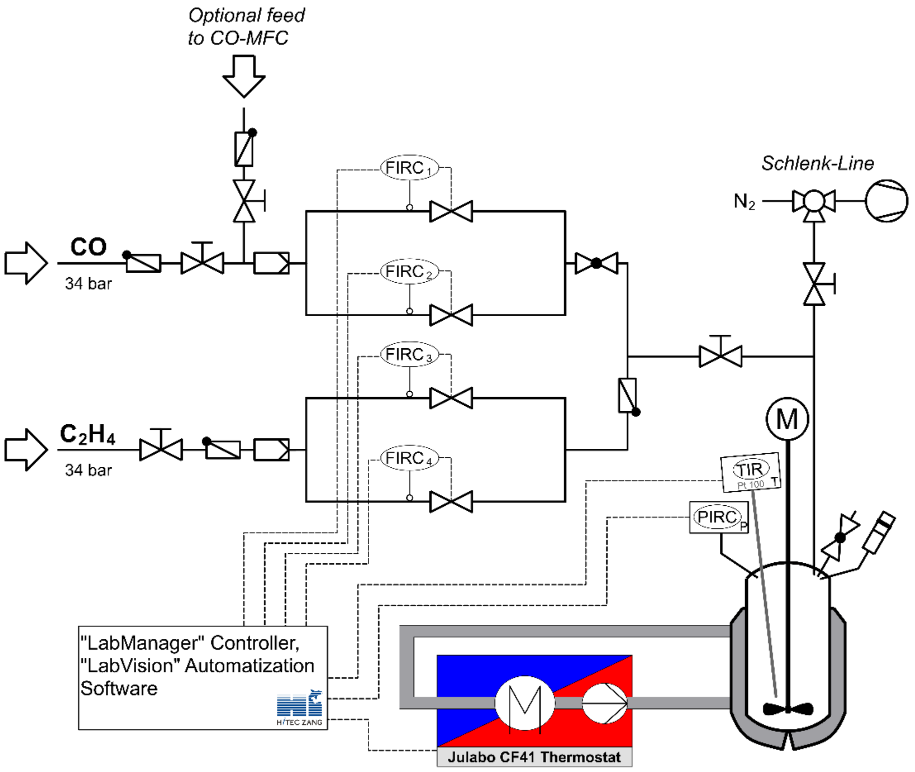

Figure S16. Piping- and instrumentation diagram (P&ID) of an advanced copolymerization setup. The setup consists of a Miniclave Drive reactor by büchiglasuster®, connected to a Julabo CF41 thermostat and equipped with a gas feed system of four individually operating EL-FLOW® mass flow controllers (MFC) by Bronkhorst®. HiTec Zang's LabManager® collects all data and autonomously controls the monomer gas feeds and temperature. (FIRC: flow indicator with controlling function, PIRC: pressure indicator with controlling function, TIRC: temperature indicator with controlling function).

1.5 General Polymerization Procedures

Before each polymerization, the reactor was evacuated and heated to 104 °C for 90 minutes, with nitrogen flushing and re-evacuation every 30 minutes. Subsequently, the reactor was set to the desired copolymerization temperature. To remove any residual monomer gas mixture from previous polymerizations, the pipe connecting the reactor to the MFCs was evacuated and flushed with nitrogen three times before introducing 100 mL of toluene into the reactor *via* cannula. The required amount of precatalyst was dissolved in 3 mL of toluene and then added to the reactor. Once stirring of the polymerization mixture (at 1000 rpm) commenced, the automated copolymerization protocol was initiated, which pressurizes the reactor and continuously doses the monomer mixture whenever the pressure falls below the desired threshold. After copolymerization, the reactor was vented and the reaction mixture was poured into approximately 700 mL of methanol, filtered, washed with methanol, and dried overnight in a vacuum drying cabinet.

2. Additional Polymerization Data

Table S1. Results of additional polymerizations performed to obtain material for processing and perform tensile testing.

#	T [°C]	Yield [g]	Activity[a]	Ω[b] [mol-%]	I/NA/A[c] [%]	M_n [kg/mol] (M_w/M_n)[d]	T_m [°C][e] (Cryst. [%])
**KPE-S1**	90	2.34	1.17	1.4 (1.0)	73 / 23 / 4	40 (1.8)	134 (64)
**KPE-S2**	90	4.17	2.09	0.5 (0.8)	82 / 16 / 2	42 (1.7)	135 (57)

Polymerizations conditions: 100 mL toluene, 90 °C, 2 µmol precatalyst **Pd-2**, 1000 rpm, 10 bar, 0.8 mol-% of CO in the feed, 60 minutes; a) in 10^6 g[polymer] mol^{-1}[Pd] h^{-1}; b) CO incorporation determined by IR spectroscopy (CO incorporation determined by ^{1}H-NMR spectroscopy); c) ratio of isolated/non-alternating/alternating keto groups in the polymer backbone, determined by ^{1}H-NMR spectroscopy; d) determined by GPC in 1,2-dichlorobenzene at 160 °C linear calibration versus polyethylene standards; e) determined by DSC (10 K min^{-1}), second heating cycle.

3. Characterization of Polyethylenes and Ethylene/Carbon Monoxide Copolymers

3.1 Infrared Spectroscopy

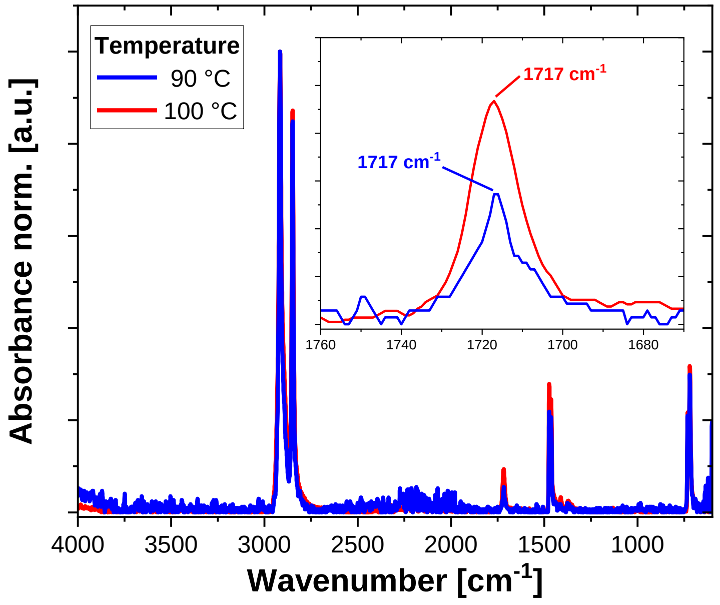

*Figure S17. IR spectra of two keto-polyethylenes obtained with **Pd-2** at 10 bar and with 0.8 mol-% of CO in the feed gas at 90 °C (blue, **KPE6**) and 100 °C (red, **KPE8**).*

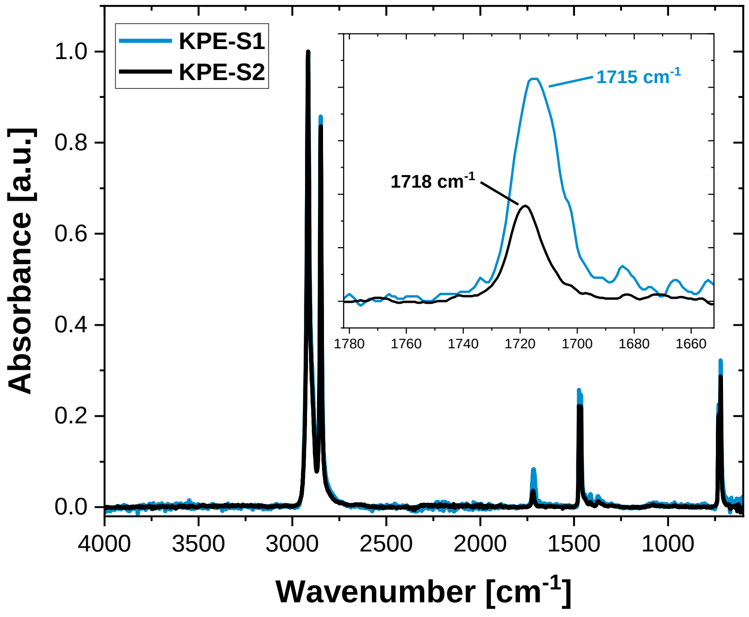

*Figure S18. IR spectra of keto-polyethylenes **KPE-S1** (light blue) and **KPE-S2** (black) used for tensile testing.*

3.2 Nuclear Magnetic Resonance Spectroscopy

3.2.1 Exemplary ^{1}H-NMR Spectrum of a Polyethylene

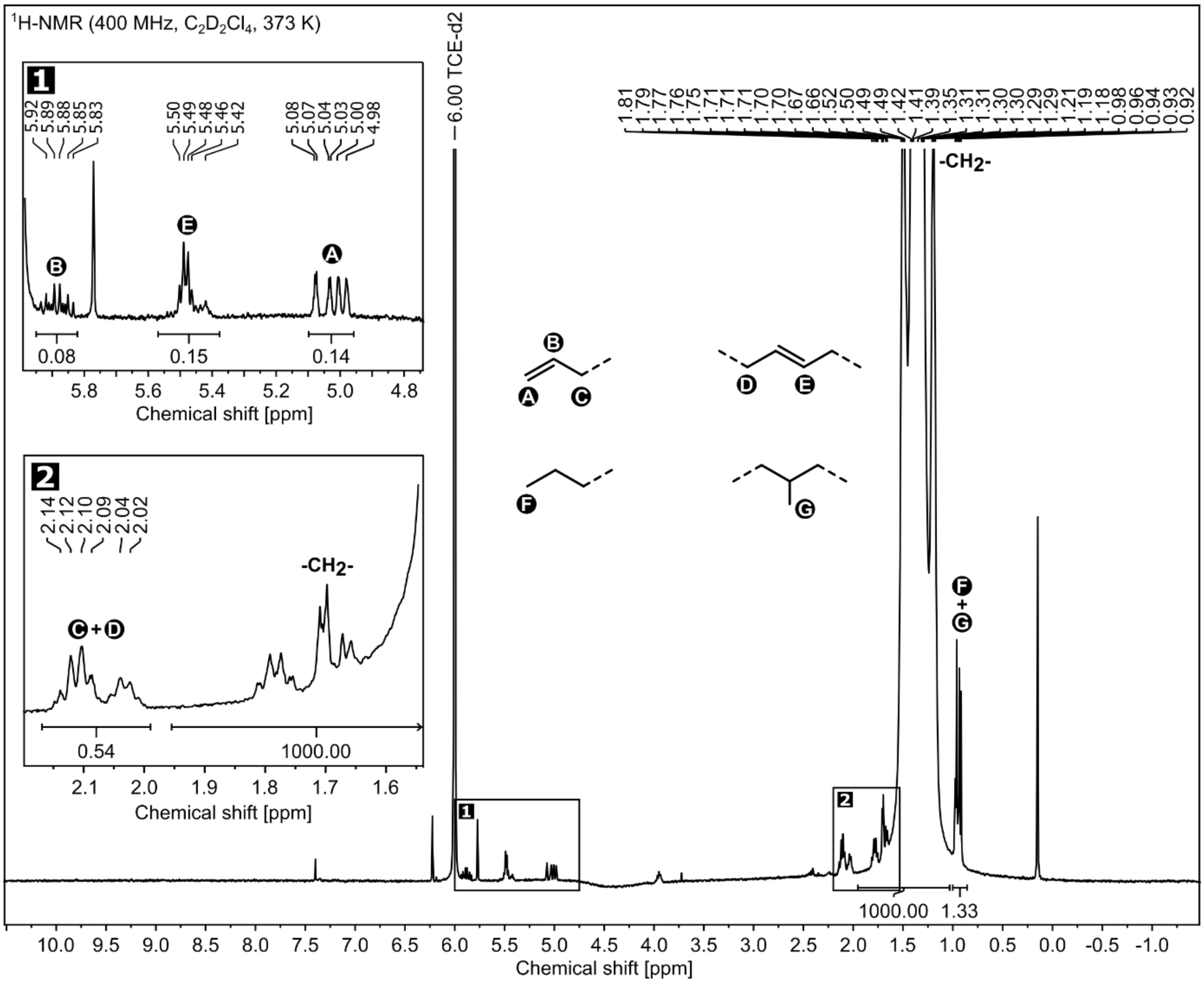

*Figure S19. ^{1}H-NMR spectrum of a polyethylene (**PE1**) with (400 MHz, $C_2D_2Cl_4$, 373 K).*

3.2.2 Exemplary ^{1}H-NMR Spectrum of a Keto-Polyethylene

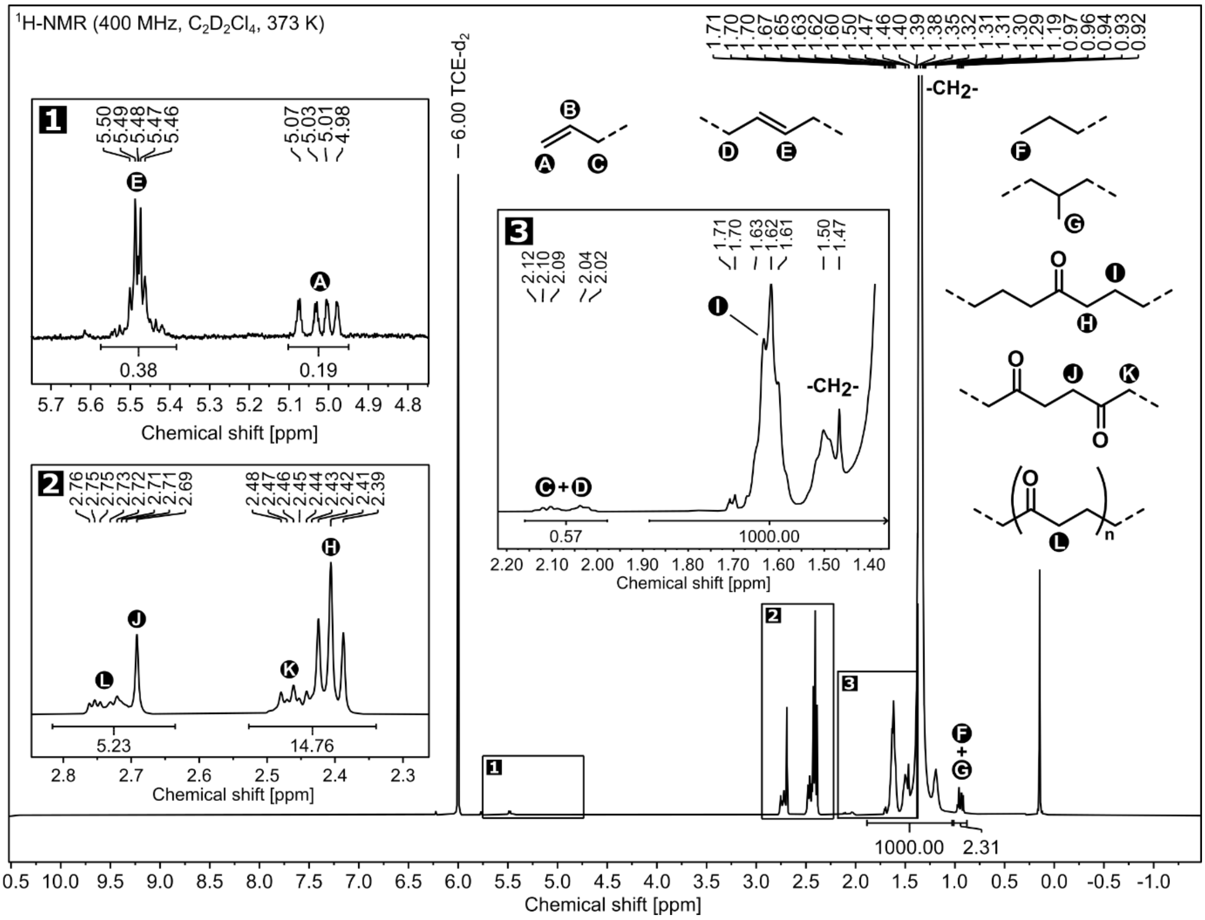

*Figure S20. ^{1}H-NMR spectrum of a keto-polyethylene (**KPE7**) with 2.0 mol-% of keto groups (IR: 2.6 mol-%) (400 MHz, $C_2D_2Cl_4$, 373 K).*

3.2.3 Calculation of Molecular Weight *via* ^{1}H-NMR Spectroscopy

To calculate the molecular weight of polyethylenes and keto-polyethylenes from ^{1}H-NMR spectra, the number of methine (n_{CH}), methylene (n_{CH2}), methyl (n_{CH3}) and keto groups ($n_{C=O}$) were multiplied with their respective masses and then summed up (see equation (S*1*) and divided by the number of end groups n_{EG}. To calculate the respective number of units n_x , the integrals of all relevant resonances were summed up and then divided by the number of protons contributing to those resonances (see equations (S*2* - (S8 and Figure S20).

$$M_n = \frac{0.013\,\frac{kg}{mol}\cdot n_{CH} + 0.014\,\frac{kg}{mol}\cdot n_{CH2} + 0.015\,\frac{kg}{mol}\cdot n_{CH3} + 0.028\,\frac{kg}{mol}\cdot n_{C=O}}{n_{EG}} \quad \text{(S1)}$$

$$n_{CH} = I_E + \frac{I_A}{2} \quad \text{(S2)}$$

$$n_{CH2} = \frac{I_{backbone} + I_{C+D} + I_A + I_J + I_K + I_L + I_H}{2} \quad \text{(S3)}$$

$$n_{CH3} = \frac{I_{F+G}}{3} \quad \text{(S4)}$$

$$n_{EG} = \frac{I_A + I_E}{2} + \frac{I_F}{3} \quad (S5)$$

$$n_{C=O} = \frac{I_H + I_J + I_K + I_L}{4} \quad (S6)$$

3.2.4 Microstructure Analysis *via* ^{1}H-NMR Spectroscopy

To calculate the overall keto group content of the keto-polyethylenes from ^{1}H-NMR spectra, the number of keto groups was divided through the sum of ethylene repeat units and keto groups (see equation (S7).

$$\Omega_{NMR}\,[\text{mol-\%}] = \frac{n_{C=O}}{\frac{(n_{CH} + n_{CH2} + n_{CH3})}{2} + n_{C=O}} \quad (S7)$$

To determine the keto group microstructure from the ^{1}H-NMR spectra, resonances between 2.85 ppm to 2.25 ppm, corresponding to the protons in the α-position to keto functional groups were analyzed (see Figure S21). The integral of each keto group type was divided by the total sum of all protons in the α-position to calculate the relative proportion of this type of keto groups (see equation (S8). Only the right half of resonance H was integrated to avoid integration errors due to overlap of resonances and multiplied by a factor of two to obtain the integral of the corresponding resonance. Subtraction of that value from the sum of integrals I_H and I_K yielded the integral of resonance K. The same procedure was applied to calculate the values of I_J and I_L.

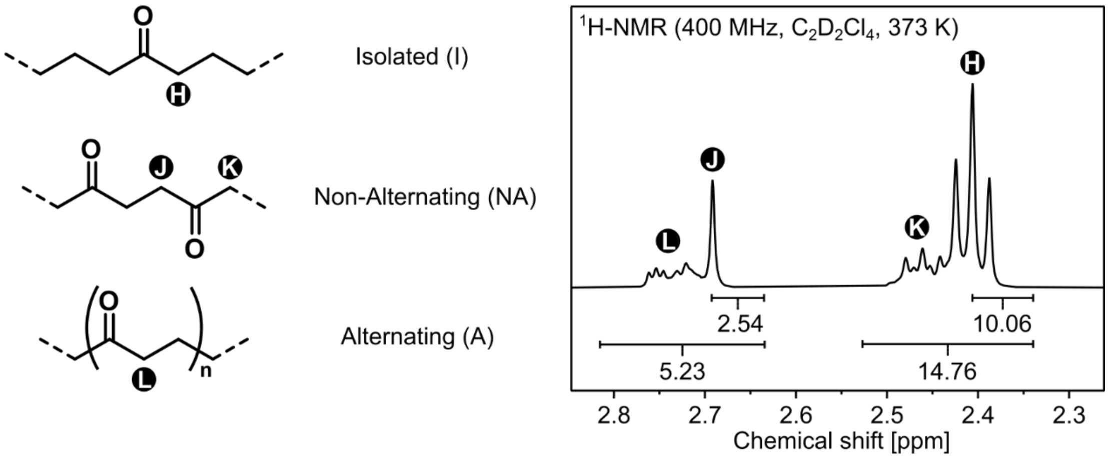

*Figure S21. Section of a ^{1}H-NMR spectrum of a keto-polyethylene (**KPE7**) with 2.0 mol-% of keto groups (IR: 2.6 mol-%) (400 MHz, $C_2D_2Cl_4$, 373 K).*

$$\text{I / NA / A}\,[\%] = \frac{I_H \text{ or } I_J + I_K \text{ or } I_L}{I_H + I_J + I_K + I_L} \quad (S8)$$

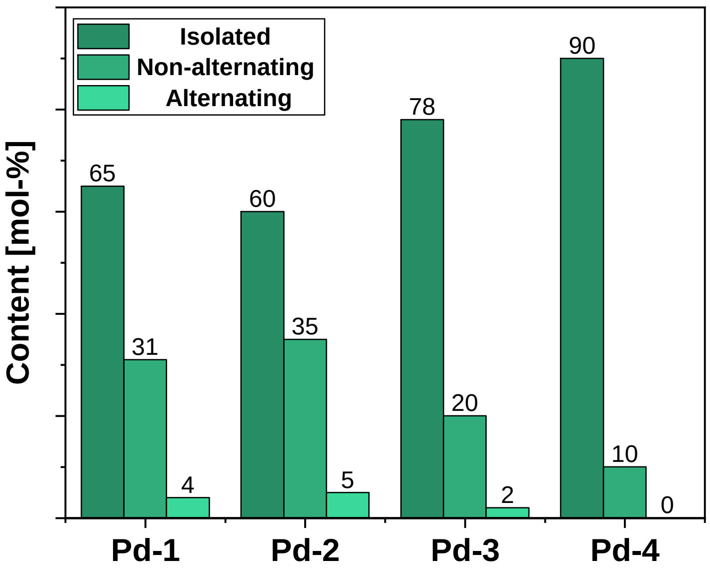

Figure S22. Microstructure of keto groups in the keto-PEs ***KPE1 – KPE4*** *determined via* ^{1}*H-NMR spectroscopy.*

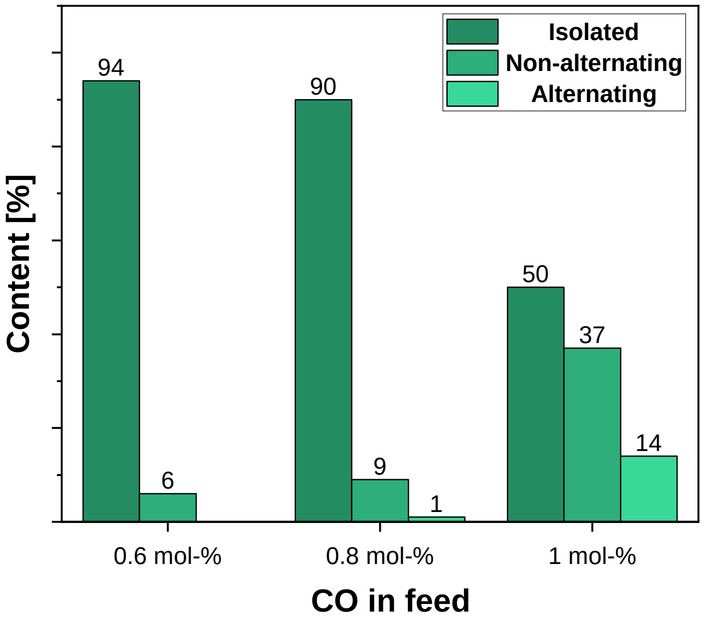

Figure S23. Microstructure of keto groups in the corresponding keto-PEs ***KPE5*** *(0.6 mol-%),* ***KPE6*** *(0.8 mol-%) and* ***KPE7*** *(1.0 mol-%) dependent on the carbon monoxide content in the monomer feed, determined via* ^{1}*H-NMR spectroscopy.*

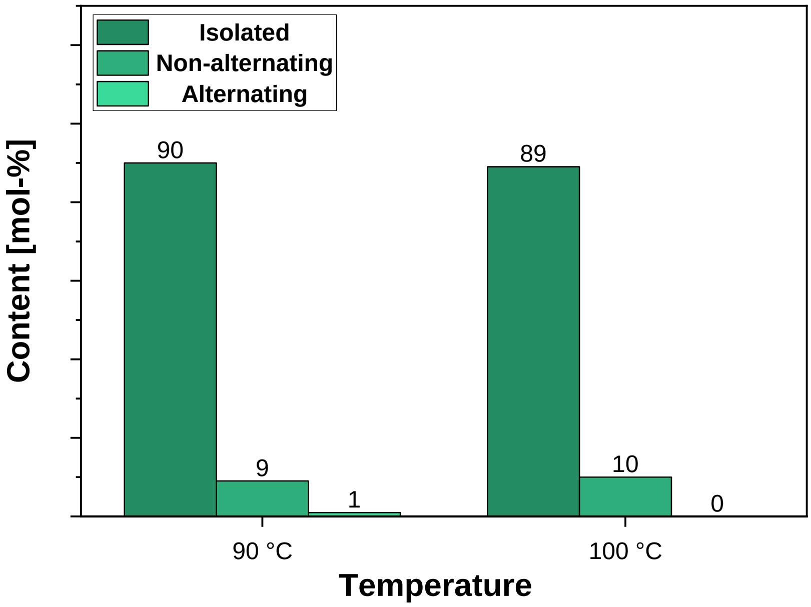

*Figure S24. Microstructure of keto groups in the corresponding keto-PEs **KPE6** (90 °C) and **KPE8** (100 °C) dependent on the polymerization temperature, determined via ^1H-NMR spectroscopy.*

3.3 Gel-Permeation Chromatography

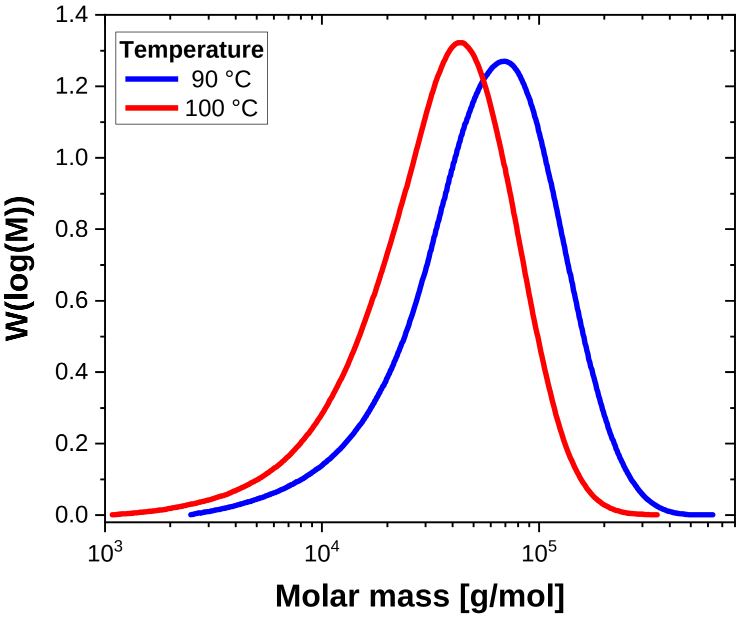

*Figure S25. GPC traces of two keto-polyethylenes obtained with **Pd-2** at 10 bar and with 0.8 mol-% of CO in the feed gas at 90 °C (blue, **KPE6**) and 100 °C (red, **KPE8**).*

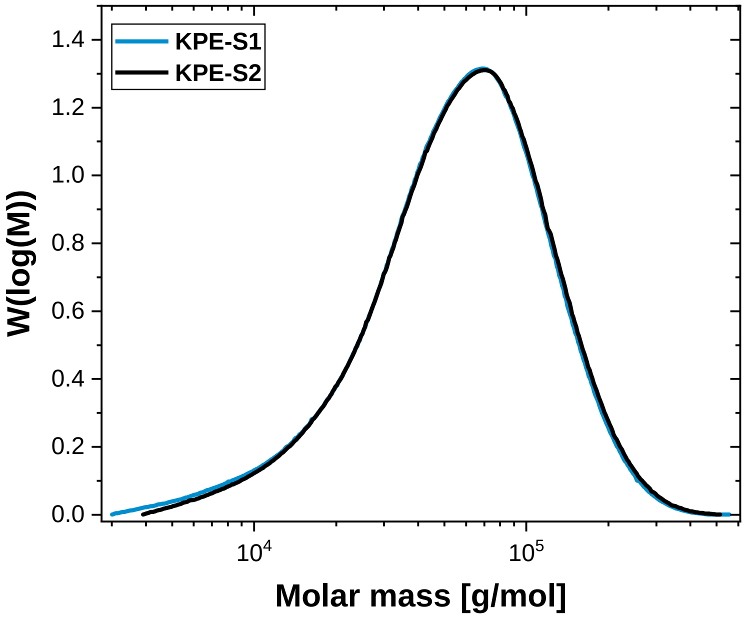

*Figure S26. GPC traces of keto-polyethylenes **KPE-S1** (light blue) and **KPE-S2** (black) used for tensile testing.*

3.4 Differential Scanning Calorimetry

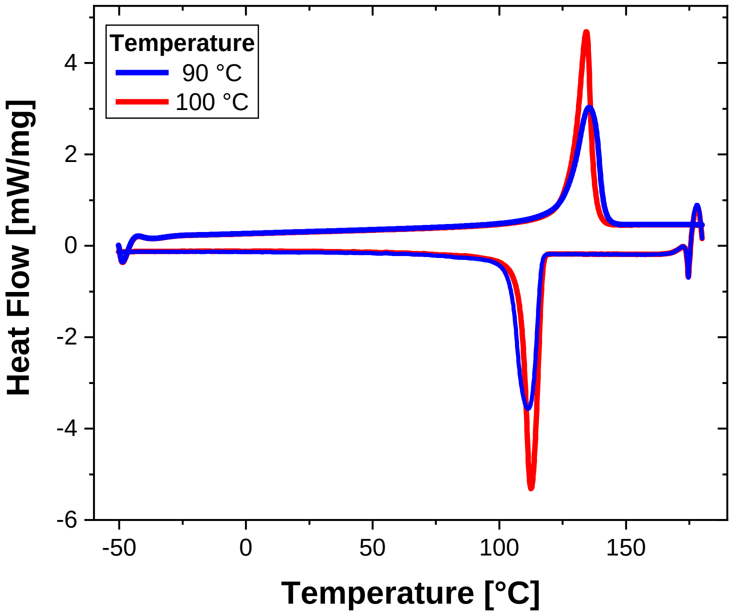

*Figure S27. DSC curves of two keto-polyethylenes obtained with **Pd-2** at 10 bar and with 0.8 mol-% of CO in the feed gas at 90 °C (blue, **KPE6**) and 100 °C (red, **KPE8**).*

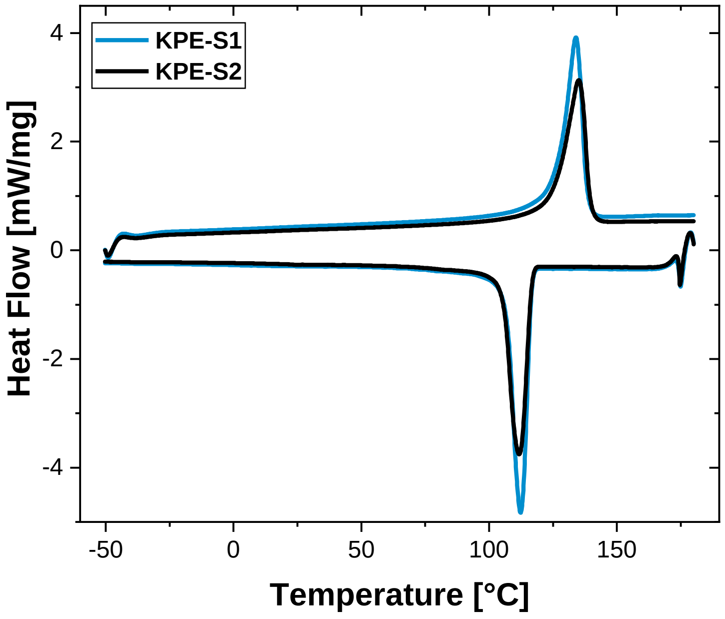

*Figure S28. DSC curves of keto-polyethylenes **KPE-S1** (light blue) and **KPE-S2** (black) used for tensile testing.*

4. Tensile Testing

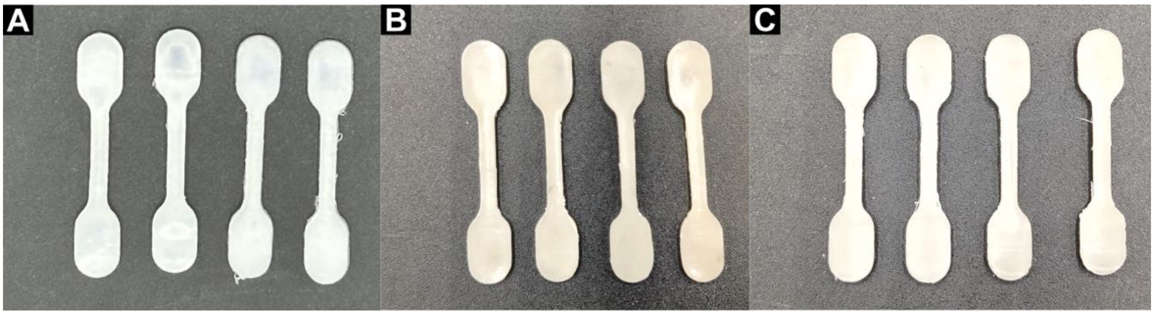

*Figure S29. Melt-pressed tensile-testing specimens of A) **PE1**, B) **KPE-S1** and C) **KPE-S2** according to DIN EN ISO 527-2, type 5B.*

*Table S2. Results of tensile testing of keto-polyethylenes with 0.5 mol-% and 1.4 mol-% of keto groups and a high-density polyethylene reference material obtained with catalyst **Pd-2**.*

Material	E [MPa]	σ_y [MPa]	ε_y [%]	ε_B [%]
HDPE	1032 ± 95	21.8 ± 0.9	8.0 ± 0.3	1170 ± 198
Keto-PE (Ω = 0.5 mol-%)	1263 ± 93	25.2 ± 0.5	6.6 ± 0.1	1069 ± 100
Keto-PE (Ω = 1.4 mol-%)	1058 ± 252	22.3 ± 1.7	6.1 ± 0.8	930 ± 196

5. Density Functional Theory

5.1 Computational Details

All DFT geometry optimizations were performed at the BP86[18–20] level with the Gaussian09 package.[21] The electronic configuration of the systems was described with the 6-31G(d) basis set for O, C, H, P, and S while for Pd the quasi-relativistic LANL2DZ ECP effective core potential was adopted.[22] All geometries were characterized as minimum or transition state through frequency calculations. The reported Gibbs energies were built through single point energy calculations on the BP86/6-31G(d) geometries using the M06 functional and the triple-ζ TZVP[23–25] basis set on main group atoms. Solvent effects (Toluene) were included with the PCM method.[26,27] To this M06/TZVP electronic energy in solvent, thermal corrections were included from the gas-phase frequency calculations at the BP86/6- 31G(d).

5.2 Alternative Alternating Pathway for **Pd-1** and **Pd-4**

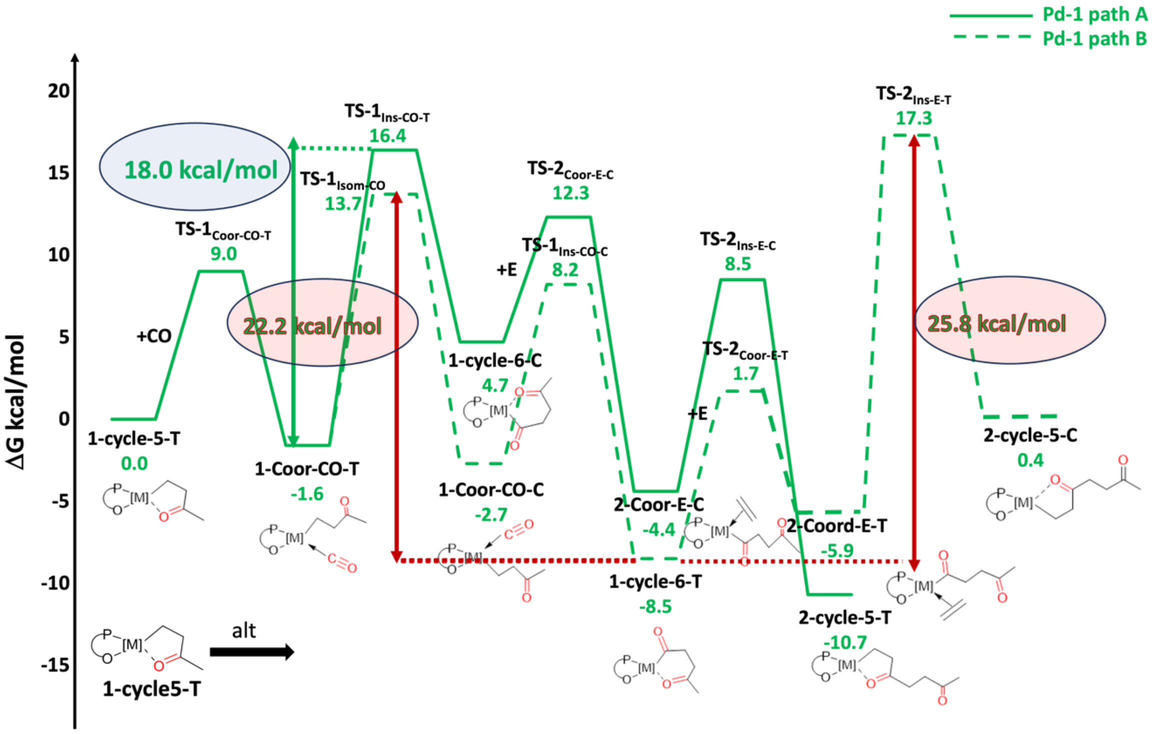

*Scheme S1. Gibbs energies in toluene ($\Delta G_{toluene}$ in kcal/mol) of key species for the two competitive alternating chain growth paths with **Pd-1** (green).*

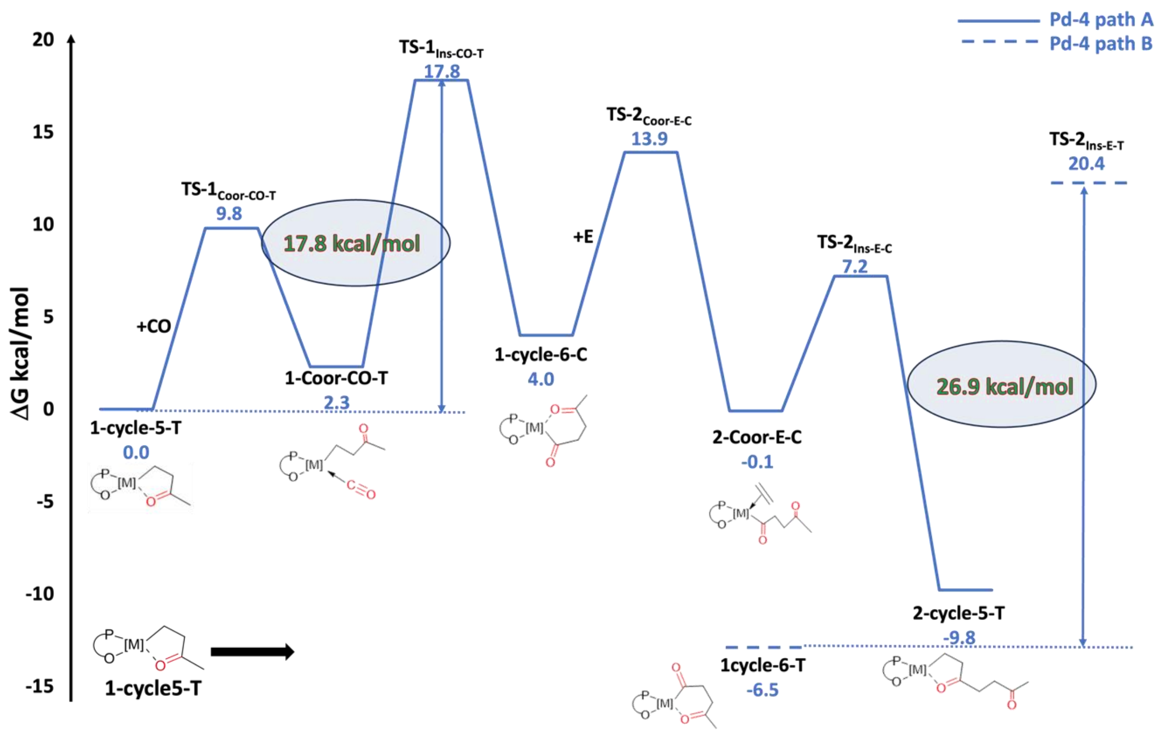

*Scheme S2. Gibbs energies in toluene ($\Delta G_{toluene}$ in kcal/mol) of key species for the two competitive alternating chain growth paths with **Pd-4** (blue).*

5.3 Steric analysis of **1-Coor-CO-T** for **Pd-4**

The higher energy of **1-Coor-CO-T** for **Pd-4** is mainly due to the steric requirements of the phosphorus ligand: when the chain is in a cis position relative to P, short distances are created between the chain's carbon atoms and the ligand's atoms, as shown in the Scheme S3. In fact, **1-Coor-E-T** also shows higher energy for **Pd-4** compared to the other systems.

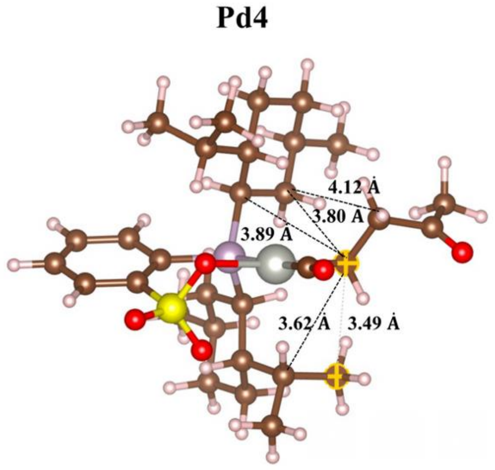

*Scheme S3. Geometry of **1-Coor-CO-T** intermediate for **Pd-4**.*

5.4 Cartesian Coordinates

Ethylene (E)

SCF done: -78.54471322 A.U.

C	0.000000	0.000000	0.670347
C	0.000000	0.000000	-0.670347
H	0.000000	0.931560	1.248984
H	0.000000	0.931560	-1.248984
H	0.000000	-0.931560	-1.248984
H	0.000000	-0.931560	1.248984

Carbon Monoxide (CO)

SCF done: -113.2945589 A.U.

C	0.000000	0.000000	-0.657221
O	0.000000	0.000000	0.492916

<u>Catalyst Pd-2</u>

Non-alternating pathway

1-cycle 5-T

SCF done: -2937.58871 A.U.

Pd	1.429996	1.343553	4.618187
P	-0.454356	2.274213	3.801695
S	0.036332	2.046154	7.342261
O	0.480990	0.838660	6.483888
O	-0.405679	1.572116	8.682895
O	1.008203	3.177619	7.310163
O	1.821934	5.143076	4.964575
O	-2.578090	6.241572	3.699744
O	0.948889	-1.444485	1.835534
O	-2.002194	-0.728641	5.435791
C	-1.493206	2.635548	6.510949
C	-2.535352	2.957191	7.401169
H	-2.353343	2.816110	8.470762
C	-3.774641	3.408210	6.931931
H	-4.576563	3.641231	7.641992
C	-3.981824	3.542932	5.552064
H	-4.949566	3.878126	5.161598
C	-2.947883	3.229761	4.661043
H	-3.130291	3.342276	3.590524
C	-1.686867	2.777089	5.112054
C	2.907100	5.174941	5.900965
H	3.766290	4.741352	5.364905
H	2.670246	4.563561	6.787184
H	3.152644	6.215661	6.191957
C	0.631566	5.687791	5.368361
C	0.426424	6.299973	6.622765
H	1.206857	6.284592	7.385336
C	-0.807900	6.898252	6.895667
H	-0.970510	7.365604	7.873664
C	-1.845476	6.910436	5.951021
H	-2.798088	7.387591	6.192202
C	-1.635841	6.286096	4.707435
C	-0.409302	5.643898	4.407756
C	-3.810027	6.921871	3.931646
H	-4.396646	6.800404	3.007486
H	-3.650792	8.001017	4.125717
H	-4.365294	6.478538	4.781471
C	-0.155148	5.099116	3.035810
C	0.146836	6.058814	2.039972
H	0.139979	7.114417	2.332929
C	0.460961	5.700526	0.725170
H	0.695470	6.474441	-0.014731
C	0.482663	4.343197	0.372148
H	0.736491	4.032953	-0.647904
C	0.170403	3.375738	1.334314
H	0.175504	2.320828	1.041460
C	-0.171986	3.725705	2.666846
C	1.992993	-2.193346	1.215965
H	2.012995	-1.864909	0.165173
H	1.794569	-3.282432	1.259222
H	2.978439	-1.988860	1.680364
C	0.662032	-1.735614	3.155685
C	1.421563	-2.621073	3.944079
H	2.304365	-3.124169	3.541829
C	1.028507	-2.850463	5.272189
H	1.621795	-3.529320	5.895390
C	-0.097923	-2.229796	5.821183
H	-0.367896	-2.410557	6.863320
C	-0.855760	-1.347547	5.023354
C	-0.474461	-1.072779	3.684967
C	-2.404476	-0.916237	6.800377
H	-3.332935	-0.336065	6.909319
H	-1.642884	-0.526667	7.498979
H	-2.611961	-1.984088	7.010928
C	-1.375089	-0.284804	2.781324
C	-2.205266	-1.054578	1.930766
H	-2.128672	-2.145625	1.992550
C	-3.104617	-0.468863	1.034826
H	-3.731967	-1.099673	0.394594
C	-3.187432	0.929104	0.962230
H	-3.875677	1.414412	0.260850
C	-2.372725	1.711142	1.788777
H	-2.418872	2.799445	1.684815
C	-1.464513	1.132699	2.711354
C	2.555489	1.626252	2.928929
H	2.417599	0.697845	2.342237
H	2.257628	2.490816	2.318698
C	4.016805	1.784365	3.404403
H	4.766665	1.627460	2.599206
H	4.180913	2.823938	3.762423
C	4.302088	0.902318	4.592045
O	3.353573	0.563610	5.332421
C	5.705278	0.443715	4.909275
H	6.404839	1.300059	4.897879
H	5.736951	-0.057719	5.888250
H	6.051481	-0.257327	4.125494

TS-1$_{\mathbf{Coor\text{-}E\text{-}T}}$

SCF done: -3016.136087 A.U.

Pd	1.794518	1.587307	4.970202
P	-0.167639	2.424728	4.097522
S	0.460932	2.931006	7.559894
O	1.044010	1.607769	7.009243

O	0.218666	2.819745	9.022427
O	1.220180	4.128870	7.092127
O	1.447998	6.488369	5.485959
O	-2.710193	5.453204	3.534309
O	0.808050	-1.573768	1.529226
O	-1.463946	-0.348513	5.476321
C	-1.193512	3.000474	6.781289
C	-2.241424	3.219309	7.694635
H	-1.975001	3.372295	8.744509
C	-3.578780	3.189991	7.281942
H	-4.381953	3.349458	8.010603
C	-3.872305	2.919941	5.938724
H	-4.911825	2.845489	5.597981
C	-2.831488	2.721025	5.020597
H	-3.090795	2.490801	3.985974
C	-1.470937	2.781757	5.403731
C	2.243709	6.975386	6.573476
H	3.288153	6.842958	6.252443
H	2.058222	6.380290	7.484199
H	2.052287	8.050491	6.762314
C	0.089207	6.488935	5.652209
C	-0.569707	7.090500	6.744743
H	-0.001610	7.520602	7.572193
C	-1.968494	7.129073	6.761101
H	-2.481457	7.595658	7.610026
C	-2.729775	6.593082	5.712361
H	-3.819916	6.653650	5.742236
C	-2.062802	5.975957	4.636671
C	-0.651070	5.874391	4.610612
C	-4.119413	5.662254	3.449357
H	-4.425896	5.241620	2.478908
H	-4.370522	6.740622	3.479316
H	-4.658582	5.138802	4.262715
C	0.047085	5.326689	3.403973
C	0.546076	6.301298	2.503996
H	0.425514	7.354329	2.780385
C	1.157700	5.964697	1.293661
H	1.526847	6.750889	0.625182
C	1.271012	4.611155	0.942064
H	1.719275	4.314727	-0.013330
C	0.796935	3.630892	1.819358
H	0.872845	2.582113	1.518350
C	0.198116	3.953757	3.066441
C	1.638621	-2.474194	0.799144
H	1.602183	-2.130565	-0.246136
H	1.265433	-3.515512	0.858708
H	2.688644	-2.448145	1.153993
C	0.626391	-1.818959	2.876481
C	1.303609	-2.838962	3.573396
H	1.996819	-3.509868	3.059772
C	1.065285	-2.994709	4.947196
H	1.592190	-3.782095	5.498056
C	0.171695	-2.164170	5.629690
H	0.012222	-2.298289	6.701242
C	-0.516099	-1.156433	4.919310
C	-0.288861	-0.952819	3.533106
C	-1.689667	-0.464935	6.890395
H	-2.458259	0.286043	7.122239
H	-0.768015	-0.235261	7.453056
H	-2.065501	-1.474174	7.148745
C	-1.112502	0.004411	2.725106
C	-1.941947	-0.598210	1.744331
H	-1.927875	-1.689992	1.664384
C	-2.763295	0.145387	0.893668
H	-3.390595	-0.364862	0.153597
C	-2.772819	1.544404	0.997539
H	-3.404183	2.151737	0.338858
C	-1.965347	2.166004	1.956425
H	-1.978141	3.258375	2.032638
C	-1.134996	1.424814	2.838389
C	2.618512	1.197815	3.126944
H	1.916214	0.557303	2.566485
H	2.786840	2.131128	2.566265
C	3.954485	0.460816	3.352376
H	4.316070	-0.032546	2.424956
H	4.749823	1.188232	3.625177
C	3.913380	-0.520005	4.506433
O	3.145843	-0.317399	5.460554
C	4.860760	-1.702998	4.511499
H	5.895908	-1.375748	4.298776
H	4.821623	-2.223047	5.480483
H	4.575259	-2.406470	3.706853
C	4.050109	2.685269	6.110829
H	4.918417	2.038123	5.936906
H	3.609102	2.664792	7.112020
C	3.550272	3.500061	5.146015
H	2.731124	4.193331	5.362120
H	4.013593	3.565317	4.153070

1-Coor-E-T

SCF done: -3016.153470 A.U.

Pd	1.456274	1.120778	4.687171
P	-0.462371	2.189465	3.814293
S	-0.122317	1.826760	7.367877
O	0.294575	0.633507	6.476521
O	-0.638465	1.324103	8.670644
O	0.922364	2.893705	7.421317
O	1.860363	4.901988	5.132718
O	-2.429224	6.265101	3.746152
O	0.888208	-1.421911	1.762363
O	-2.114793	-0.897197	5.347276
C	-1.577466	2.532122	6.504013
C	-2.628892	2.890093	7.368981
H	-2.493034	2.710805	8.439623
C	-3.822657	3.425815	6.872327
H	-4.633891	3.686262	7.561741
C	-3.971368	3.610737	5.491110
H	-4.902211	4.015581	5.077896
C	-2.926232	3.261920	4.626956
H	-3.061631	3.424940	3.556218
C	-1.710537	2.720211	5.103416
C	2.927995	4.902044	6.090213
H	3.772176	4.409793	5.584036
H	2.643139	4.329846	6.988542
H	3.218219	5.936318	6.360887
C	0.690820	5.510583	5.503120

C	0.479672	6.115776	6.759871
H	1.236774	6.053665	7.543235
C	-0.729115	6.775204	7.005071
H	-0.896309	7.238038	7.984365
C	-1.734251	6.855435	6.029639
H	-2.667868	7.378491	6.248857
C	-1.517918	6.241582	4.782074
C	-0.317368	5.540195	4.507812
C	-3.620906	7.024558	3.944655
H	-4.177765	6.958885	2.996844
H	-3.395790	8.086369	4.165680
H	-4.238556	6.606941	4.764070
C	-0.052020	5.017852	3.128657
C	0.296378	5.992531	2.162705
H	0.331118	7.038469	2.486420
C	0.594396	5.662091	0.836706
H	0.858802	6.447887	0.119944
C	0.553119	4.316828	0.441631
H	0.782772	4.029054	-0.590574
C	0.205290	3.333527	1.375352
H	0.158959	2.290750	1.046333
C	-0.120459	3.655933	2.719313
C	1.997100	-2.072521	1.139325
H	2.058227	-1.648935	0.125308
H	1.841722	-3.167126	1.071692
H	2.943653	-1.870203	1.677229
C	0.575705	-1.797508	3.049729
C	1.288110	-2.769511	3.780476
H	2.157769	-3.274862	3.353659
C	0.855046	-3.093317	5.076202
H	1.405177	-3.849036	5.648992
C	-0.258809	-2.474554	5.656053
H	-0.563579	-2.736759	6.671236
C	-0.974962	-1.510568	4.914382
C	-0.557060	-1.146914	3.607710
C	-2.532598	-1.144075	6.698229
H	-3.447150	-0.547122	6.830593
H	-1.766084	-0.806354	7.417613
H	-2.768692	-2.215085	6.853967
C	-1.444316	-0.312041	2.731769
C	-2.299918	-1.044547	1.872996
H	-2.254464	-2.138252	1.915296
C	-3.183976	-0.418061	0.989899
H	-3.832271	-1.019554	0.342544
C	-3.222218	0.982848	0.936497
H	-3.895597	1.498925	0.242787
C	-2.384249	1.727452	1.773669
H	-2.396114	2.817852	1.684581
C	-1.494652	1.107317	2.687440
C	2.591726	1.364759	2.970522
H	3.304156	0.520459	3.014459
H	1.922895	1.203379	2.107365
C	3.360015	2.693649	2.859498
H	2.675329	3.526865	2.627406
H	3.878537	2.925178	3.805168
C	4.457601	2.554518	1.787729
O	5.543780	2.045885	2.056259
C	4.122940	3.041034	0.382270
H	3.963185	4.135883	0.392724
H	4.937927	2.790898	-0.314655
H	3.173301	2.593146	0.035060
C	3.352906	1.010198	5.944510
H	4.169968	1.505541	5.408065
H	2.966997	1.507084	6.842546
C	2.980879	-0.281843	5.622155
H	2.294606	-0.849752	6.259617
H	3.484543	-0.835979	4.822702

TS-1$_{Isom-E}$

SCF done: -3016.124231 A.U.

Pd	1.111980	-1.077508	-0.942375
P	-0.392972	0.910658	-0.263534
S	-0.157924	-1.696724	2.084891
O	-0.061389	-2.144482	0.604317
O	-0.713148	-2.802673	2.910448
O	1.116660	-1.072823	2.568040
O	3.499367	2.723541	-1.408010
O	1.166261	2.261665	2.653504
O	-2.021208	-2.703285	-1.539866
O	-4.646905	1.061302	-0.502550
C	-1.393602	-0.341321	2.149822
C	-2.209794	-0.387552	3.295121
H	-2.134344	-1.265132	3.943994
C	-3.082196	0.665358	3.598806
H	-3.700860	0.619019	4.502521
C	-3.142365	1.778112	2.747773
H	-3.795726	2.626398	2.983756
C	-2.355537	1.811385	1.587969
H	-2.412573	2.688671	0.939333
C	-1.474909	0.758061	1.253872
C	4.709089	2.672586	-2.164924
H	4.477702	3.153524	-3.127780
H	5.037053	1.630053	-2.348591
H	5.527806	3.225048	-1.663389
C	3.541214	2.249438	-0.114107
C	4.666032	1.615266	0.450434
H	5.580710	1.465359	-0.128164
C	4.596935	1.172157	1.780743
H	5.467973	0.675836	2.224113
C	3.442059	1.344881	2.553113
H	3.408447	0.977127	3.580479
C	2.322535	1.986763	1.980746
C	2.354337	2.440592	0.637088
C	1.055215	1.803736	4.009859
H	0.042437	2.094440	4.326764
H	1.803707	2.300469	4.658533
H	1.160266	0.706131	4.058136
C	1.228942	3.259964	0.083274
C	1.449274	4.654134	-0.017865
H	2.420649	5.048166	0.300475
C	0.467420	5.524564	-0.502915
H	0.670851	6.599832	-0.565222
C	-0.772566	5.009219	-0.911784
H	-1.552844	5.673894	-1.300130
C	-1.007348	3.631425	-0.830010
H	-1.962683	3.231822	-1.187884

C	-0.025925	2.739257	-0.330438
C	-1.610390	-4.054938	-1.302524
H	-0.815116	-4.252487	-2.037529
H	-2.446006	-4.763217	-1.468878
H	-1.206031	-4.164605	-0.281176
C	-2.991701	-2.193030	-0.715596
C	-3.684707	-2.948570	0.253007
H	-3.421884	-3.990121	0.447534
C	-4.717103	-2.341246	0.978280
H	-5.253871	-2.926454	1.733648
C	-5.083022	-1.005454	0.760757
H	-5.898463	-0.561986	1.336757
C	-4.371384	-0.254048	-0.194937
C	-3.300713	-0.825886	-0.921022
C	-5.783950	1.650599	0.126874
H	-5.865287	2.665028	-0.293852
H	-5.652108	1.716943	1.224446
H	-6.710071	1.084936	-0.095634
C	-2.606601	-0.053000	-1.998938
C	-3.168853	-0.144961	-3.294383
H	-4.077586	-0.745487	-3.414051
C	-2.601932	0.494024	-4.401763
H	-3.065284	0.398663	-5.390334
C	-1.432416	1.250899	-4.228439
H	-0.964529	1.760489	-5.078770
C	-0.862722	1.361207	-2.955316
H	0.043173	1.965160	-2.832716
C	-1.436544	0.733079	-1.815756
C	2.865219	-1.612299	0.033602
H	3.698934	-1.411544	-0.656114
H	2.759007	-0.849595	0.820158
C	2.870852	-3.024607	0.588272
H	3.167030	-2.985444	1.658597
H	1.840706	-3.434385	0.640792
C	3.757868	-4.033499	-0.135494
O	4.272510	-3.813512	-1.232697
C	3.983838	-5.355606	0.591970
H	4.623216	-5.189321	1.480238
H	3.030252	-5.774016	0.963122
H	4.479611	-6.075362	-0.077316
C	2.118281	-0.611607	-2.751157
H	3.095789	-0.156281	-2.549259
H	1.432671	-0.005329	-3.356237
C	1.958219	-2.020783	-2.680138
H	1.108648	-2.500436	-3.185120
H	2.805467	-2.670355	-2.429969

1-Coor-E-C

SCF done: -3016.143823 A.U.

Pd	1.694690	1.090489	4.534507
P	-0.432292	2.222144	3.798494
S	0.530657	2.248354	7.122822
O	0.783320	0.843383	6.492231
O	0.469889	2.143101	8.603734
O	1.461078	3.264002	6.536611
O	1.830010	5.731404	4.974920
O	-2.699580	5.712725	3.709262
O	0.441975	-1.599904	1.365008
O	-1.899932	-0.576753	5.323024
C	-1.158491	2.665414	6.566684
C	-2.069826	2.935827	7.601868
H	-1.696235	2.925957	8.630009
C	-3.420851	3.175464	7.318391
H	-4.124048	3.375278	8.135011
C	-3.863732	3.128348	5.989202
H	-4.923599	3.275273	5.750517
C	-2.950901	2.876049	4.954569
H	-3.321185	2.826496	3.928404
C	-1.578625	2.661894	5.210270
C	2.873581	5.952504	5.930567
H	3.803074	5.654651	5.421991
H	2.721762	5.321506	6.823163
H	2.940092	7.021482	6.215405
C	0.547867	5.988530	5.374204
C	0.205249	6.592770	6.602373
H	0.971108	6.823314	7.345801
C	-1.136509	6.896398	6.861487
H	-1.403931	7.365264	7.815521
C	-2.146782	6.622266	5.927823
H	-3.183534	6.882149	6.152966
C	-1.796321	6.003766	4.711746
C	-0.456844	5.644453	4.434858
C	-4.035357	6.183988	3.883798
H	-4.568149	5.923113	2.955978
H	-4.062185	7.281891	4.027411
H	-4.531790	5.692599	4.743278
C	-0.068506	5.090772	3.098574
C	0.351459	6.043442	2.138999
H	0.350444	7.097865	2.436284
C	0.760377	5.681723	0.851824
H	1.080178	6.450060	0.138796
C	0.755988	4.326444	0.490347
H	1.069104	4.011688	-0.511666
C	0.340247	3.367582	1.420941
H	0.316322	2.316706	1.112523
C	-0.083911	3.718414	2.730655
C	1.324515	-2.442592	0.621592
H	1.238227	-2.112633	-0.424980
H	1.030427	-3.507332	0.699243
H	2.375509	-2.332431	0.953929
C	0.306561	-1.855484	2.714017
C	1.068436	-2.817682	3.404683
H	1.824500	-3.416838	2.891518
C	0.846108	-2.999546	4.778289
H	1.439957	-3.743451	5.321574
C	-0.118919	-2.257468	5.468485
H	-0.270784	-2.418233	6.537513
C	-0.884715	-1.301643	4.766557
C	-0.666338	-1.066292	3.383589
C	-2.151532	-0.754683	6.723968
H	-2.973078	-0.062932	6.960548
H	-1.260391	-0.490212	7.319594
H	-2.466004	-1.794334	6.941365
C	-1.563592	-0.157856	2.596911
C	-2.469736	-0.791573	1.712098
H	-2.463976	-1.885807	1.665424

C	-3.355539	-0.065558	0.909849
H	-4.043802	-0.591980	0.238613
C	-3.348993	1.336561	0.967626
H	-4.028610	1.924953	0.340608
C	-2.459480	1.986160	1.831610
H	-2.440573	3.081323	1.852238
C	-1.565361	1.263211	2.661686
C	3.309179	0.092806	5.390974
H	2.753458	-0.686896	5.941816
H	3.945374	-0.374841	4.619042
C	4.119713	1.012467	6.308890
H	4.791364	1.662055	5.720595
H	3.445834	1.650343	6.907487
C	4.997636	0.148711	7.236597
O	6.142219	-0.170224	6.927134
C	4.348720	-0.291776	8.543614
H	3.298966	-0.598441	8.387958
H	4.928761	-1.104085	9.008891
H	4.318105	0.571656	9.236569
C	2.499792	0.679734	2.558214
H	3.050789	-0.265025	2.625579
H	1.662207	0.682037	1.853984
C	3.059316	1.869472	3.043997
H	2.660689	2.844941	2.741701
H	4.061369	1.874507	3.487374

TS-1$_{Ins-E-C}$

SCF done: -3016.125898 A.U.

Pd	1.362174	-0.148831	-0.377268
P	-0.900118	0.163716	-0.866076
S	0.169243	0.439264	2.442307
O	0.885843	-0.716878	1.692139
O	0.240978	0.223836	3.912885
O	0.596070	1.778301	1.928100
O	-0.082955	4.215452	0.453634
O	-4.334840	2.495655	-0.580922
O	1.223009	-2.833887	-3.608003
O	-1.061074	-3.096577	0.503365
C	-1.574490	0.188708	1.964788
C	-2.462931	0.041806	3.043529
H	-2.060815	0.146090	4.055585
C	-3.810477	-0.271049	2.823017
H	-4.490168	-0.396440	3.673654
C	-4.267209	-0.449986	1.510274
H	-5.308336	-0.733171	1.316320
C	-3.385851	-0.283707	0.431819
H	-3.762864	-0.446901	-0.579730
C	-2.028880	0.058639	0.625328
C	0.859070	4.792322	1.364116
H	1.790217	4.904574	0.787922
H	1.031316	4.118821	2.221245
H	0.524000	5.789299	1.713842
C	-1.330176	3.931843	0.936069
C	-1.786343	4.293957	2.221323
H	-1.114580	4.766218	2.941053
C	-3.117625	4.037159	2.569245
H	-3.472612	4.316745	3.567941
C	-4.011586	3.438299	1.669260
H	-5.048940	3.264433	1.964191
C	-3.541740	3.064576	0.395329
C	-2.191202	3.267540	0.026771
C	-5.735231	2.419436	-0.315364
H	-6.193199	2.026175	-1.236490
H	-6.159475	3.417548	-0.090658
H	-5.955461	1.733231	0.525719
C	-1.725125	2.975248	-1.366407
C	-1.798499	4.059738	-2.274076
H	-2.189154	5.011225	-1.896944
C	-1.393707	3.950406	-3.607376
H	-1.465484	4.814558	-4.277638
C	-0.897356	2.723134	-4.071152
H	-0.576219	2.605111	-5.112387
C	-0.817580	1.636555	-3.193726
H	-0.444420	0.681519	-3.576744
C	-1.226353	1.731396	-1.837324
C	2.309673	-3.204820	-4.458590
H	2.029162	-2.858889	-5.465176
H	2.458310	-4.302035	-4.472362
H	3.255897	-2.714501	-4.155987
C	1.284903	-3.228682	-2.286655
C	2.408406	-3.855223	-1.712206
H	3.301438	-4.069760	-2.304132
C	2.365376	-4.210755	-0.355040
H	3.236291	-4.697816	0.098124
C	1.234104	-3.962708	0.431230
H	1.227516	-4.249429	1.484711
C	0.111943	-3.337319	-0.153823
C	0.126870	-2.944377	-1.517326
C	-1.113748	-3.413382	1.901945
H	-2.110289	-3.088645	2.235019
H	-0.337554	-2.859409	2.458018
H	-1.003871	-4.503368	2.066500
C	-1.104114	-2.411154	-2.187293
C	-1.762285	-3.299781	-3.071945
H	-1.342561	-4.303467	-3.199268
C	-2.914633	-2.933218	-3.774149
H	-3.397270	-3.650576	-4.447777
C	-3.438909	-1.641668	-3.613432
H	-4.335417	-1.329577	-4.161143
C	-2.803015	-0.743185	-2.748488
H	-3.202409	0.271678	-2.647043
C	-1.643398	-1.106207	-2.017742
C	3.504875	-0.459077	0.417544
H	2.964310	-1.233275	0.989364
H	4.426103	-0.912909	0.030483
C	3.815794	0.794960	1.228058
H	4.385396	1.518031	0.613014
H	2.896382	1.282251	1.594312
C	4.730072	0.377470	2.407742
O	5.897078	0.048461	2.210559
C	4.089426	0.379424	3.784510
H	3.847533	1.420088	4.075779
H	3.122669	-0.157421	3.770327
H	4.770980	-0.060802	4.529011
C	3.290382	-0.034554	-1.685268
H	3.703300	-1.010775	-1.962072

H	4.036638	0.748735	-1.509687
C	2.037310	0.339551	-2.279539
H	1.828408	1.402801	-2.457192
H	1.587870	-0.356001	-3.000674

1-B-T

SCF done: -3016.161322 A.U.

Pd	0.935542	-0.479603	0.246312
P	-1.021762	-0.039287	-0.739342
S	-0.724216	-0.523453	2.819717
O	0.101670	-1.502734	1.941909
O	-1.115081	-1.191911	4.089146
O	-0.083349	0.821551	2.924689
O	0.242351	3.190528	0.778139
O	-4.187605	3.181905	-0.816617
O	1.562356	-2.670213	-2.957917
O	-1.459551	-3.705209	0.498040
C	-2.294166	-0.294291	1.881028
C	-3.442896	-0.382342	2.690044
H	-3.301050	-0.598389	3.753083
C	-4.724701	-0.227129	2.149178
H	-5.604726	-0.311308	2.796992
C	-4.868914	0.024712	0.778247
H	-5.863051	0.138291	0.331158
C	-3.731370	0.115335	-0.033472
H	-3.865557	0.317475	-1.097911
C	-2.426971	-0.036606	0.490165
C	1.211064	3.440200	1.806789
H	2.189683	3.295225	1.323557
H	1.091150	2.722070	2.634672
H	1.139526	4.482420	2.175411
C	-1.077002	3.356951	1.105248
C	-1.532294	3.767511	2.375617
H	-0.834050	3.894473	3.204560
C	-2.900707	3.981024	2.572486
H	-3.255814	4.291284	3.561827
C	-3.830748	3.804848	1.536616
H	-4.892938	3.981352	1.720926
C	-3.368323	3.385146	0.275549
C	-1.993967	3.130382	0.047972
C	-5.570146	3.498451	-0.660077
H	-6.030282	3.321065	-1.644749
H	-5.715848	4.558050	-0.371866
H	-6.054477	2.848284	0.094881
C	-1.493168	2.798787	-1.323147
C	-1.410168	3.874589	-2.238578
H	-1.762413	4.856575	-1.904445
C	-0.878235	3.721960	-3.523190
H	-0.821892	4.582182	-4.199942
C	-0.401957	2.464695	-3.925571
H	0.036952	2.324725	-4.919979
C	-0.495148	1.378465	-3.048557
H	-0.132990	0.398346	-3.377007
C	-1.062514	1.514417	-1.755017
C	2.790507	-2.919848	-3.640150
H	2.665625	-2.490308	-4.646328
H	2.996507	-4.004789	-3.723375
H	3.649452	-2.428150	-3.140128
C	1.407018	-3.237525	-1.708198
C	2.449728	-3.862258	-0.995334
H	3.460268	-3.923054	-1.407048
C	2.166289	-4.426184	0.259786
H	2.972599	-4.911284	0.821914
C	0.880454	-4.384151	0.809560
H	0.692237	-4.817013	1.793954
C	-0.159040	-3.758903	0.087980
C	0.097575	-3.155410	-1.169854
C	-1.764774	-4.193553	1.813024
H	-2.845105	-4.027655	1.938447
H	-1.211583	-3.626323	2.581550
H	-1.548585	-5.276789	1.896850
C	-1.024064	-2.638824	-2.021465
C	-1.551088	-3.554056	-2.963394
H	-1.135065	-4.567293	-2.982692
C	-2.566521	-3.199253	-3.857246
H	-2.951038	-3.936045	-4.571718
C	-3.070967	-1.890471	-3.841306
H	-3.849275	-1.582032	-4.548446
C	-2.565589	-0.968073	-2.918256
H	-2.935269	0.061464	-2.949302
C	-1.558499	-1.322688	-1.985819
C	4.686503	-0.097330	-0.751407
H	5.419209	-0.273115	0.059563
H	4.530588	-1.067495	-1.261553
C	5.247158	0.950681	-1.740373
H	4.537929	1.053861	-2.587968
H	5.328376	1.938984	-1.254213
C	6.630726	0.610788	-2.306796
O	7.568390	1.398104	-2.219497
C	6.793604	-0.749298	-2.985036
H	6.787858	-1.553859	-2.224654
H	5.959764	-0.958385	-3.680845
H	7.752764	-0.781697	-3.524275
C	3.341247	0.361475	-0.135168
H	3.474276	1.312403	0.412283
H	3.112550	-0.399565	0.681642
C	2.159679	0.445832	-1.091802
H	1.815839	1.460213	-1.338768
H	2.254410	-0.184396	-1.994483

Alternating pathway, path A

TS-1$_{\text{Ins-CO-T}}$

SCF done: -3050.876768 A.U.

P	0.223873	-0.367747	0.478390
S	1.395523	-0.318314	-2.695409
O	-0.076460	-0.847674	-2.791749
O	2.128660	-0.581976	-3.957842
O	1.424557	1.070469	-2.137862
O	3.067186	3.018258	-0.822570
O	3.559555	-0.219508	2.561069
O	-4.079140	-0.655372	1.782050
O	-1.051585	-3.431102	-0.521512
C	2.105271	-1.414264	-1.425973
C	3.100935	-2.299683	-1.868091
H	3.425515	-2.228796	-2.910605

C	3.634185	-3.258099	-0.995489
H	4.405889	-3.951500	-1.348918
C	3.153381	-3.335675	0.319456
H	3.534883	-4.101273	1.005271
C	2.166693	-2.441732	0.763308
H	1.790115	-2.534715	1.784511
C	1.638422	-1.444409	-0.085142
C	3.383237	3.542181	-2.116364
H	2.632674	4.324295	-2.306457
H	3.296053	2.756081	-2.886586
H	4.394656	3.995306	-2.136357
C	3.797432	1.952619	-0.378020
C	4.999311	1.515889	-0.974635
H	5.369746	1.982966	-1.889787
C	5.723038	0.479830	-0.372304
H	6.658861	0.142163	-0.832354
C	5.284820	-0.129080	0.812392
H	5.877630	-0.924738	1.268836
C	4.070574	0.296270	1.385472
C	3.290532	1.309472	0.781195
C	4.382453	-1.138184	3.281124
H	3.834761	-1.366079	4.208759
H	5.362822	-0.689280	3.533243
H	4.544926	-2.074420	2.712402
C	2.036638	1.814910	1.424786
C	2.149766	3.033022	2.138657
H	3.132116	3.516817	2.165931
C	1.065769	3.617802	2.800163
H	1.198414	4.557100	3.349189
C	-0.184814	2.981088	2.762298
H	-1.046926	3.404217	3.291025
C	-0.323571	1.781094	2.055327
H	-1.299479	1.279798	2.057875
C	0.769228	1.176663	1.380465
C	-5.337091	0.021470	1.904328
H	-5.343277	0.441239	2.921604
H	-6.186207	-0.679259	1.790370
H	-5.424524	0.849633	1.176482
C	-3.847453	-1.385174	0.638329
C	-4.747473	-1.458311	-0.444021
H	-5.708896	-0.939505	-0.413661
C	-4.384323	-2.200526	-1.578208
H	-5.077907	-2.256654	-2.424920
C	-3.152934	-2.862872	-1.659454
H	-2.891580	-3.422793	-2.559891
C	-2.260275	-2.793458	-0.566630
C	-2.593943	-2.052457	0.596784
C	-0.576666	-4.040022	-1.733208
H	0.434424	-4.402009	-1.495427
H	-0.522160	-3.294973	-2.546811
H	-1.215641	-4.895316	-2.027730
C	-1.712258	-2.035576	1.808278
C	-2.172324	-2.740973	2.945645
H	-3.117626	-3.288852	2.868147
C	-1.460431	-2.743765	4.149911
H	-1.845489	-3.302077	5.010995
C	-0.263574	-2.016700	4.250872
H	0.296742	-1.994470	5.192646
C	0.210640	-1.309663	3.138999
H	1.136818	-0.729375	3.220600
C	-0.488490	-1.321386	1.906706
Pd	-1.293154	0.248395	-1.400350
C	-2.568795	1.101662	-2.461862
O	-3.151257	1.528851	-3.392448
H	-4.041085	1.529088	-0.899560
C	-2.970651	1.679094	-0.695477
H	-2.780943	1.133362	0.245367
C	-2.573198	3.146565	-0.602376
H	-1.518603	3.254694	-0.284765
H	-2.641170	3.650227	-1.588737
C	-3.469071	3.926752	0.373336
O	-4.469907	3.426523	0.882640
C	-3.033921	5.354382	0.678598
H	-2.076868	5.336778	1.234305
H	-2.849456	5.922619	-0.251793
H	-3.799841	5.862485	1.284342

1-cycle-6-C

SCF done: -3050.896022 A.U.

Pd	1.454549	0.712782	4.706613
P	-0.565991	2.017578	3.926294
S	0.659684	2.358229	7.134967
O	0.750520	0.845967	6.741176
O	0.749492	2.514943	8.609184
O	1.577057	3.196585	6.297205
O	1.828658	5.768645	4.901405
O	-2.631649	5.414024	3.429360
O	-0.082290	-1.740259	1.361827
O	-2.154982	-0.574477	5.432711
C	-1.060431	2.772674	6.671359
C	-1.881864	3.154885	7.745661
H	-1.422097	3.249220	8.733873
C	-3.253532	3.367909	7.555164
H	-3.885570	3.656722	8.402881
C	-3.808548	3.175837	6.282201
H	-4.886638	3.295833	6.121784
C	-2.985458	2.812943	5.205809
H	-3.439445	2.646412	4.226260
C	-1.593081	2.631242	5.360426
C	2.797635	6.058133	5.912572
H	3.764474	5.744772	5.489159
H	2.591788	5.475524	6.827604
H	2.837618	7.142125	6.141430
C	0.516103	6.013173	5.200592
C	0.088271	6.734986	6.334907
H	0.805606	7.079186	7.083088
C	-1.274543	7.015135	6.490249
H	-1.609070	7.578463	7.369055
C	-2.221317	6.598320	5.544174
H	-3.276872	6.840537	5.685641
C	-1.786461	5.856942	4.427777
C	-0.423036	5.522602	4.257214
C	-3.996477	5.825713	3.502377
H	-4.479261	5.421737	2.598976
H	-4.086644	6.929659	3.505481
H	-4.497547	5.416129	4.400889

C	0.058483	4.810085	3.030516
C	0.619111	5.630458	2.020753
H	0.655441	6.710175	2.203440
C	1.111488	5.109372	0.820619
H	1.529719	5.779239	0.060242
C	1.058381	3.723228	0.604917
H	1.428015	3.288255	-0.331447
C	0.517968	2.891983	1.592100
H	0.484929	1.811121	1.414702
C	0.001918	3.408546	2.809798
C	0.788301	-2.571532	0.598778
H	0.690580	-2.228010	-0.442950
H	0.497004	-3.638877	0.661551
H	1.843249	-2.467776	0.922353
C	-0.169688	-1.989952	2.715492
C	0.584369	-2.979790	3.376235
H	1.295279	-3.606240	2.832144
C	0.423285	-3.146796	4.759325
H	1.019947	-3.903089	5.280702
C	-0.473762	-2.361044	5.491379
H	-0.567711	-2.502181	6.569713
C	-1.227429	-1.374101	4.822251
C	-1.080726	-1.165048	3.425943
C	-2.269607	-0.667797	6.860707
H	-3.004800	0.100615	7.142134
H	-1.300563	-0.452067	7.344399
H	-2.641711	-1.665901	7.165259
C	-1.938734	-0.184108	2.687831
C	-2.903285	-0.711191	1.796474
H	-2.987571	-1.799810	1.709560
C	-3.733385	0.116355	1.032496
H	-4.470747	-0.326453	0.352743
C	-3.606499	1.510841	1.134402
H	-4.239251	2.173397	0.532367
C	-2.656596	2.055466	2.007978
H	-2.543619	3.143808	2.072317
C	-1.826101	1.228350	2.803247
C	3.119931	-0.043750	5.447005
C	4.263498	0.958257	5.193859
H	3.891874	1.971876	5.427823
H	5.101353	0.713706	5.872293
C	4.759198	0.881284	3.735558
H	5.304723	-0.074955	3.577840
C	3.711445	0.924842	2.631437
O	2.498478	0.702482	2.804349
O	3.252306	-1.118966	5.987725
H	5.504381	1.674916	3.535058
C	4.200447	1.230003	1.232802
H	4.265305	2.330377	1.121145
H	5.208970	0.818580	1.052483
H	3.488790	0.851022	0.483757

TS-2$_{Coor\text{-}E\text{-}C}$

SCF done: -3129.444686 A.U.

Pd	0.031284	1.670199	5.662841
P	-1.597696	2.508668	3.824124
S	-2.337553	3.020441	7.264074
O	-1.821447	1.621440	6.853913
O	-3.247582	2.912433	8.438801
O	-1.230531	4.029188	7.357229
O	2.522480	4.298030	2.195718
O	-1.229885	5.881438	4.561600
O	-3.990081	-0.191679	5.516343
O	-5.240644	1.475565	1.292124
C	-3.398639	3.558287	5.869419
C	-4.542155	4.268047	6.283795
H	-4.732811	4.350237	7.358200
C	-5.411725	4.843482	5.350072
H	-6.295991	5.394340	5.690952
C	-5.138010	4.704405	3.982544
H	-5.801480	5.149531	3.232371
C	-4.011414	3.982367	3.567634
H	-3.820551	3.884088	2.495850
C	-3.118242	3.387660	4.487223
C	3.889160	4.503890	1.844298
H	4.005090	4.076650	0.835865
H	4.583878	3.985087	2.534806
H	4.145987	5.581221	1.823274
C	2.060315	4.915880	3.338191
C	2.916598	5.435083	4.331061
H	4.003126	5.373275	4.224688
C	2.353339	6.055814	5.457324
H	3.012335	6.462058	6.234175
C	0.967171	6.188090	5.598896
H	0.549053	6.679711	6.479447
C	0.119175	5.691864	4.582982
C	0.648786	5.009634	3.455746
C	-1.813188	6.666341	5.614915
H	-2.882531	6.727706	5.365710
H	-1.373912	7.683100	5.633423
H	-1.686163	6.162490	6.588726
C	-0.223728	4.602289	2.306356
C	-0.033576	5.338088	1.110504
H	0.707968	6.144028	1.116597
C	-0.754368	5.069270	-0.056336
H	-0.578126	5.664517	-0.959871
C	-1.695126	4.029613	-0.054829
H	-2.267221	3.790708	-0.958858
C	-1.903671	3.289872	1.115508
H	-2.635795	2.475575	1.096756
C	-1.194411	3.559844	2.313824
C	-4.236250	-0.405399	6.914523
H	-3.310488	-0.844991	7.309612
H	-5.081251	-1.106174	7.064465
H	-4.425548	0.548182	7.438481
C	-4.979502	0.396494	4.780017
C	-6.266983	0.691282	5.280130
H	-6.518978	0.490306	6.323580
C	-7.225633	1.238466	4.419406
H	-8.226894	1.461266	4.805974
C	-6.934570	1.510672	3.074575
H	-7.700776	1.942097	2.426001
C	-5.641609	1.230795	2.591842
C	-4.644416	0.680850	3.432439
C	-6.228345	1.957378	0.381930
H	-5.720788	2.041610	-0.591425

H	-6.613453	2.952456	0.680945
H	-7.079047	1.253464	0.292644
C	-3.332775	0.246470	2.847763
C	-3.378355	-0.969529	2.117757
H	-4.340038	-1.491848	2.061131
C	-2.257798	-1.503974	1.476891
H	-2.337984	-2.445031	0.920860
C	-1.033620	-0.820948	1.559143
H	-0.138104	-1.214567	1.064322
C	-0.961172	0.369022	2.288713
H	0.002433	0.889495	2.358744
C	-2.093041	0.933588	2.935791
C	0.998382	1.472179	7.363298
O	0.920029	0.556411	8.149133
C	1.885175	2.721891	7.541260
H	1.370761	3.603694	7.124767
H	2.027911	2.875435	8.625623
C	3.246177	2.505664	6.861094
H	3.846726	3.434042	6.933719
H	3.836607	1.725208	7.382869
C	3.181349	2.135858	5.384188
O	2.116473	2.038133	4.755164
C	4.496159	1.842520	4.687799
H	5.239521	2.635327	4.887811
H	4.920030	0.899179	5.084521
H	4.335773	1.733722	3.604769
C	0.806727	-0.949398	4.998183
H	0.942048	-0.937277	3.911369
H	1.707420	-1.061471	5.613285
C	-0.424523	-0.865061	5.563857
H	-0.550292	-0.928109	6.649449
H	-1.338434	-0.819288	4.961670

2-Coor-E-C

SCF done: -3129.471134 A.U.

Pd	2.577473	1.723316	3.814533
P	0.084897	2.490455	3.615044
S	1.829828	2.928570	6.545841
O	2.160228	1.518384	5.971021
O	2.208635	3.006311	7.984104
O	2.351482	4.004480	5.641245
O	1.828775	6.544433	4.317152
O	-2.730156	5.426736	3.997730
O	0.867809	-1.150796	0.906811
O	-0.558080	-0.418811	5.340950
C	0.010027	2.972929	6.473378
C	-0.634197	3.124785	7.712485
H	-0.013924	3.269407	8.601898
C	-2.030990	3.056968	7.799606
H	-2.525007	3.167744	8.771642
C	-2.780547	2.819164	6.639066
H	-3.871482	2.723443	6.692435
C	-2.135864	2.688066	5.399552
H	-2.739348	2.483364	4.512401
C	-0.732568	2.786244	5.276322
C	2.963649	7.020308	5.047899
H	3.817499	6.920503	4.360704
H	3.138407	6.399697	5.944010
H	2.845666	8.085458	5.330459
C	0.626841	6.517775	4.967614
C	0.401687	7.085987	6.239678
H	1.222943	7.524624	6.810567
C	-0.896401	7.089083	6.764223
H	-1.073492	7.532586	7.750928
C	-1.977893	6.548006	6.054173
H	-2.982132	6.578540	6.482764
C	-1.738644	5.963521	4.794854
C	-0.433703	5.903021	4.253740
C	-4.077890	5.591032	4.438084
H	-4.707050	5.171573	3.637591
H	-4.329262	6.660060	4.581776
H	-4.268977	5.041991	5.380795
C	-0.192873	5.374220	2.873498
C	-0.128157	6.348135	1.847053
H	-0.261666	7.397118	2.133424
C	0.096034	6.014140	0.508256
H	0.139314	6.798644	-0.255758
C	0.264074	4.665222	0.160844
H	0.437322	4.370892	-0.880601
C	0.204856	3.686382	1.158829
H	0.316002	2.635403	0.867695
C	-0.027103	4.007835	2.523067
C	1.676263	-1.844519	-0.045574
H	1.303073	-1.533573	-1.033465
H	1.575177	-2.942355	0.058588
H	2.745234	-1.568692	0.045466
C	1.096500	-1.412123	2.243441
C	2.138324	-2.237423	2.707174
H	2.833252	-2.715955	2.013439
C	2.293655	-2.421976	4.088444
H	3.114144	-3.048595	4.454657
C	1.429642	-1.817928	5.008438
H	1.574212	-1.976626	6.078907
C	0.380018	-1.002449	4.534612
C	0.207981	-0.768438	3.144929
C	-0.409436	-0.587580	6.757245
H	-1.232074	-0.011421	7.205730
H	0.558909	-0.182972	7.100974
H	-0.503437	-1.654009	7.042229
C	-0.971250	-0.005788	2.622280
C	-1.958406	-0.755414	1.937827
H	-1.808896	-1.835746	1.837517
C	-3.100138	-0.156540	1.395843
H	-3.844424	-0.769487	0.874425
C	-3.279667	1.230291	1.519177
H	-4.163966	1.718700	1.093847
C	-2.313694	1.993661	2.185982
H	-2.444609	3.078857	2.262151
C	-1.158921	1.397961	2.752443
C	2.992170	1.317450	1.695050
C	3.427727	2.613879	1.984054
H	3.696207	0.477784	1.698006
H	2.024750	1.127484	1.217696
C	4.456203	1.146925	4.300825
H	4.488511	2.825706	2.162927
H	2.793475	3.483670	1.775966

C	5.181728	2.142993	5.203372
O	4.906800	0.078865	3.944050
C	5.361939	1.572054	6.624352
H	4.621401	3.090623	5.249901
H	6.175356	2.342345	4.757685
H	4.378072	1.370066	7.081582
H	5.900742	0.602930	6.582498
C	6.169904	2.525156	7.514234
O	6.941434	3.356584	7.042257
C	5.926942	2.387758	9.010020
H	4.894609	2.725499	9.225357
H	5.988215	1.330849	9.330460
H	6.646412	2.999511	9.575706

$TS\text{-}2_{Ins\text{-}E\text{-}C}$

SCF done: -3129.45960 A.U.

Pd	2.301643	1.598767	3.519644
P	0.121546	2.473241	3.522220
S	2.104120	2.954191	6.296142
O	2.383802	1.532915	5.740002
O	2.602642	3.082811	7.694574
O	2.523761	4.019393	5.328950
O	1.945370	6.581527	4.136942
O	-2.599336	5.366687	4.100113
O	0.561782	-1.154436	0.642754
O	-0.391532	-0.464535	5.209945
C	0.284462	2.977904	6.374334
C	-0.263709	3.133917	7.657732
H	0.424638	3.299040	8.491986
C	-1.647803	3.042702	7.856779
H	-2.065185	3.155831	8.863848
C	-2.482922	2.778152	6.762533
H	-3.564243	2.664267	6.903052
C	-1.936902	2.640819	5.477050
H	-2.606266	2.416187	4.643837
C	-0.548983	2.762122	5.244252
C	3.122831	7.047861	4.804243
H	3.933592	6.958766	4.065730
H	3.351098	6.412518	5.677504
H	3.021761	8.108306	5.110264
C	0.790377	6.522818	4.862990
C	0.632438	7.083154	6.148770
H	1.478392	7.539086	6.667746
C	-0.629979	7.058013	6.753354
H	-0.754609	7.496153	7.750426
C	-1.742362	6.496519	6.109852
H	-2.718700	6.506112	6.599435
C	-1.569347	5.919195	4.836735
C	-0.299221	5.884267	4.216319
C	-3.921924	5.521060	4.614967
H	-4.592013	5.100456	3.849005
H	-4.170549	6.588123	4.776077
H	-4.058409	4.967865	5.564699
C	-0.136027	5.363317	2.822212
C	-0.124967	6.345097	1.800863
H	-0.224317	7.393017	2.103931
C	0.001900	6.019197	0.448030
H	0.004490	6.809894	-0.310857
C	0.119993	4.670779	0.078197
H	0.210340	4.383548	-0.975623
C	0.118673	3.683850	1.068833
H	0.199208	2.635316	0.763431
C	-0.004688	4.000152	2.447248
C	1.234715	-1.866254	-0.397758
H	0.777785	-1.519511	-1.337309
H	1.093091	-2.960038	-0.299089
H	2.319381	-1.641232	-0.414344
C	0.899990	-1.464678	1.945972
C	1.948414	-2.340464	2.289334
H	2.546158	-2.836047	1.520545
C	2.226631	-2.565196	3.646048
H	3.051368	-3.231578	3.920273
C	1.481644	-1.948629	4.656869
H	1.728756	-2.131161	5.704362
C	0.428399	-1.078691	4.304477
C	0.129105	-0.810601	2.942509
C	-0.075130	-0.622377	6.601723
H	-0.821145	-0.017546	7.137834
H	0.938365	-0.239686	6.815175
H	-0.164960	-1.682004	6.911664
C	-1.060100	0.010246	2.548535
C	-2.127011	-0.686422	1.930053
H	-2.022862	-1.767510	1.789437
C	-3.290030	-0.038088	1.503362
H	-4.095420	-0.613089	1.032009
C	-3.412965	1.349265	1.677969
H	-4.313199	1.877001	1.342913
C	-2.369579	2.062788	2.279596
H	-2.460704	3.148162	2.399134
C	-1.191784	1.414450	2.730626
C	2.676629	1.606989	1.504386
C	3.937665	1.009401	1.953262
H	1.968441	0.948105	0.983088
H	2.693945	2.642648	1.137503
C	4.477621	0.969454	3.804476
H	4.036546	-0.071324	1.794701
H	4.842737	1.582808	1.698215
C	5.166671	2.242002	4.270689
O	4.695503	-0.152380	4.205506
C	5.682311	2.123136	5.710973
H	4.463836	3.091807	4.183792
H	6.005886	2.461720	3.581005
H	4.850143	1.879724	6.394391
H	6.402025	1.283239	5.795394
C	6.388129	3.402332	6.170003
O	6.887417	4.190214	5.368265
C	6.408322	3.638475	7.672452
H	5.368449	3.799693	8.017443
H	6.781250	2.745467	8.208810
H	7.029074	4.514054	7.917634

2-cycle-5-T

SCF done: -3129.487386 A.U.

Pd	1.624960	1.749886	4.720828
P	-0.342993	2.461270	3.880706
S	0.130331	2.310328	7.437015

O	0.728673	1.163013	6.590998
O	-0.285178	1.785595	8.767716
O	0.970759	3.543570	7.430290
O	1.619144	5.477432	4.996381
O	-2.841381	6.264229	3.713766
O	1.457309	-1.078473	1.962658
O	-1.545601	-0.717220	5.573732
C	-1.438440	2.737001	6.577926
C	-2.516588	2.963526	7.455102
H	-2.335496	2.838117	8.526737
C	-3.784465	3.306404	6.971334
H	-4.612018	3.467325	7.671865
C	-3.985035	3.428677	5.589253
H	-4.972281	3.683385	5.187084
C	-2.917513	3.205959	4.710938
H	-3.096522	3.305127	3.638321
C	-1.627456	2.862048	5.176882
C	2.704456	5.615425	5.923147
H	3.594341	5.252192	5.384736
H	2.527314	4.997350	6.818721
H	2.859461	6.677547	6.198846
C	0.393313	5.941748	5.393030
C	0.143025	6.545368	6.643347
H	0.920032	6.584928	7.408238
C	-1.130222	7.058138	6.910337
H	-1.327721	7.517148	7.885838
C	-2.163138	6.994301	5.962543
H	-3.147155	7.405762	6.198618
C	-1.907123	6.379766	4.722882
C	-0.638494	5.821482	4.429130
C	-4.123612	6.844388	3.945865
H	-4.700094	6.673811	3.023096
H	-4.051507	7.933426	4.136751
H	-4.639931	6.360483	4.798173
C	-0.346048	5.284403	3.061731
C	-0.159918	6.252625	2.045824
H	-0.279241	7.306639	2.319403
C	0.182155	5.906355	0.734711
H	0.325438	6.687509	-0.020689
C	0.351747	4.553341	0.405505
H	0.632238	4.254447	-0.610952
C	0.152399	3.575015	1.386484
H	0.269148	2.521753	1.111345
C	-0.217454	3.910554	2.715222
C	2.574100	-1.708668	1.335157
H	2.540027	-1.393293	0.280769
H	2.504118	-2.812547	1.393624
H	3.535715	-1.384822	1.780901
C	1.212778	-1.397079	3.284717
C	2.076067	-2.184364	4.070782
H	3.010642	-2.579153	3.665034
C	1.716194	-2.461738	5.399042
H	2.386761	-3.069110	6.017886
C	0.524878	-1.981092	5.952009
H	0.278431	-2.197733	6.993223
C	-0.334489	-1.193723	5.157290
C	0.008618	-0.871430	3.819266
C	-1.916993	-0.952829	6.939654
H	-2.903884	-0.480399	7.055668
H	-1.200503	-0.482082	7.635934
H	-2.002295	-2.037871	7.147441
C	-0.986016	-0.202904	2.917095
C	-1.739379	-1.075005	2.094731
H	-1.547117	-2.150169	2.178673
C	-2.704383	-0.607347	1.197441
H	-3.268913	-1.314927	0.579365
C	-2.931941	0.772558	1.092429
H	-3.672261	1.166611	0.386867
C	-2.196195	1.654440	1.891937
H	-2.353172	2.729655	1.764044
C	-1.226027	1.195105	2.818321
C	2.709806	2.107230	3.016295
C	4.172397	2.337784	3.461214
H	2.607863	1.177077	2.425120
H	2.353659	2.959274	2.419280
C	4.524079	1.496246	4.659290
H	4.911566	2.195840	2.643621
H	4.301057	3.390368	3.795491
C	5.952703	1.079628	4.924588
O	3.606845	1.143096	5.433849
C	6.209100	0.520624	6.327925
H	6.626616	1.921736	4.675188
H	6.210067	0.284692	4.188402
H	6.530302	1.305407	7.038636
H	5.257861	0.126810	6.742576
C	7.219000	-0.632310	6.330383
O	7.566260	-1.190717	5.291612
C	7.748224	-1.060303	7.692398
H	8.424181	-0.279098	8.090715
H	6.925715	-1.170135	8.422863
H	8.305228	-2.005301	7.603255

Alternating pathway, path B

TS-1$_{Coor\text{-}CO\text{-}T}$
SCF done: -3050.888016 A.U.

Pd	1.562491	1.123374	4.148115
P	-0.398458	2.206129	3.893055
S	0.640248	1.215081	7.159945
O	0.827708	0.248305	5.970809
O	0.345217	0.451686	8.402524
O	1.715088	2.253427	7.236095
O	2.778517	4.910758	1.516482
O	0.612334	5.040453	5.696090
O	-1.280069	-1.664285	5.065211
O	-4.625199	1.517579	4.142668
C	-0.891115	2.147103	6.770505
C	-1.638634	2.503677	7.908571
H	-1.322177	2.099307	8.875077
C	-2.747677	3.352135	7.807289
H	-3.310448	3.627683	8.706685
C	-3.121348	3.849453	6.550509
H	-3.970993	4.534961	6.451133
C	-2.405557	3.467032	5.408261
H	-2.718699	3.858757	4.438015
C	-1.286543	2.605820	5.487433

C	3.941437	5.111786	0.714178
H	3.588661	5.093857	-0.328497
H	4.687704	4.305618	0.859625
H	4.419416	6.088382	0.925586
C	2.929549	4.963858	2.888153
C	4.181180	5.091005	3.524078
H	5.104687	5.163007	2.943843
C	4.226689	5.138208	4.927364
H	5.197055	5.224519	5.429707
C	3.060867	5.087888	5.696074
H	3.119670	5.118305	6.785266
C	1.810149	4.982673	5.048736
C	1.726265	4.870218	3.635932
C	0.630648	5.260650	7.117178
H	-0.428142	5.324779	7.407538
H	1.143304	6.212740	7.357046
H	1.109863	4.410841	7.633352
C	0.403504	4.861377	2.929175
C	0.101934	6.033144	2.191557
H	0.841363	6.840733	2.192494
C	-1.092334	6.189183	1.482106
H	-1.284708	7.116311	0.929993
C	-2.034889	5.151454	1.488412
H	-2.979298	5.247205	0.940752
C	-1.761531	3.979459	2.203298
H	-2.501083	3.172347	2.192112
C	-0.556204	3.805800	2.931751
C	-0.566251	-2.516942	5.973426
H	0.311923	-2.860964	5.409704
H	-1.187797	-3.384982	6.271735
H	-0.230809	-1.953781	6.861095
C	-2.363497	-0.993116	5.552551
C	-2.929747	-1.222626	6.825652
H	-2.471931	-1.930251	7.519680
C	-4.089239	-0.530523	7.192856
H	-4.529826	-0.711861	8.180072
C	-4.701087	0.390784	6.330140
H	-5.606024	0.915927	6.644748
C	-4.116971	0.630134	5.071145
C	-2.936452	-0.039228	4.672631
C	-5.864742	2.148606	4.459066
H	-6.118767	2.765523	3.582891
H	-5.776588	2.797824	5.352432
H	-6.668078	1.405186	4.630333
C	-2.411249	0.117869	3.277249
C	-2.983077	-0.745605	2.310514
H	-3.757530	-1.443287	2.648083
C	-2.590404	-0.735073	0.968976
H	-3.056777	-1.422545	0.254073
C	-1.590846	0.159129	0.554470
H	-1.258906	0.184829	-0.489633
C	-1.012461	1.022993	1.488603
H	-0.235498	1.718160	1.154085
C	-1.411511	1.031885	2.850486
C	2.371220	1.614760	2.327439
H	2.041844	0.795181	1.660650
H	2.058670	2.578226	1.898834
C	3.901632	1.578864	2.524899
H	4.472168	1.522309	1.572379
H	4.232307	2.518507	3.021894
C	4.299993	0.469360	3.458680
O	3.470621	0.060396	4.301982
C	5.680328	-0.139054	3.409149
H	6.451659	0.653232	3.411388
H	5.835126	-0.819552	4.259851
H	5.803278	-0.699434	2.462279
C	1.254154	-2.026103	2.885083
O	1.310515	-1.962302	1.736402

1-Coor-CO-T

SCF done: -3050.905472 A.U.

P	0.062158	-0.355692	0.670962
S	1.148079	-0.882799	-2.628390
O	-0.339350	-1.281625	-2.420726
O	1.723994	-1.584001	-3.803899
O	1.329057	0.599654	-2.549672
O	2.044171	2.941728	-0.870962
O	3.918158	0.173113	2.438259
O	-4.269228	-0.677670	1.268572
O	-1.148630	-3.677670	-0.569871
C	1.951894	-1.612438	-1.155833
C	3.024124	-2.475553	-1.439854
H	3.304110	-2.612216	-2.488562
C	3.686534	-3.161003	-0.413410
H	4.512801	-3.839994	-0.653331
C	3.267138	-2.987734	0.912219
H	3.752297	-3.536331	1.727692
C	2.204874	-2.119400	1.200906
H	1.887874	-2.012320	2.239724
C	1.532139	-1.404354	0.185515
C	2.063847	3.547401	-2.170488
H	1.150153	4.159245	-2.220938
H	2.037688	2.775799	-2.957999
H	2.949036	4.202627	-2.292404
C	3.073676	2.094455	-0.557101
C	4.195914	1.873235	-1.382444
H	4.255730	2.320003	-2.376514
C	5.233120	1.056804	-0.917925
H	6.102426	0.879645	-1.561487
C	5.189721	0.462084	0.352049
H	6.018798	-0.162212	0.692600
C	4.060707	0.680862	1.163732
C	2.974056	1.465831	0.708211
C	5.041867	-0.509612	2.993941
H	4.755343	-0.764926	4.026176
H	5.940914	0.137048	3.010669
H	5.272569	-1.438280	2.436027
C	1.838222	1.792270	1.629203
C	2.028635	2.923378	2.457908
H	2.980069	3.459856	2.372882
C	1.054725	3.363570	3.361071
H	1.240176	4.245844	3.984356
C	-0.154265	2.660115	3.458643
H	-0.937861	2.981637	4.153126
C	-0.362035	1.529732	2.659059
H	-1.299629	0.978045	2.773296

C	0.616242	1.071122	1.737279
C	-5.496286	0.054107	1.216643
H	-5.570163	0.577276	2.181700
H	-6.366006	-0.620349	1.092041
H	-5.489919	0.800479	0.399318
C	-3.935838	-1.437843	0.172224
C	-4.721651	-1.530540	-0.994077
H	-5.659914	-0.978729	-1.088610
C	-4.279661	-2.351339	-2.043749
H	-4.885955	-2.426600	-2.953760
C	-3.084747	-3.076032	-1.959415
H	-2.761976	-3.698182	-2.796615
C	-2.304295	-2.981904	-0.787062
C	-2.712678	-2.150344	0.286558
C	-0.622407	-4.445959	-1.661177
H	0.328322	-4.859007	-1.292706
H	-0.433651	-3.801394	-2.537110
H	-1.304656	-5.276255	-1.930714
C	-1.989453	-2.170975	1.599570
C	-2.558756	-2.989290	2.604632
H	-3.461023	-3.557280	2.353549
C	-2.009288	-3.083687	3.886983
H	-2.477723	-3.729653	4.638254
C	-0.866330	-2.334289	4.203897
H	-0.428919	-2.377685	5.207720
C	-0.287525	-1.515030	3.228137
H	0.577310	-0.900782	3.500116
C	-0.820105	-1.428573	1.916470
Pd	-1.319426	0.288195	-1.215960
C	-2.419065	0.790676	-2.705947
O	-3.055455	1.066604	-3.632193
H	-3.313414	1.858077	-0.443136
C	-2.264864	1.796636	-0.110459
H	-2.272131	1.464160	0.936750
C	-1.539270	3.124549	-0.286129
H	-0.454012	3.036223	-0.079024
H	-1.599093	3.479956	-1.336228
C	-2.107018	4.245815	0.603329
O	-3.113785	4.096863	1.291011
C	-1.330974	5.561691	0.588343
H	-0.332075	5.405853	1.037655
H	-1.164751	5.916039	-0.446040
H	-1.876463	6.326930	1.162258

TS-1$_{Isom-CO}$

SCF done: -3050.87798 A.U.

Pd	-0.873920	-1.621761	0.502601
P	0.365301	0.943385	0.332168
S	-0.005631	-0.916640	-2.604189
O	-0.036512	-1.935165	-1.419104
O	0.347645	-1.654003	-3.843869
O	-1.236507	-0.070863	-2.637954
O	-3.016102	2.148535	2.062064
O	-1.766233	3.045242	-2.383059
O	1.457526	-2.674041	0.976038
O	4.812412	0.641863	0.758670
C	1.385920	0.238800	-2.282964
C	2.260381	0.372847	-3.377502
H	2.103344	-0.275483	-4.244187
C	3.287629	1.324737	-3.361530
H	3.949628	1.427778	-4.229095
C	3.445881	2.149036	-2.239326
H	4.227231	2.917966	-2.217455
C	2.591871	1.996951	-1.138135
H	2.735909	2.647636	-0.273135
C	1.546859	1.046243	-1.123452
C	-3.956119	1.682442	3.030996
H	-3.505839	1.901747	4.011144
H	-4.127440	0.591708	2.944888
H	-4.925493	2.211670	2.945145
C	-3.360314	2.021559	0.733203
C	-4.558048	1.427476	0.285687
H	-5.290123	1.027997	0.991772
C	-4.803694	1.358373	-1.094940
H	-5.732322	0.895832	-1.449445
C	-3.892538	1.864952	-2.029418
H	-4.101877	1.784540	-3.098216
C	-2.703397	2.471552	-1.569969
C	-2.420492	2.550504	-0.185000
C	-1.918286	2.866336	-3.796619
H	-1.038020	3.350304	-4.246674
H	-2.836544	3.361638	-4.170723
H	-1.924900	1.792405	-4.052460
C	-1.240354	3.327199	0.316141
C	-1.435536	4.714250	0.514289
H	-2.406712	5.144152	0.244733
C	-0.435482	5.528971	1.055772
H	-0.617700	6.600328	1.199032
C	0.787909	4.956771	1.437481
H	1.573379	5.571433	1.892493
C	1.001419	3.587121	1.241018
H	1.942252	3.144650	1.585231
C	0.014385	2.757173	0.653580
C	1.118625	-4.060222	0.763186
H	0.325967	-4.285820	1.491795
H	2.000715	-4.699772	0.951108
H	0.740017	-4.218984	-0.261939
C	2.591820	-2.216051	0.312709
C	3.205410	-2.924952	-0.736950
H	2.767377	-3.843863	-1.130329
C	4.384050	-2.416096	-1.296446
H	4.861775	-2.957459	-2.120550
C	4.960781	-1.227177	-0.829915
H	5.882542	-0.855823	-1.283630
C	4.327813	-0.523403	0.213042
C	3.120524	-0.995225	0.786080
C	6.059619	1.126696	0.260723
H	6.283171	2.030076	0.848936
H	5.991124	1.392293	-0.812097
H	6.870618	0.385643	0.403061
C	2.497601	-0.300507	1.956861
C	3.055665	-0.574185	3.227218
H	3.938902	-1.221508	3.273290
C	2.504577	-0.055617	4.403948
H	2.958244	-0.288073	5.374099
C	1.353765	0.744428	4.323967

H	0.888183	1.143021	5.232784
C	0.796757	1.039518	3.074485
H	-0.102432	1.663873	3.027794
C	1.367657	0.556056	1.867175
C	-2.796237	-1.600948	-0.326778
H	-3.523609	-1.329511	0.448870
H	-2.620651	-0.787287	-1.047764
C	-3.085665	-2.931772	-0.981180
H	-3.984262	-2.803920	-1.632067
H	-2.277373	-3.222566	-1.679050
C	-3.406855	-4.087533	-0.031263
O	-3.547990	-3.923209	1.178837
C	-3.544601	-5.464541	-0.672754
H	-4.187511	-5.427052	-1.571443
H	-2.550500	-5.815552	-1.010840
H	-3.952445	-6.183106	0.054703
C	-1.639688	-1.841622	2.191352
O	-2.056918	-1.902766	3.274186

1-Coor-CO-C

SCF done: -3050.902281 A.U.

P	-0.527463	0.776615	0.297964
S	-0.611896	-2.451900	-0.754936
O	0.873515	-1.958956	-0.851026
O	-0.797933	-3.695477	-1.541428
O	-1.069239	-2.443774	0.669213
O	-3.414124	-2.183697	2.320821
O	-4.331521	1.817015	0.015742
O	3.334457	3.195421	1.155144
O	1.308735	1.123174	-2.562171
C	-1.502420	-1.128282	-1.629728
C	-2.157977	-1.507828	-2.811970
H	-2.155200	-2.566913	-3.086661
C	-2.765882	-0.541561	-3.625164
H	-3.269954	-0.843842	-4.550149
C	-2.696204	0.810665	-3.259407
H	-3.133014	1.582635	-3.903731
C	-2.054417	1.186850	-2.069545
H	-1.992358	2.246541	-1.810935
C	-1.469966	0.228229	-1.213264
C	-3.436747	-3.609616	2.426741
H	-2.828881	-3.845846	3.313144
H	-2.981660	-4.075564	1.534834
H	-4.466611	-3.990658	2.578152
C	-4.039528	-1.619621	1.245552
C	-4.900071	-2.321854	0.375168
H	-5.050345	-3.398100	0.485268
C	-5.577679	-1.618835	-0.628267
H	-6.251845	-2.161566	-1.300927
C	-5.423527	-0.234232	-0.785676
H	-5.976803	0.291154	-1.567215
C	-4.540107	0.452684	0.070430
C	-3.805671	-0.230739	1.066852
C	-5.163583	2.567645	-0.869299
H	-4.897857	3.623554	-0.705826
H	-6.236201	2.417302	-0.639056
H	-4.977725	2.302881	-1.928420
C	-2.938402	0.504894	2.041497
C	-3.511883	0.744153	3.314768
H	-4.530074	0.380217	3.490019
C	-2.827248	1.415201	4.331530
H	-3.307541	1.578113	5.303053
C	-1.523509	1.875218	4.091924
H	-0.963584	2.407306	4.869161
C	-0.934615	1.647809	2.844441
H	0.079673	2.024522	2.669028
C	-1.617703	0.968444	1.803569
C	4.464810	3.434284	1.997051
H	4.140780	4.204859	2.713043
H	5.331108	3.809765	1.418100
H	4.760691	2.522720	2.550522
C	3.482270	2.284988	0.133714
C	4.657247	1.537650	-0.082918
H	5.529069	1.657270	0.564330
C	4.693458	0.619584	-1.142271
H	5.602750	0.031620	-1.310158
C	3.592825	0.434171	-1.987787
H	3.646579	-0.288849	-2.804263
C	2.422226	1.191960	-1.769275
C	2.342151	2.119495	-0.698406
C	1.288159	0.118400	-3.587240
H	0.291913	0.195532	-4.046792
H	1.419157	-0.887470	-3.150360
H	2.065710	0.312727	-4.351662
C	1.144995	2.999081	-0.502853
C	1.327454	4.381132	-0.749801
H	2.313881	4.719119	-1.084367
C	0.295603	5.308923	-0.575005
H	0.475354	6.370887	-0.778389
C	-0.962072	4.871979	-0.130774
H	-1.778886	5.586527	0.022418
C	-1.168468	3.510642	0.124552
H	-2.146799	3.171058	0.482742
C	-0.135229	2.559497	-0.064776
Pd	1.385563	-0.740853	0.820611
C	2.106553	0.124136	2.294139
O	2.567770	0.630422	3.234683
C	2.880364	-2.212593	1.143208
C	2.264352	-3.369047	1.913597
H	2.038143	-3.084281	2.963287
H	1.291071	-3.673116	1.483166
C	3.182674	-4.605815	1.990470
O	4.375860	-4.561018	1.708231
C	2.505872	-5.899568	2.438917
H	1.852500	-5.731955	3.314876
H	1.856384	-6.272565	1.623756
H	3.265115	-6.662610	2.671009
H	3.089567	-2.461926	0.091438
H	3.785466	-1.811011	1.620951

$TS_{Ins\text{-}CO\text{-}C}$

SCF done: -3050.88904 A.U.

P	-0.528993	0.771032	0.261822
S	-0.589505	-2.468574	-0.803844
O	0.883805	-1.976827	-0.936860
O	-0.815009	-3.717151	-1.573185

O	-1.022245	-2.446913	0.630962
O	-3.392004	-2.188705	2.270430
O	-4.332635	1.841714	0.027011
O	3.334339	3.170323	1.131899
O	1.296510	1.169388	-2.615416
C	-1.501324	-1.141714	-1.661376
C	-2.167143	-1.517837	-2.838701
H	-2.161546	-2.576161	-3.116805
C	-2.788252	-0.551775	-3.642645
H	-3.298860	-0.853622	-4.564261
C	-2.724466	0.799496	-3.272759
H	-3.172342	1.571173	-3.909740
C	-2.074112	1.174586	-2.087126
H	-2.017665	2.233258	-1.822668
C	-1.475016	0.214665	-1.242288
C	-3.406773	-3.616508	2.353290
H	-2.787579	-3.863854	3.228730
H	-2.958943	-4.065440	1.449234
H	-4.433172	-4.004533	2.510716
C	-4.031410	-1.610439	1.211462
C	-4.903128	-2.300857	0.342638
H	-5.051377	-3.378628	0.439720
C	-5.593560	-1.584304	-0.642218
H	-6.276442	-2.117860	-1.313441
C	-5.440434	-0.197806	-0.783821
H	-6.002382	0.337741	-1.552141
C	-4.545600	0.477311	0.069733
C	-3.800986	-0.218885	1.049062
C	-5.164783	2.603194	-0.848585
H	-4.891313	3.656356	-0.680241
H	-6.237095	2.458531	-0.613258
H	-4.986737	2.343575	-1.910344
C	-2.919588	0.500077	2.023770
C	-3.481068	0.740684	3.301828
H	-4.506905	0.399751	3.478955
C	-2.772943	1.381400	4.322440
H	-3.244743	1.546916	5.297750
C	-1.455526	1.802627	4.085014
H	-0.875296	2.302012	4.868955
C	-0.877473	1.574961	2.832694
H	0.153281	1.904321	2.660082
C	-1.589189	0.934301	1.786924
C	4.442825	3.341681	2.018328
H	4.120232	4.098550	2.749278
H	5.340818	3.706720	1.482418
H	4.685845	2.401547	2.548674
C	3.475131	2.270942	0.102167
C	4.641233	1.510174	-0.119600
H	5.513178	1.612987	0.530416
C	4.670838	0.606666	-1.192300
H	5.575224	0.012639	-1.366388
C	3.571973	0.446156	-2.045494
H	3.620018	-0.266005	-2.871854
C	2.409231	1.215002	-1.820164
C	2.336369	2.127165	-0.735962
C	1.252230	0.154626	-3.630941
H	0.256442	0.247970	-4.088497
H	1.363498	-0.848278	-3.181634
H	2.030911	0.325652	-4.399769
C	1.139920	3.002793	-0.523098
C	1.317022	4.388415	-0.750333
H	2.301128	4.734558	-1.083380
C	0.282428	5.310052	-0.558167
H	0.457929	6.375403	-0.746962
C	-0.971908	4.863085	-0.114800
H	-1.789974	5.572963	0.052565
C	-1.172689	3.497842	0.124120
H	-2.147487	3.150302	0.484471
C	-0.137199	2.553917	-0.085136
Pd	1.312916	-0.630078	0.718317
C	2.214213	-0.086914	2.238609
O	2.552318	0.433006	3.241565
C	3.092873	-1.757295	1.619510
C	2.576133	-2.922665	2.453503
H	2.252632	-2.586044	3.461078
H	1.677837	-3.377837	1.995089
C	3.650284	-4.003432	2.676859
O	4.837990	-3.796650	2.446287
C	3.144510	-5.342167	3.202217
H	2.441548	-5.204545	4.044492
H	2.583505	-5.861663	2.401651
H	3.992543	-5.971070	3.514081
H	3.245416	-2.023771	0.557947
H	4.047454	-1.351630	1.982942

1-cycle-6-T

SCF done: -3050.91479 A.U.

P	-0.286854	0.921341	0.037909
S	-0.239391	-2.007019	-1.929872
O	1.112958	-1.260963	-2.005799
O	-0.434600	-2.915339	-3.090280
O	-0.476451	-2.595929	-0.569687
O	-2.185753	-2.505695	1.851825
O	-4.405540	1.424843	0.521771
O	3.627056	3.045619	0.631670
O	1.100544	1.863161	-3.155682
C	-1.430254	-0.629257	-2.154746
C	-2.302255	-0.784989	-3.246010
H	-2.251192	-1.723690	-3.805719
C	-3.169832	0.246233	-3.628975
H	-3.832833	0.111856	-4.491531
C	-3.155867	1.454885	-2.919910
H	-3.795780	2.290271	-3.227488
C	-2.302216	1.608321	-1.817888
H	-2.295158	2.564493	-1.290409
C	-1.439810	0.571983	-1.394567
C	-1.978364	-3.920211	1.772634
H	-1.190654	-4.141728	2.509988
H	-1.633967	-4.205122	0.763403
H	-2.895515	-4.478528	2.047129
C	-3.133955	-1.968113	1.021494
C	-3.985053	-2.732568	0.195792
H	-3.866400	-3.815002	0.116843
C	-4.989123	-2.085011	-0.533490
H	-5.649775	-2.678128	-1.176282
C	-5.174833	-0.697255	-0.457273

H	-5.974335	-0.220954	-1.029056
C	-4.306330	0.059147	0.353207
C	-3.254990	-0.557180	1.071828
C	-5.515931	2.076858	-0.093604
H	-5.447475	3.133056	0.210313
H	-6.478760	1.655103	0.255934
H	-5.467283	2.007807	-1.197869
C	-2.402226	0.239670	2.010314
C	-2.857759	0.310938	3.349127
H	-3.792242	-0.204761	3.596765
C	-2.157808	1.005064	4.340510
H	-2.542734	1.036152	5.366389
C	-0.960043	1.655450	4.004915
H	-0.388662	2.204722	4.761796
C	-0.491560	1.604072	2.688086
H	0.445628	2.113126	2.441938
C	-1.197310	0.909208	1.670661
C	4.788471	2.930977	1.452720
H	4.578346	3.536531	2.347463
H	5.689777	3.328307	0.944201
H	4.961222	1.881685	1.755346
C	3.608478	2.331790	-0.540790
C	4.705512	1.599503	-1.038049
H	5.645039	1.546052	-0.482796
C	4.576438	0.934976	-2.267107
H	5.425590	0.361184	-2.656097
C	3.391385	0.985881	-3.010554
H	3.315986	0.448798	-3.957952
C	2.294848	1.714129	-2.503257
C	2.382116	2.378229	-1.254297
C	0.911032	1.112704	-4.363243
H	-0.124642	1.318180	-4.672459
H	1.037411	0.032414	-4.173750
H	1.607610	1.450638	-5.156143
C	1.261626	3.231489	-0.745298
C	1.429702	4.632471	-0.850188
H	2.353000	5.006695	-1.305305
C	0.463271	5.532878	-0.389681
H	0.628276	6.611934	-0.489523
C	-0.709285	5.044338	0.206748
H	-1.474436	5.733276	0.582514
C	-0.895584	3.662160	0.330196
H	-1.801179	3.287746	0.820098
C	0.070060	2.740149	-0.146248
Pd	1.517852	-0.516776	0.050339
C	1.989731	-0.297715	1.940245
O	2.622781	0.586414	2.487376
C	1.652290	-1.636050	2.643171
C	2.823729	-2.628548	2.492334
H	3.699502	-2.257965	3.067712
H	2.568829	-3.607878	2.940065
C	3.340449	-2.859686	1.075345
O	3.130876	-2.080360	0.132917
C	4.179249	-4.096974	0.839503
H	4.873364	-4.282469	1.678707
H	3.502388	-4.971616	0.777085
H	4.730484	-4.012111	-0.108895
H	1.494463	-1.428586	3.716969
H	0.725588	-2.056336	2.216924

TS-2$_{Coor\text{-}E\text{-}T}$

SCF done: -3129.451196 A.U.

P	-0.367505	0.348855	0.117145
S	-0.402754	-2.337156	-2.228344
O	0.844935	-1.439059	-2.326756
O	-0.704412	-3.011020	-3.518624
O	-0.365840	-3.245801	-1.022907
O	-2.793055	-2.491458	1.191345
O	-4.227543	1.856762	2.297833
O	1.984386	1.187945	-2.927297
O	-2.226362	3.292701	-2.718043
C	-1.775787	-1.147069	-1.946788
C	-2.898160	-1.358126	-2.766381
H	-2.861383	-2.185001	-3.482016
C	-4.011484	-0.509847	-2.692143
H	-4.879182	-0.693494	-3.336512
C	-4.000895	0.577465	-1.809144
H	-4.863991	1.247412	-1.737551
C	-2.880692	0.801387	-0.996538
H	-2.864130	1.669276	-0.329935
C	-1.760580	-0.058497	-1.034170
C	-3.022276	-3.778457	0.602311
H	-2.051847	-4.289976	0.622898
H	-3.340297	-3.682393	-0.451552
H	-3.779191	-4.348104	1.176812
C	-3.832740	-1.601359	1.178674
C	-5.120665	-1.903210	0.685218
H	-5.343058	-2.887638	0.268282
C	-6.121712	-0.926109	0.740193
H	-7.122202	-1.162693	0.359927
C	-5.873730	0.347699	1.272136
H	-6.672448	1.092892	1.304102
C	-4.585851	0.638509	1.763052
C	-3.544899	-0.323194	1.717334
C	-5.248390	2.846168	2.425661
H	-4.763432	3.712507	2.900958
H	-6.081717	2.493261	3.064607
H	-5.652267	3.149306	1.439384
C	-2.256960	-0.044138	2.438023
C	-2.387680	-0.101524	3.850244
H	-3.374736	-0.351679	4.254525
C	-1.325629	0.143225	4.723828
H	-1.478642	0.083441	5.807436
C	-0.067844	0.455174	4.189858
H	0.792616	0.647060	4.840223
C	0.090822	0.497406	2.800932
H	1.085725	0.714292	2.402433
C	-0.976336	0.260388	1.892646
C	2.953032	0.403133	-3.635987
H	3.851847	0.405316	-3.002813
H	3.194715	0.859473	-4.616004
H	2.592037	-0.631628	-3.765231
C	0.764591	1.355800	-3.527321
C	0.455465	0.861319	-4.813020
H	1.162085	0.227667	-5.351208
C	-0.779202	1.172085	-5.390552

H	-1.019890	0.777667	-6.384300
C	-1.712592	1.980474	-4.726719
H	-2.665108	2.223690	-5.202955
C	-1.399789	2.458018	-3.440661
C	-0.180586	2.116457	-2.797372
C	-3.401399	3.770092	-3.369059
H	-3.868576	4.473432	-2.661773
H	-4.109985	2.947734	-3.591142
H	-3.158062	4.302236	-4.309823
C	0.132428	2.757303	-1.474296
C	0.545673	4.107041	-1.560014
H	0.678385	4.532233	-2.561006
C	0.763776	4.903993	-0.430829
H	1.089753	5.944265	-0.544067
C	0.535139	4.355686	0.836529
H	0.662681	4.960343	1.741846
C	0.138436	3.016988	0.952508
H	-0.044191	2.623653	1.954423
C	-0.048924	2.182988	-0.176178
Pd	1.475683	-1.062552	-0.192280
C	1.913567	-1.551602	1.661142
O	2.791961	-1.114978	2.378243
C	1.105537	-2.833050	1.970039
C	1.766404	-4.097647	1.376634
H	2.632662	-4.395473	2.006761
H	1.057415	-4.943973	1.416123
C	2.343820	-4.003268	-0.033862
O	2.752214	-2.941759	-0.532221
C	2.473006	-5.294201	-0.806545
H	2.696682	-6.155569	-0.153118
H	1.485240	-5.468322	-1.277480
H	3.226408	-5.198648	-1.603056
H	1.049810	-2.935841	3.069235
H	0.087817	-2.726564	1.561545
C	3.740982	0.887618	-0.050169
C	4.653124	-0.087243	-0.225733
H	3.361241	1.498514	-0.876049
H	3.363842	1.121433	0.951001
H	5.061351	-0.337460	-1.212456
H	5.007321	-0.691812	0.616511

2-Coor-E-T

SCF done: -3129.477575 A.U.

P	0.226462	-0.393646	0.126016
S	0.107579	-3.815933	-0.278465
O	1.535546	-3.391301	-0.679389
O	-0.242542	-5.194917	-0.697703
O	-0.133424	-3.474583	1.178115
O	-2.128536	-2.563571	3.096533
O	-3.849434	0.442664	-0.086856
O	4.274080	1.903926	-0.174214
O	1.728687	-0.874974	-2.996979
C	-0.929477	-2.676624	-1.250842
C	-1.729084	-3.281743	-2.234235
H	-1.742564	-4.374819	-2.283195
C	-2.457420	-2.499411	-3.140465
H	-3.070167	-2.980769	-3.911286
C	-2.369520	-1.102343	-3.066061
H	-2.899531	-0.472334	-3.790063
C	-1.589053	-0.498271	-2.068303
H	-1.525584	0.591558	-2.041247
C	-0.869824	-1.263090	-1.123609
C	-1.998819	-3.878219	3.662161
H	-1.265417	-3.772868	4.475484
H	-1.621085	-4.586625	2.904871
H	-2.964888	-4.228058	4.076090
C	-2.951371	-2.415917	2.014240
C	-3.805960	-3.427255	1.526954
H	-3.790071	-4.429075	1.960953
C	-4.683095	-3.130451	0.476849
H	-5.348019	-3.915344	0.098328
C	-4.739372	-1.851820	-0.096805
H	-5.444231	-1.645561	-0.905479
C	-3.867826	-0.854962	0.383448
C	-2.938889	-1.131698	1.412593
C	-4.852351	0.803719	-1.036184
H	-4.711424	1.879378	-1.225007
H	-5.870214	0.630423	-0.635391
H	-4.734315	0.245450	-1.985260
C	-2.082072	-0.046998	1.989446
C	-2.623509	0.636471	3.106491
H	-3.621872	0.341025	3.447437
C	-1.931490	1.655777	3.767245
H	-2.388851	2.167881	4.621780
C	-0.647093	2.009383	3.322993
H	-0.078375	2.802266	3.822357
C	-0.090758	1.347449	2.222723
H	0.909751	1.638774	1.886010
C	-0.793277	0.328223	1.526855
C	5.505391	2.273371	0.447058
H	5.273276	3.158009	1.059295
H	6.278888	2.536302	-0.301564
H	5.888761	1.466909	1.101459
C	4.279932	0.801701	-0.994794
C	5.431826	0.037942	-1.272147
H	6.398066	0.294037	-0.831211
C	5.323522	-1.067760	-2.129129
H	6.216509	-1.664523	-2.348678
C	4.098453	-1.433288	-2.701221
H	4.039540	-2.302492	-3.359515
C	2.952408	-0.653035	-2.431178
C	3.024024	0.472397	-1.571172
C	1.567570	-2.059854	-3.789968
H	0.510199	-2.062402	-4.092696
H	1.783122	-2.961370	-3.189786
H	2.210689	-2.027781	-4.691438
C	1.877146	1.428597	-1.444069
C	2.062957	2.680878	-2.081888
H	3.013025	2.856434	-2.597491
C	1.083090	3.677198	-2.073696
H	1.266811	4.630841	-2.581849
C	-0.130552	3.442798	-1.410048
H	-0.914782	4.208132	-1.388568
C	-0.335220	2.218373	-0.764139
H	-1.278834	2.050609	-0.234260
C	0.648858	1.195372	-0.768607

Pd	1.923749	-1.933801	1.067515
C	2.072783	-0.976239	2.803795
O	2.858796	-0.083174	3.048541
C	1.122110	-1.606683	3.827093
C	1.125570	-0.867690	5.171997
H	2.162498	-0.593805	5.456651
H	0.586910	0.096041	5.092620
C	0.530894	-1.689464	6.315643
O	0.264279	-2.884966	6.204067
C	0.289938	-0.940231	7.623150
H	1.163098	-0.322160	7.901900
H	-0.564921	-0.248102	7.497699
H	0.057268	-1.653314	8.428877
H	0.106001	-1.670988	3.396976
H	1.434111	-2.659355	3.968807
C	4.125790	-2.270246	1.017053
C	3.543585	-3.333016	1.708342
H	4.605037	-1.443129	1.550346
H	4.316308	-2.336100	-0.059294
H	3.582492	-3.384529	2.803113
H	3.254437	-4.251176	1.185168

TS-2$_{Ins-E-T}$

SCF done: -3129.450040 A.U.

C	4.118288	-1.339334	0.637301
C	3.499845	-0.120116	1.000267
C	4.021674	1.085336	0.466564
C	5.062937	1.060793	-0.485579
C	5.620845	-0.169268	-0.854404
C	5.173946	-1.374134	-0.294637
C	2.447789	-0.079204	2.066574
C	1.061061	-0.328743	1.886352
C	0.206223	-0.260270	3.017576
C	0.691595	0.036907	4.295922
C	2.060977	0.287151	4.475056
C	2.913455	0.228543	3.369010
P	0.089334	-0.696774	0.325956
C	1.222237	-1.243780	-1.063680
C	1.596167	-0.392198	-2.138330
C	2.387296	-0.868845	-3.197879
C	2.793683	-2.208928	-3.238431
C	2.390339	-3.078645	-2.215975
C	1.618652	-2.597330	-1.147978
S	1.009681	1.323335	-2.343734
O	1.526798	1.802233	-3.650594
O	3.475773	2.235180	0.971647
C	3.877175	3.466764	0.367566
O	3.617739	-2.460416	1.267865
C	4.300807	-3.694328	1.045657
C	-0.712013	-2.303812	0.832811
C	-2.025572	-2.669422	0.430483
C	-2.533391	-3.920969	0.855459
C	-1.786811	-4.792636	1.654570
C	-0.496123	-4.422495	2.062251
C	0.027847	-3.189662	1.655158
C	-2.952547	-1.825902	-0.393687
C	-4.158267	-1.342889	0.187931
C	-5.113395	-0.653732	-0.589987
C	-4.880408	-0.484540	-1.963814
C	-3.720376	-0.975910	-2.576854
C	-2.761462	-1.652211	-1.790984
O	-4.313320	-1.617266	1.520031
C	-5.432504	-1.040365	2.193458
O	-1.624192	-2.218621	-2.294439
C	-1.310914	-1.969585	-3.672888
Pd	-1.431676	1.035376	-0.442963
C	-2.269114	1.820040	1.569303
C	-1.279583	2.822241	2.189180
C	-0.256411	3.514782	1.280106
C	-0.747160	4.777269	0.582332
O	-1.908291	5.184781	0.688466
C	-3.274597	2.441228	0.249568
C	-2.788814	2.432150	-1.130164
O	-0.537943	1.140051	-2.417048
O	1.424381	2.096735	-1.127408
O	-2.823456	0.974018	2.265115
C	0.277685	5.503415	-0.275395
H	2.646980	-0.169912	-3.998259
H	3.401497	-2.571964	-4.075111
H	2.664429	-4.139811	-2.247900
H	1.301500	-3.297671	-0.372430
H	3.317021	4.251102	0.899703
H	3.607888	3.484878	-0.703402
H	4.962960	3.648321	0.495735
H	5.440015	1.984683	-0.929331
H	6.431156	-0.190035	-1.592220
H	5.636311	-2.318502	-0.590078
H	3.795892	-4.434287	1.685784
H	5.367442	-3.623754	1.335158
H	4.230844	-4.016329	-0.011684
H	3.985301	0.418063	3.494133
H	2.462180	0.523484	5.467340
H	-0.001726	0.071752	5.144146
H	-0.866712	-0.445339	2.895843
H	-5.341914	-1.362221	3.241835
H	-6.393140	-1.405479	1.779227
H	-5.395930	0.063773	2.147491
H	-6.036686	-0.278520	-0.141913
H	-5.629343	0.034564	-2.573429
H	-3.566705	-0.837222	-3.649096
H	-0.335628	-2.450360	-3.837734
H	-1.226738	-0.885074	-3.864958
H	-2.066997	-2.424762	-4.342337
H	-3.547109	-4.198533	0.547722
H	-2.214852	-5.753162	1.963892
H	0.100744	-5.086759	2.697828
H	1.032541	-2.900161	1.981590
H	0.147857	2.827119	0.503716
H	0.639704	3.783978	1.875275
H	1.089382	5.907231	0.359728
H	0.747493	4.790495	-0.978638
H	-0.199117	6.330899	-0.822743
H	-0.760547	2.259181	2.982643
H	-1.908292	3.588381	2.688284
H	-3.379698	1.891383	-1.881841
H	-2.228546	3.296832	-1.508279

H	-4.195099	1.865324	0.409768
H	-3.278443	3.461615	0.683389

2-cycle-5-C

SCF done: -3129.46950 A.U.

Pd	1.715354	1.541274	4.739232
P	-0.414604	2.240035	3.865915
S	0.277419	2.324671	7.350402
O	0.813023	1.088113	6.551097
O	-0.069696	1.853331	8.718123
O	1.161044	3.516459	7.231331
O	1.775711	5.282490	4.629562
O	-2.685976	6.096723	3.371682
O	0.975188	-1.379211	1.897274
O	-1.598209	-0.783719	5.792343
C	-1.327491	2.766126	6.567196
C	-2.321415	3.101429	7.507679
H	-2.071185	3.028403	8.570028
C	-3.601430	3.489646	7.096070
H	-4.363083	3.738673	7.843863
C	-3.899721	3.542236	5.727253
H	-4.900648	3.829298	5.384707
C	-2.916055	3.205666	4.789419
H	-3.169854	3.244285	3.728211
C	-1.612309	2.820360	5.176371
C	2.892991	5.473620	5.508000
H	3.744218	4.992103	5.002792
H	2.711203	4.986602	6.480366
H	3.111556	6.551462	5.643780
C	0.580315	5.836624	5.002149
C	0.382955	6.572736	6.189322
H	1.183577	6.671661	6.924415
C	-0.866996	7.153016	6.430855
H	-1.023765	7.717856	7.357036
C	-1.926304	7.026949	5.519341
H	-2.890550	7.492796	5.735387
C	-1.721834	6.282707	4.341978
C	-0.479050	5.659963	4.076622
C	-3.937975	6.751968	3.566326
H	-4.541720	6.512837	2.676814
H	-3.816930	7.850633	3.642861
H	-4.455552	6.381291	4.473083
C	-0.223625	4.973649	2.769992
C	0.044153	5.814466	1.663303
H	-0.007976	6.897585	1.820178
C	0.380672	5.305476	0.404596
H	0.578075	5.988685	-0.429731
C	0.472620	3.915207	0.230293
H	0.742060	3.490001	-0.743981
C	0.196504	3.063617	1.306582
H	0.261471	1.980537	1.156098
C	-0.183642	3.566765	2.577568
C	2.017917	-2.037948	1.184502
H	1.879582	-1.760334	0.127858
H	1.952407	-3.139338	1.286983
H	3.022374	-1.707252	1.519067
C	0.875064	-1.633269	3.250500
C	1.807538	-2.404304	3.973608
H	2.675357	-2.852367	3.482607
C	1.593302	-2.610282	5.346502
H	2.314857	-3.206807	5.916695
C	0.479319	-2.076268	6.002748
H	0.346845	-2.237616	7.074352
C	-0.451783	-1.310539	5.268641
C	-0.257086	-1.064507	3.886520
C	-1.827162	-0.953569	7.198771
H	-2.788644	-0.456615	7.396246
H	-1.034293	-0.467982	7.794741
H	-1.907072	-2.026660	7.462680
C	-1.309752	-0.387452	3.059285
C	-2.180297	-1.243017	2.342722
H	-2.055802	-2.324627	2.465527
C	-3.171245	-0.746748	1.489429
H	-3.828893	-1.438763	0.950770
C	-3.299015	0.639738	1.315950
H	-4.051448	1.050570	0.632912
C	-2.449780	1.503417	2.017439
H	-2.528567	2.580601	1.837744
C	-1.463681	1.017088	2.911347
C	3.554778	1.043719	5.522444
C	4.449909	0.649855	4.329767
H	3.902829	1.965969	6.021878
H	3.437101	0.235792	6.260350
C	4.079501	1.468283	3.125934
H	5.541566	0.695792	4.530293
H	4.232562	-0.403951	4.046738
C	5.082657	1.816844	2.055501
O	2.893106	1.882289	3.031223
C	4.498548	2.477344	0.802059
H	5.679968	0.914205	1.819880
H	5.818977	2.522021	2.503614
H	4.252026	1.737436	0.016734
H	3.533450	2.964644	1.051953
C	5.422796	3.554724	0.221276
O	6.415991	3.950444	0.827093
C	5.022836	4.123707	-1.133282
H	5.117109	3.345094	-1.914419
H	3.963799	4.441665	-1.124844
H	5.669518	4.975946	-1.391176

Catalyst Pd-4

Non-alternating pathway

1-cycle5-T

SCF done: -2339.315424 A.U.

Pd	-0.673151	4.820496	8.475500
S	-2.893564	5.729437	6.575818
P	-1.386894	2.774999	7.808275
O	-1.341631	5.636934	6.589808
C	-4.006470	0.919646	10.683733
H	-3.439554	1.178794	11.599893
H	-5.072073	1.102607	10.918096
C	0.848714	2.673744	5.905809
H	1.195994	3.614962	6.384806
C	-2.744367	2.836200	6.529914
C	-2.085034	1.589864	9.158720
H	-1.450367	1.826824	10.037021

O	-3.461721	5.976873	7.935898
C	-0.005914	3.101918	4.672445
H	-0.928564	3.559856	5.064055
O	-3.360313	6.603444	5.471347
C	-3.578844	1.873327	9.535896
H	-4.172259	1.594385	8.640004
C	0.107901	1.876663	7.034562
H	-0.259613	0.914161	6.622461
C	-3.180965	1.611931	5.968684
H	-2.673013	0.681558	6.237876
C	2.352368	0.784039	7.746863
H	2.003723	-0.188399	7.336233
C	-0.403247	1.932633	3.748743
H	-1.139682	2.273399	2.999332
H	-0.865180	1.094826	4.302727
H	0.467358	1.532161	3.196885
C	3.070170	1.546792	6.620598
H	3.930514	0.958786	6.245466
H	3.487827	2.488762	7.034762
C	-4.883469	2.734288	4.656866
H	-5.710318	2.704456	3.938408
C	-3.384507	4.026321	6.090171
C	-2.316547	-0.841976	10.003843
H	-1.683603	-0.580010	10.879346
C	-3.937159	3.357378	9.866869
H	-3.364876	4.019074	9.189660
C	2.104130	1.868346	5.470167
H	1.780570	0.915791	5.003985
H	2.637346	2.431598	4.682498
C	-3.588470	3.787044	11.307799
H	-3.652037	4.886485	11.393671
H	-2.566670	3.487211	11.605232
H	-4.289139	3.355025	12.046688
C	-1.914028	0.080005	8.829321
H	-2.561050	-0.184775	7.971076
H	-0.878559	-0.155637	8.528524
C	-4.239712	1.553014	5.050609
H	-4.549511	0.584812	4.641675
C	-2.070639	-2.320293	9.654053
H	-2.674609	-2.625073	8.778074
H	-2.349237	-2.979091	10.496702
H	-1.008486	-2.511326	9.413168
C	3.279659	0.497866	8.940920
H	4.166108	-0.082302	8.625754
H	2.761580	-0.078111	9.730029
H	3.641246	1.440448	9.394802
C	-5.424759	3.642609	9.575000
H	-5.674130	3.419215	8.522296
H	-5.649878	4.709352	9.746911
H	-6.095338	3.044742	10.222680
C	-3.784760	-0.568952	10.375917
H	-4.432881	-0.881772	9.530383
H	-4.082166	-1.187200	11.245380
C	1.113887	1.594529	8.188681
H	1.459099	2.573700	8.578422
H	0.626642	1.079992	9.037322
C	-4.441072	3.963277	5.164079
H	-4.885276	4.905925	4.829614
C	0.692766	4.218950	3.867736
H	1.642078	3.880791	3.410577
H	0.901376	5.093273	4.509126
H	0.037905	4.562258	3.046619
C	0.043122	4.283262	10.321267
H	-0.788145	4.535246	11.005897
H	0.310151	3.224294	10.468917
C	1.260529	5.191625	10.603607
H	1.545140	5.231313	11.676824
H	2.159931	4.810152	10.072405
C	1.027204	6.576596	10.051646
O	0.248575	6.715030	9.083256
C	1.724257	7.778494	10.638656
H	1.326419	7.971787	11.653719
H	2.806284	7.582792	10.755244
H	1.562582	8.667148	10.010365

TS-1$_{\text{Coor-E-T}}$

SCF done: -2417.864396 A.U.

Pd	-1.217189	5.067808	8.452957
S	-3.819855	5.261712	6.648114
P	-1.566831	2.869943	7.886510
O	-2.359376	5.777720	6.756986
C	-4.072495	0.899826	10.836324
H	-3.378796	0.836881	11.698203
H	-5.058045	1.175669	11.253384
C	0.969862	3.113100	6.494417
H	1.198902	3.933278	7.207014
C	-2.698042	2.631970	6.403644
C	-2.248345	1.636575	9.207621
H	-1.452156	1.644270	9.980399
O	-4.462092	5.032849	7.980163
C	0.288075	3.793964	5.263913
H	-0.727010	4.104077	5.574280
O	-4.578408	6.065874	5.658087
C	-3.596991	2.045149	9.896376
H	-4.348375	2.161470	9.088368
C	0.088852	2.096591	7.297505
H	-0.168320	1.253123	6.627786
C	-2.635439	1.409025	5.689249
H	-1.920768	0.637786	5.986035
C	2.217587	0.851530	8.081888
H	1.975222	0.011234	7.395619
C	0.150033	2.874394	4.032828
H	-0.490389	3.354899	3.271947
H	-0.311235	1.901193	4.279209
H	1.130134	2.675606	3.560244
C	3.079313	1.859262	7.301934
H	4.016940	1.378466	6.960835
H	3.381349	2.681698	7.985948
C	-4.380826	2.105497	4.154436
H	-5.030995	1.911058	3.294192
C	-3.617062	3.602537	5.924541
C	-2.772722	-0.879857	9.621348
H	-2.040673	-0.892772	10.457946
C	-3.562630	3.396725	10.682208
H	-3.062316	4.133991	10.026175
C	2.308026	2.432841	6.103327

H	2.102498	1.609230	5.390452
H	2.940014	3.160103	5.560800
C	-2.775403	3.333133	12.009802
H	-2.604699	4.354541	12.396922
H	-1.783839	2.854588	11.902176
H	-3.328780	2.781234	12.792053
C	-2.348485	0.206174	8.609004
H	-3.107913	0.212965	7.804266
H	-1.392301	-0.091979	8.137303
C	-3.466470	1.138857	4.591589
H	-3.384396	0.174012	4.078604
C	-2.781659	-2.270715	8.963156
H	-3.497198	-2.304700	8.119547
H	-3.082236	-3.049589	9.687617
H	-1.784178	-2.542510	8.570110
C	2.954751	0.276899	9.304073
H	3.895958	-0.218631	9.003594
H	2.336564	-0.467471	9.838989
H	3.213129	1.077715	10.023331
C	-4.986927	3.933761	10.934994
H	-5.525065	4.080218	9.984211
H	-4.941916	4.913713	11.445306
H	-5.577995	3.259331	11.583539
C	-4.145109	-0.490977	10.192869
H	-4.894619	-0.501252	9.373991
H	-4.480951	-1.237132	10.939414
C	0.888071	1.521199	8.498502
H	1.122628	2.338627	9.208804
H	0.283005	0.785994	9.058586
C	-4.440588	3.338029	4.816049
H	-5.113343	4.133394	4.481023
C	1.012423	5.102666	4.878791
H	2.053044	4.923059	4.546754
H	1.030806	5.809028	5.727705
H	0.483249	5.600079	4.046268
C	-0.061469	4.663002	10.108230
H	-0.566459	5.282177	10.872659
H	-0.076964	3.620046	10.463268
C	1.389417	5.130842	9.906052
H	1.932757	5.223302	10.871670
H	1.966286	4.378106	9.325186
C	1.510617	6.414740	9.109014
O	0.623625	6.735028	8.302594
C	2.741954	7.280220	9.295559
H	2.710744	7.756208	10.294907
H	3.662679	6.668226	9.266372
H	2.783597	8.063743	8.523819
C	-2.727692	6.935973	9.928396
H	-2.552113	6.760975	10.998303
H	-3.581899	6.425268	9.468302
C	-1.954683	7.789576	9.221084
H	-2.142676	7.976201	8.158346
H	-1.119795	8.334412	9.677045

1-Coor-E-T

SCF done: -2417.870539 A.U.

Pd	-0.586731	5.142777	8.274920
S	-2.922893	5.767352	6.515259
P	-1.420156	2.919302	7.830784
O	-1.387941	5.726509	6.288821
C	-4.040656	0.945021	10.654537
H	-3.567559	1.297170	11.591707
H	-5.133615	1.030625	10.805183
C	0.744502	2.633991	5.820925
H	1.106906	3.630496	6.152697
C	-2.794487	2.896990	6.568299
C	-2.085888	1.750173	9.232803
H	-1.550756	2.111795	10.135355
O	-3.227508	5.965594	7.976032
C	-0.165723	2.893570	4.580449
H	-1.079222	3.387466	4.948394
O	-3.607396	6.666352	5.555534
C	-3.625932	1.883753	9.489108
H	-4.123228	1.500850	8.573931
C	0.071378	1.989505	7.080956
H	-0.272201	0.967114	6.822697
C	-3.254400	1.650865	6.082930
H	-2.764052	0.729575	6.412165
C	2.369921	1.056299	7.840767
H	2.019021	0.027287	7.607940
C	-0.584149	1.614245	3.827244
H	-1.363292	1.850327	3.080525
H	-1.000560	0.843312	4.501030
H	0.266670	1.162659	3.284144
C	3.016004	1.637279	6.570716
H	3.859636	0.999787	6.241224
H	3.447948	2.631696	6.810816
C	-4.954845	2.722490	4.723603
H	-5.787813	2.663101	4.014253
C	-3.439361	4.065873	6.082439
C	-2.138537	-0.653454	10.199341
H	-1.617326	-0.287093	11.109489
C	-4.155363	3.335971	9.715784
H	-3.578886	4.028188	9.073930
C	1.984518	1.778552	5.440164
H	1.646316	0.765785	5.141305
H	2.466636	2.218535	4.547804
C	-3.996400	3.838198	11.166562
H	-4.177382	4.927840	11.206545
H	-2.988496	3.637326	11.570991
H	-4.725369	3.360148	11.847202
C	-1.723339	0.253474	9.017231
H	-2.236861	-0.127522	8.111841
H	-0.643024	0.120529	8.842402
C	-4.320497	1.557019	5.175192
H	-4.648394	0.572202	4.824120
C	-1.710223	-2.110246	9.948817
H	-2.203622	-2.519029	9.046066
H	-1.987125	-2.756588	10.801612
H	-0.617384	-2.194963	9.803017
C	3.363494	0.972858	9.013006
H	4.238502	0.350886	8.749514
H	2.894464	0.533780	9.912739
H	3.737357	1.977960	9.286491
C	-5.626489	3.461345	9.267728
H	-5.746391	3.194452	8.202164

H	-5.979869	4.500147	9.392987
H	-6.292853	2.806193	9.861545
C	-3.652867	-0.523822	10.433492
H	-4.191821	-0.932601	9.552604
H	-3.960918	-1.135938	11.303448
C	1.139080	1.909671	8.213799
H	1.476128	2.943722	8.428792
H	0.708060	1.531757	9.159132
C	-4.504800	3.972839	5.171259
H	-4.955824	4.902563	4.811156
C	0.485499	3.901223	3.608565
H	1.420205	3.513443	3.161531
H	0.711213	4.854145	4.119388
H	-0.205148	4.129258	2.776691
C	0.123707	4.682558	10.159650
H	0.018462	3.604282	10.350176
H	1.200661	4.931928	10.182965
C	-0.651307	5.467458	11.221843
H	-0.308881	6.514800	11.308774
H	-1.729474	5.525914	10.960075
C	-0.618202	4.789163	12.608501
O	-0.611447	3.566304	12.731265
C	-0.636533	5.719706	13.814274
H	0.304769	6.301148	13.853289
H	-1.458068	6.455258	13.725030
H	-0.748551	5.140733	14.743854
C	0.280158	7.207125	8.371742
H	0.369546	7.508737	9.420513
H	-0.429513	7.766421	7.753106
C	1.215375	6.357334	7.788489
H	1.262708	6.248702	6.698814
H	2.072594	5.977162	8.355624

TS-1$_{Isom-E}$
SCF done: -2417.845901 A.U.

S	0.997036	-2.553808	0.922177
P	-0.848970	0.179548	0.041518
O	1.894569	-1.290144	0.773393
C	-4.719557	-0.785239	-1.632963
H	-4.487357	-0.565839	-2.694306
H	-5.293861	-1.730696	-1.629807
C	0.813358	2.158235	1.487172
H	1.658672	1.723570	0.914235
C	-1.200903	-0.836165	1.575811
C	-2.593731	0.346474	-0.800285
H	-2.314598	0.615845	-1.841098
O	0.458807	-2.928726	-0.439529
C	0.808202	1.433366	2.870568
H	0.512855	0.388583	2.684175
O	1.657051	-3.627174	1.700849
C	-3.392663	-0.996357	-0.855020
H	-3.663099	-1.240939	0.193892
C	-0.464491	1.967953	0.603289
H	-1.338694	2.367466	1.154954
C	-2.260121	-0.501242	2.449935
H	-2.854766	0.395358	2.253101
C	-0.012398	4.260568	-0.549973
H	-0.870911	4.712490	-0.007125
C	-0.189119	2.028034	3.886556
H	-0.259130	1.376678	4.776325
H	-1.209098	2.124214	3.471033
H	0.127356	3.028270	4.235692
C	1.242303	4.430162	0.324003
H	1.436203	5.504017	0.513484
H	2.124091	4.043660	-0.228673
C	-1.797421	-2.409004	3.878818
H	-2.021785	-3.017527	4.761910
C	-0.434260	-1.987280	1.905974
C	-4.799049	1.678781	-1.059610
H	-4.519157	1.924967	-2.106957
C	-2.593556	-2.217422	-1.407696
H	-1.581323	-2.193640	-0.964193
C	1.091398	3.676481	1.653906
H	0.261839	4.135215	2.229041
H	2.003437	3.810644	2.263412
C	-2.410617	-2.203472	-2.940436
H	-1.646708	-2.944347	-3.237372
H	-2.083869	-1.215354	-3.316629
H	-3.345515	-2.463585	-3.470805
C	-3.493391	1.486788	-0.252070
H	-3.782828	1.267727	0.794684
H	-2.950281	2.448328	-0.230145
C	-2.562463	-1.272497	3.583319
H	-3.392954	-0.977213	4.234568
C	-5.630764	2.843719	-0.494761
H	-5.929602	2.644873	0.552330
H	-6.554732	2.994546	-1.082440
H	-5.063080	3.792516	-0.507728
C	0.124766	4.967637	-1.909686
H	0.319650	6.047805	-1.778569
H	-0.792353	4.860701	-2.518328
H	0.966392	4.544550	-2.490776
C	-3.219456	-3.553420	-0.956117
H	-3.295842	-3.605858	0.145176
H	-2.593262	-4.401410	-1.286307
H	-4.232045	-3.703806	-1.376986
C	-5.585043	0.356303	-1.078055
H	-5.911741	0.116354	-0.044137
H	-6.507118	0.465634	-1.681788
C	-0.300972	2.754945	-0.732556
H	0.540139	2.301004	-1.298199
H	-1.196135	2.633037	-1.371393
C	-0.728039	-2.759406	3.041830
H	-0.097947	-3.627808	3.256585
C	2.224893	1.364369	3.481000
H	2.623177	2.362900	3.740501
H	2.931016	0.878846	2.784357
H	2.210674	0.766979	4.410951
Pd	1.391128	-0.652675	-1.299887
C	1.003683	-0.225839	-3.350763
H	1.380279	0.766478	-3.629476
C	1.889623	-1.329593	-3.261810
H	-0.065481	-0.388920	-3.526823
H	2.955318	-1.214253	-3.489447
H	1.507627	-2.357161	-3.301845
C	3.208615	0.329822	-1.366118

H	3.358802	0.765475	-2.368051
H	3.023003	1.135861	-0.636820
C	4.339912	-0.581925	-0.929382
H	4.043895	-1.144015	-0.019568
H	4.577556	-1.350529	-1.690786
C	5.631456	0.186605	-0.583056
O	5.669151	1.410174	-0.497110
C	6.864990	-0.680093	-0.340346
H	6.637828	-1.512603	0.350501
H	7.196853	-1.136449	-1.292736
H	7.680953	-0.062620	0.065905

1-Coor-E-C

SCF done: -2417.867314 A.U.

Pd	-0.778048	5.219284	8.045895
S	-3.335423	5.301370	6.334529
P	-1.402220	2.751749	7.614790
O	-1.828146	5.649089	6.179359
C	-4.034829	1.321433	10.755731
H	-3.342710	1.190719	11.610822
H	-4.957000	1.767978	11.170730
C	1.056345	2.547334	6.087941
H	1.387521	3.433895	6.666059
C	-2.620650	2.540761	6.199973
C	-2.174936	1.679759	9.030470
H	-1.347365	1.625859	9.767786
O	-3.710875	5.251037	7.790983
C	0.391723	3.119016	4.794833
H	-0.581758	3.553696	5.086923
O	-4.181298	6.133488	5.444628
C	-3.405851	2.329475	9.751481
H	-4.163004	2.553430	8.972181
C	0.121599	1.731982	7.047400
H	-0.234438	0.831982	6.507441
C	-2.751453	1.290366	5.548938
H	-2.115359	0.453013	5.847676
C	2.194153	0.458368	7.963438
H	1.886162	-0.459716	7.417255
C	0.135295	2.064744	3.698977
H	-0.478821	2.498809	2.889828
H	-0.406108	1.180859	4.081006
H	1.079634	1.713008	3.243525
C	3.092973	1.280063	7.024003
H	3.989101	0.692930	6.743490
H	3.462668	2.176577	7.565871
C	-4.497278	2.127355	4.083262
H	-5.222050	1.974190	3.275989
C	-3.443800	3.593539	5.719028
C	-3.048870	-0.700390	9.626893
H	-2.311540	-0.770696	10.455769
C	-3.088126	3.677310	10.480575
H	-2.497569	4.291944	9.772087
C	2.321191	1.710508	5.767050
H	2.028349	0.801924	5.203072
H	2.982186	2.291429	5.097354
C	-2.259393	3.516068	11.774747
H	-1.877806	4.499327	12.103251
H	-1.387287	2.845920	11.649225
H	-2.866379	3.109095	12.604357
C	-2.487271	0.242304	8.540915
H	-3.247231	0.297006	7.738538
H	-1.585507	-0.215674	8.089331
C	-3.676363	1.077480	4.514615
H	-3.745405	0.088385	4.047850
C	-3.281913	-2.112689	9.062269
H	-4.015903	-2.090935	8.234203
H	-3.676710	-2.791076	9.840537
H	-2.346453	-2.555095	8.671784
C	2.928141	0.030613	9.246574
H	3.825824	-0.569153	9.009688
H	2.279577	-0.578197	9.903312
H	3.259448	0.913688	9.825694
C	-4.373593	4.477798	10.776075
H	-4.922557	4.694517	9.844970
H	-4.119703	5.442740	11.252510
H	-5.044894	3.939978	11.472120
C	-4.335091	-0.070980	10.184967
H	-5.088708	-0.005126	9.372612
H	-4.773142	-0.714924	10.972529
C	0.911741	1.253877	8.297958
H	1.196156	2.132294	8.915298
H	0.273528	0.620319	8.939265
C	-4.366531	3.388315	4.679613
H	-4.961415	4.243396	4.344991
C	1.214725	4.295420	4.225850
H	2.219978	3.978512	3.889128
H	1.338029	5.093786	4.980142
H	0.699801	4.739398	3.355043
C	-0.589885	7.292906	8.172434
H	0.247277	7.576509	8.831149
H	-0.378130	7.546761	7.121438
C	-1.905861	7.891521	8.654581
H	-2.096546	8.860134	8.141026
H	-2.774959	7.262489	8.361780
C	-2.013515	8.127560	10.158605
O	-1.161223	7.744034	10.962316
C	-3.259584	8.871481	10.628492
H	-3.284062	9.891893	10.201361
H	-4.171936	8.357173	10.273546
H	-3.269001	8.935162	11.727389
C	0.288974	5.047626	9.936974
H	-0.004112	5.940280	10.504790
H	0.053358	4.078402	10.389053
C	1.226304	5.129562	8.896754
H	1.730200	4.230529	8.525265
H	1.721010	6.075373	8.649889

TS-1$_{\mathbf{Ins\text{-}E\text{-}C}}$

SCF done: -2417.846906 A.U.

Pd	-0.466522	4.773745	8.414666
S	-2.724998	5.665543	6.441543
P	-1.316695	2.705784	7.732601
O	-1.176720	5.551598	6.481315
C	-3.945276	0.927919	10.651510
H	-3.363820	1.196447	11.555718
H	-5.004944	1.134488	10.893671

C	0.877394	2.467587	5.767784
H	1.300623	3.391354	6.218534
C	-2.683585	2.778546	6.461381
C	-2.027147	1.532559	9.093201
H	-1.380563	1.770052	9.963519
O	-3.303322	5.949363	7.798048
C	0.021513	2.930416	4.547085
H	-0.869776	3.437800	4.950123
O	-3.161888	6.549330	5.331768
C	-3.512361	1.847477	9.478479
H	-4.117441	1.556640	8.594183
C	0.121755	1.736086	6.931304
H	-0.297078	0.782595	6.548491
C	-3.175959	1.561151	5.933285
H	-2.711174	0.617178	6.231399
C	2.311156	0.520203	7.608438
H	1.881402	-0.436890	7.241005
C	-0.452232	1.775927	3.640130
H	-1.183460	2.148225	2.900596
H	-0.946201	0.967933	4.210231
H	0.387066	1.327023	3.077289
C	3.041712	1.199252	6.436603
H	3.840100	0.538761	6.045490
H	3.545965	2.114171	6.812788
C	-4.825232	2.718978	4.583634
H	-5.651079	2.705274	3.863711
C	-3.275028	3.979245	5.986207
C	-2.295229	-0.877995	9.986615
H	-1.646560	-0.612390	10.849383
C	-3.846311	3.344862	9.774497
H	-3.261724	3.979316	9.082056
C	2.062924	1.570982	5.311837
H	1.656855	0.638621	4.870701
H	2.608830	2.083581	4.498394
C	-3.492665	3.802485	11.205405
H	-3.543200	4.903874	11.270585
H	-2.474773	3.497967	11.511247
H	-4.196729	3.393558	11.953843
C	-1.888413	0.014145	8.790501
H	-2.548552	-0.255721	7.943678
H	-0.861192	-0.248318	8.484282
C	-4.235571	1.522921	5.014800
H	-4.588986	0.557889	4.634732
C	-2.082356	-2.367155	9.661986
H	-2.704843	-2.676977	8.800812
H	-2.360577	-3.004537	10.521027
H	-1.027646	-2.582623	9.409521
C	3.256246	0.207422	8.781399
H	4.088434	-0.444520	8.458853
H	2.725839	-0.304347	9.605729
H	3.698013	1.136138	9.190521
C	-5.329451	3.645948	9.475144
H	-5.580441	3.409541	8.425669
H	-5.543029	4.717593	9.632055
H	-6.008014	3.066054	10.130446
C	-3.753325	-0.570461	10.371638
H	-4.418126	-0.888458	9.541020
H	-4.050595	-1.165339	11.257226
C	1.147907	1.430207	8.060149
H	1.578341	2.396264	8.392557
H	0.661499	0.987131	8.948531
C	-4.330930	3.941210	5.058283
H	-4.735262	4.892361	4.698691
C	0.763349	4.001774	3.719173
H	1.690669	3.612444	3.257996
H	1.022703	4.873439	4.345871
H	0.117238	4.365359	2.899759
C	0.390825	6.910654	8.636294
H	1.289958	7.364498	9.073549
H	0.574571	6.749372	7.560406
C	-0.842549	7.772571	8.882765
H	-0.823700	8.616440	8.156566
H	-1.784485	7.240473	8.628664
C	-1.004301	8.383078	10.269120
O	-0.258920	8.125980	11.217591
C	-2.170162	9.354153	10.426678
H	-3.112933	8.889236	10.084128
H	-2.261147	9.663252	11.479202
H	-2.011310	10.249035	9.795422
C	0.935517	5.462407	10.154470
H	0.653975	6.273004	10.836416
H	1.989471	5.452725	9.850828
C	0.271832	4.192000	10.263164
H	0.871914	3.278363	10.169069
H	-0.570403	4.113293	10.963668

1-B-T

SCF done: -2417.8881 A.U.

Pd	-0.722886	4.807030	7.804104
S	-2.980771	5.465052	5.909723
P	-1.387733	2.667362	7.463414
O	-1.426672	5.438628	5.858558
C	-3.999122	1.175598	10.556020
H	-3.411157	1.506582	11.434913
H	-5.055978	1.405516	10.787575
C	0.906298	2.347006	5.665150
H	1.283887	3.309244	6.075971
C	-2.735275	2.575915	6.174751
C	-2.090242	1.654295	8.945206
H	-1.427147	1.961855	9.779955
O	-3.483966	5.845256	7.267985
C	0.088273	2.712228	4.388646
H	-0.823039	3.232876	4.729066
O	-3.541241	6.201305	4.750497
C	-3.568855	2.008197	9.317074
H	-4.183236	1.664151	8.458251
C	0.113890	1.677772	6.840362
H	-0.257640	0.684581	6.513356
C	-3.133814	1.289946	5.734671
H	-2.594154	0.406857	6.087763
C	2.301820	0.611155	7.720174
H	1.928278	-0.389590	7.412157
C	-0.336420	1.493125	3.544899
H	-1.050381	1.803084	2.761069
H	-0.833723	0.712131	4.148357
H	0.528347	1.026966	3.037182

C	3.066052	1.221004	6.530943
H	3.907357	0.563145	6.237687
H	3.516151	2.184188	6.851223
C	-4.884058	2.222581	4.341544
H	-5.716723	2.097289	3.640521
C	-3.415467	3.697795	5.624638
C	-2.360826	-0.680535	10.022923
H	-1.706240	-0.349772	10.858088
C	-3.882434	3.524015	9.515852
H	-3.323818	4.097890	8.752238
C	2.134175	1.458509	5.331527
H	1.782785	0.477186	4.953315
H	2.701933	1.923202	4.504608
C	-3.463802	4.078748	10.893677
H	-3.510889	5.182305	10.884881
H	-2.433143	3.791243	11.173794
H	-4.134304	3.728244	11.700362
C	-1.965137	0.114965	8.756808
H	-2.646395	-0.207163	7.946217
H	-0.946473	-0.180738	8.451304
C	-4.196464	1.107087	4.837727
H	-4.474771	0.094216	4.525868
C	-2.150389	-2.190035	9.810989
H	-2.777195	-2.563414	8.978708
H	-2.425287	-2.759720	10.717267
H	-1.096867	-2.424716	9.570836
C	3.201614	0.429993	8.955541
H	4.066335	-0.218936	8.725669
H	2.650035	-0.028061	9.797335
H	3.597502	1.403571	9.302496
C	-5.374121	3.819010	9.255037
H	-5.674475	3.493695	8.242892
H	-5.565717	4.903925	9.321146
H	-6.031028	3.313331	9.989054
C	-3.815722	-0.340268	10.389523
H	-4.485485	-0.718530	9.588798
H	-4.110662	-0.864162	11.319590
C	1.079062	1.494897	8.048124
H	1.437304	2.499470	8.349917
H	0.559326	1.077025	8.928849
C	-4.478030	3.508532	4.722349
H	-4.957151	4.398625	4.303171
C	0.843131	3.737673	3.515982
H	1.783618	3.327127	3.102381
H	1.082876	4.648054	4.093619
H	0.214874	4.046488	2.661312
C	0.158761	4.770522	9.646081
C	0.396031	6.261471	9.476237
H	-0.545328	4.506308	10.450202
H	1.066111	4.147652	9.681445
C	-0.406551	7.174124	10.422815
H	1.476436	6.506636	9.475169
H	0.098746	6.576691	8.401418
H	-1.453555	6.822284	10.461842
C	-0.386533	8.650185	10.005122
H	-0.008008	7.075681	11.448524
H	-0.858836	8.788754	9.010910
C	-1.071376	9.579804	11.015378
H	0.657396	9.013310	9.883438
O	-1.386566	9.193815	12.137683
C	-1.342396	11.005642	10.545881
H	-2.156536	10.998096	9.795564
H	-1.647744	11.632191	11.397898
H	-0.456964	11.443746	10.049178

Alternating pathway, path A

TS-1$_{\mathbf{Coor\text{-}CO\text{-}T}}$
SCF done: -2452.61126 A.U.

Pd	-0.482579	4.882632	8.529326
S	-2.660751	5.893723	6.630001
P	-1.243382	2.854681	7.786700
O	-1.114264	5.740528	6.640696
C	-3.913464	1.015193	10.646155
H	-3.375714	1.278475	11.578555
H	-4.986279	1.197031	10.847048
C	0.833627	2.476867	5.691757
H	1.276516	3.436707	6.020963
C	-2.648707	2.997965	6.567031
C	-1.952430	1.688020	9.163131
H	-1.335984	1.954301	10.045693
O	-3.218599	6.137759	7.994951
C	-0.094708	2.817684	4.483536
H	-0.975158	3.343044	4.888179
O	-3.093329	6.801866	5.539894
C	-3.456439	1.964807	9.506664
H	-4.029307	1.679881	8.600236
C	0.155716	1.854432	6.959588
H	-0.279320	0.871379	6.685887
C	-3.183616	1.795253	6.044619
H	-2.738237	0.836341	6.325875
C	2.399379	0.728452	7.626648
H	1.974909	-0.267472	7.373586
C	-0.594430	1.581879	3.707326
H	-1.377013	1.877044	2.985687
H	-1.033678	0.812346	4.368710
H	0.221421	1.104441	3.133454
C	3.048909	1.306877	6.357489
H	3.842978	0.628785	5.988319
H	3.546203	2.265777	6.613544
C	-4.830907	3.007318	4.742127
H	-5.670249	3.020730	4.037862
C	-3.227064	4.219788	6.131102
C	-2.194393	-0.737337	10.038923
H	-1.600055	-0.459867	10.936372
C	-3.828797	3.450995	9.823720
H	-3.200791	4.115843	9.200814
C	2.000434	1.543961	5.259777
H	1.584470	0.564414	4.948490
H	2.488014	1.971840	4.364546
C	-3.593323	3.870199	11.291390
H	-3.658093	4.969411	11.377797
H	-2.601331	3.563242	11.671975
H	-4.352345	3.437285	11.969389
C	-1.747024	0.174137	8.872310
H	-2.336436	-0.119276	7.981555

H	-0.692747	-0.051159	8.638526
C	-4.262486	1.791925	5.148937
H	-4.648021	0.839635	4.768053
C	-1.920130	-2.216895	9.716303
H	-2.486724	-2.538453	8.821535
H	-2.224143	-2.868689	10.555516
H	-0.847620	-2.399309	9.518601
C	3.413962	0.544654	8.768707
H	4.242464	-0.119285	8.461287
H	2.941685	0.102378	9.665560
H	3.855207	1.515705	9.064091
C	-5.289731	3.745040	9.424861
H	-5.460126	3.539280	8.353196
H	-5.528147	4.808566	9.599019
H	-6.004866	3.134640	10.010379
C	-3.677239	-0.472894	10.350409
H	-4.291036	-0.790976	9.481537
H	-4.005194	-1.090055	11.209562
C	1.240128	1.652476	8.059165
H	1.660000	2.647772	8.308481
H	0.798331	1.265629	8.996724
C	-4.303577	4.211956	5.225588
H	-4.698469	5.176811	4.892522
C	0.585974	3.820723	3.527027
H	1.490179	3.398676	3.049008
H	0.872015	4.743131	4.061776
H	-0.108919	4.107044	2.716960
C	0.131660	4.220491	10.376645
H	-0.794191	4.168651	10.976861
H	0.618582	3.231958	10.375743
C	1.081565	5.296496	10.936987
H	1.291242	5.175449	12.020351
H	2.063956	5.231360	10.420149
C	0.566710	6.679505	10.621336
O	-0.114265	6.846973	9.589061
C	0.892742	7.850435	11.515967
H	0.402356	7.711174	12.498616
H	1.980870	7.895993	11.709529
H	0.547862	8.792593	11.064439
C	1.812230	5.531382	7.709124
O	2.030892	5.875941	6.625812

1-coor-CO-T

SCF done: -2452.622799 A.U.

Pd	-1.092166	5.104165	7.894773
S	-3.769943	5.007308	6.313239
P	-1.504268	2.732509	7.594550
O	-2.309279	5.518123	6.120083
C	-3.957580	1.285471	10.862119
H	-3.236189	1.207861	11.699460
H	-4.889401	1.692936	11.294334
C	1.016019	2.814171	6.173510
H	1.284493	3.670539	6.830102
C	-2.694286	2.364291	6.192777
C	-2.169432	1.703276	9.081192
H	-1.315719	1.708940	9.789130
O	-4.133843	4.911299	7.759865
C	0.323917	3.432230	4.916080
H	-0.677992	3.786283	5.226558
O	-4.700952	5.718999	5.406287
C	-3.414648	2.306534	9.822268
H	-4.201433	2.473210	9.059259
C	0.121733	1.874729	7.051144
H	-0.168609	0.998671	6.439841
C	-2.666987	1.092144	5.571317
H	-1.943132	0.338469	5.892762
C	2.220519	0.614225	7.871304
H	1.932909	-0.251367	7.235642
C	0.130282	2.442692	3.748599
H	-0.501444	2.900575	2.966855
H	-0.368355	1.508467	4.063274
H	1.092949	2.171670	3.277093
C	3.101223	1.547750	7.023745
H	4.016183	1.017355	6.695859
H	3.440068	2.396459	7.655306
C	-4.499816	1.679810	4.091256
H	-5.197488	1.422614	3.286626
C	-3.646438	3.297262	5.702882
C	-2.904169	-0.706841	9.740316
H	-2.140614	-0.728012	10.547746
C	-3.158749	3.686374	10.515040
H	-2.647272	4.327939	9.770051
C	2.329984	2.077453	5.804437
H	2.094864	1.223605	5.137766
H	2.973482	2.758877	5.217760
C	-2.267886	3.606659	11.774987
H	-1.952728	4.620290	12.081641
H	-1.348709	3.010319	11.622707
H	-2.810719	3.166251	12.631515
C	-2.420668	0.241676	8.622528
H	-3.200985	0.244235	7.838268
H	-1.508701	-0.179574	8.156082
C	-3.557395	0.746915	4.542846
H	-3.504640	-0.253080	4.098190
C	-3.083931	-2.137312	9.202475
H	-3.838389	-2.162941	8.393254
H	-3.427088	-2.819856	10.001070
H	-2.138667	-2.542328	8.795422
C	2.959124	0.075215	9.108616
H	3.875025	-0.470280	8.817349
H	2.324566	-0.617285	9.691814
H	3.262499	0.900379	9.781081
C	-4.482579	4.404815	10.852276
H	-5.098332	4.537849	9.948182
H	-4.274547	5.408548	11.266074
H	-5.069415	3.855358	11.612518
C	-4.203202	-0.131081	10.325915
H	-4.983199	-0.118839	9.536267
H	-4.583975	-0.780798	11.137861
C	0.923101	1.352615	8.274045
H	1.198029	2.206594	8.925543
H	0.308058	0.671701	8.888930
C	-4.532510	2.956174	4.667615
H	-5.232368	3.722776	4.321318
C	1.075680	4.690860	4.431161
H	2.092961	4.454253	4.065883

H	1.165205	5.438449	5.240753
H	0.528173	5.168085	3.599048
C	0.143571	4.841480	9.577974
H	-0.361652	5.435321	10.358541
H	0.107542	3.782907	9.880470
C	1.592049	5.322637	9.391302
H	2.128224	4.688966	8.660665
H	1.617804	6.364007	9.027197
C	2.315738	5.308276	10.756561
O	2.208192	6.249828	11.534695
C	3.134052	4.066245	11.092962
H	4.007868	3.999025	10.416239
H	3.483755	4.109199	12.135860
H	2.537707	3.147897	10.933465
C	-0.792883	7.012717	7.982804
O	-0.692391	8.162949	7.951699

TS-1$_{Ins\text{-}CO\text{-}T}$

SCF done: -2452.598277 A.U.

Pd	-1.202019	4.957074	8.113040
S	-3.818371	4.914805	6.453277
P	-1.600015	2.563348	7.621568
O	-2.364327	5.502235	6.423218
C	-4.092181	0.995733	10.785728
H	-3.358942	0.846380	11.603505
H	-5.003627	1.405365	11.257566
C	0.977639	2.762812	6.361200
H	1.165474	3.595898	7.076255
C	-2.710343	2.291725	6.143197
C	-2.318561	1.443921	9.007178
H	-1.470802	1.370872	9.717768
O	-4.280385	4.658827	7.849238
C	0.351300	3.418051	5.089523
H	-0.683603	3.715799	5.344341
O	-4.690409	5.709573	5.556954
C	-3.533436	2.049603	9.788683
H	-4.326493	2.277687	9.048910
C	0.060403	1.749489	7.128359
H	-0.159699	0.894971	6.458839
C	-2.654486	1.075314	5.421881
H	-1.958565	0.290439	5.732792
C	2.167260	0.569193	8.075617
H	1.996339	-0.298079	7.400945
C	0.282797	2.478408	3.868468
H	-0.306482	2.948139	3.060907
H	-0.199987	1.513536	4.105908
H	1.288722	2.264671	3.461980
C	3.051577	1.578922	7.323769
H	4.019588	1.112167	7.055863
H	3.285979	2.424506	8.003336
C	-4.372685	1.815617	3.875514
H	-5.015300	1.638779	3.005875
C	-3.620590	3.277135	5.675160
C	-3.134302	-0.969023	9.520962
H	-2.361278	-1.075588	10.312644
C	-3.219643	3.390569	10.530945
H	-2.766249	4.068298	9.776117
C	2.344850	2.097623	6.062002
H	2.192014	1.248404	5.365792
H	2.994216	2.819206	5.531672
C	-2.225276	3.254193	11.703878
H	-1.950654	4.255735	12.083504
H	-1.286420	2.741114	11.422609
H	-2.663812	2.700564	12.554440
C	-2.615679	0.024210	8.459627
H	-3.383798	0.096355	7.665427
H	-1.708340	-0.395305	7.980877
C	-3.475481	0.830980	4.310219
H	-3.405687	-0.129479	3.787321
C	-3.387322	-2.354719	8.901691
H	-4.158087	-2.298970	8.109545
H	-3.743093	-3.071043	9.664603
H	-2.468571	-2.772315	8.449225
C	2.836507	0.062338	9.365765
H	3.813550	-0.406438	9.146532
H	2.209470	-0.691056	9.877737
H	3.006668	0.896893	10.070744
C	-4.507793	4.098981	10.998861
H	-5.196487	4.256508	10.152562
H	-4.264882	5.091717	11.420615
H	-5.033098	3.529502	11.788612
C	-4.397564	-0.375722	10.166397
H	-5.189117	-0.280541	9.393932
H	-4.792935	-1.060441	10.941999
C	0.791336	1.204063	8.385933
H	0.951882	2.023969	9.117006
H	0.171386	0.442582	8.892814
C	-4.435393	3.039448	4.555428
H	-5.108304	3.838758	4.230558
C	1.077866	4.729442	4.720309
H	2.136621	4.559159	4.447937
H	1.046052	5.449395	5.559350
H	0.586981	5.211430	3.856078
C	0.304779	5.245834	9.867704
H	-0.126343	5.613666	10.810770
H	0.190726	4.147725	9.871509
C	1.780175	5.599914	9.688855
H	2.081072	5.522091	8.622819
H	1.985561	6.647174	9.977440
C	2.715192	4.639719	10.449211
O	2.362793	3.502066	10.753566
C	4.103395	5.171855	10.770535
H	4.027274	5.949140	11.555329
H	4.750674	4.357836	11.130709
H	4.557902	5.658159	9.887445
C	-0.551164	6.583894	8.781938
O	-0.326022	7.723170	8.976687

1-cycle-6-C

SCF done: -2452.624625 A.U.

Pd	-0.416544	5.025336	7.619226
S	-3.290310	5.138765	6.436013
P	-1.378300	2.705090	7.912949
O	-1.790288	5.395198	6.059213
C	-3.692421	2.408681	11.549789
H	-2.925590	2.295161	12.342549

H	-4.457449	3.095240	11.956106
C	0.959961	2.086160	6.356165
H	1.376641	3.020438	6.795833
C	-2.733596	2.337930	6.677263
C	-2.078143	2.062495	9.589447
H	-1.167146	1.983709	10.215573
O	-3.541615	5.306341	7.899526
C	0.257775	2.509971	5.026448
H	-0.656526	3.069010	5.302521
O	-4.167068	5.874574	5.493295
C	-3.046884	3.067700	10.298214
H	-3.857363	3.309089	9.581636
C	0.024204	1.481403	7.458551
H	-0.422785	0.543141	7.073652
C	-3.002975	1.009245	6.271431
H	-2.413796	0.186060	6.686652
C	2.052622	0.208199	8.457340
H	1.652236	-0.750867	8.062416
C	-0.163621	1.324280	4.134534
H	-0.798664	1.682383	3.304643
H	-0.745605	0.564649	4.686399
H	0.712240	0.820469	3.684907
C	2.956095	0.818243	7.372180
H	3.792340	0.129488	7.141354
H	3.413871	1.751531	7.762830
C	-4.775866	1.731821	4.779359
H	-5.562265	1.505820	4.050481
C	-3.515363	3.362717	6.079285
C	-3.258273	0.054510	10.750095
H	-2.436274	-0.018662	11.495083
C	-2.376970	4.430690	10.676771
H	-1.899438	4.805530	9.748279
C	2.150343	1.126480	6.101107
H	1.771539	0.172586	5.681673
H	2.812867	1.562291	5.330811
C	-1.285021	4.322986	11.762974
H	-0.792267	5.305113	11.900080
H	-0.497324	3.588862	11.511205
H	-1.702872	4.045803	12.747978
C	-2.689542	0.646378	9.442532
H	-3.519677	0.684531	8.711464
H	-1.934614	-0.048746	9.023322
C	-4.011387	0.702487	5.344740
H	-4.190162	-0.342069	5.065373
C	-3.826940	-1.355941	10.515000
H	-4.646937	-1.332035	9.772121
H	-4.234850	-1.781512	11.450242
H	-3.052325	-2.049489	10.138099
C	2.824175	-0.092173	9.754559
H	3.667340	-0.781662	9.565161
H	2.173206	-0.556726	10.518318
H	3.242972	0.835989	10.188639
C	-3.425389	5.489948	11.076235
H	-4.170295	5.621157	10.274411
H	-2.939388	6.470565	11.242350
H	-3.948500	5.227927	12.015089
C	-4.314269	1.027050	11.300276
H	-5.147412	1.107103	10.571040
H	-4.751433	0.634534	12.239307
C	0.848907	1.139729	8.729944
H	1.224564	2.081356	9.183498
H	0.204899	0.656085	9.486962
C	-4.518409	3.060584	5.142795
H	-5.078878	3.891658	4.704194
C	1.123975	3.504659	4.223223
H	2.083413	3.056754	3.901163
H	1.340086	4.414507	4.810920
H	0.588043	3.824992	3.311684
C	0.447813	6.752031	7.122596
O	0.811197	6.991539	5.989850
C	0.702664	7.755024	8.271957
H	1.710907	8.188397	8.132023
H	-0.026759	8.572477	8.122200
C	0.529126	7.186933	9.699817
H	0.824245	7.941232	10.449256
H	-0.539750	6.940690	9.859324
C	1.304580	5.898853	9.896597
O	1.080534	4.905731	9.171255
C	2.362155	5.815813	10.965190
H	3.132995	6.590486	10.788182
H	1.913864	6.039832	11.951821
H	2.830383	4.820583	10.983594

TS-2$_{Coor\text{-}E\text{-}C}$

SCF done: -2531.16993170 A.U.

Pd	-1.644627	-0.792801	-0.571085
S	-1.836200	-0.082952	2.471909
P	0.742911	0.102314	0.044085
O	-1.638805	-1.328533	1.564231
C	1.557906	4.240704	-0.985795
H	1.091321	4.222470	-1.990754
H	1.235332	5.186723	-0.510871
C	1.822722	-2.639593	0.315201
H	0.859549	-2.992707	-0.106175
C	0.931550	0.505813	1.870033
C	1.489445	1.700995	-0.796387
H	1.064661	1.634475	-1.820279
O	-2.525263	1.047571	1.759257
C	1.652788	-2.714496	1.865291
H	0.962057	-1.905705	2.154741
O	-2.411020	-0.472379	3.784742
C	1.014516	3.047407	-0.152952
H	1.504163	3.093661	0.842111
C	2.072745	-1.225879	-0.312957
H	3.047482	-0.842048	0.051932
C	2.205606	0.891138	2.352087
H	3.062463	0.874794	1.673374
C	3.253422	-2.321375	-2.344804
H	4.224015	-1.913187	-1.987267
C	2.965007	-2.529285	2.655208
H	2.748396	-2.425930	3.733511
H	3.522365	-1.626149	2.346459
H	3.638924	-3.398028	2.536691
C	3.057618	-3.705346	-1.701290
H	3.894419	-4.377426	-1.975449
H	2.138515	-4.168216	-2.119034

C	1.354295	1.273722	4.589653
H	1.506040	1.568460	5.634074
C	-0.128091	0.478990	2.818085
C	3.555561	2.910138	-1.783971
H	3.099287	2.822018	-2.793870
C	-0.519276	3.191505	0.104038
H	-0.914270	2.208299	0.421546
C	2.941663	-3.600167	-0.172102
H	3.913434	-3.252288	0.232790
H	2.770796	-4.604659	0.257487
C	-1.318553	3.619889	-1.144423
H	-2.401487	3.577042	-0.925193
H	-1.117986	2.965863	-2.012220
H	-1.093162	4.660661	-1.441244
C	3.039156	1.735224	-0.920794
H	3.487272	1.853088	0.084632
H	3.439905	0.793775	-1.331879
C	2.422485	1.277693	3.682931
H	3.427851	1.571615	4.005232
C	5.085671	2.853487	-1.937508
H	5.584601	2.930764	-0.952467
H	5.454307	3.686035	-2.564439
H	5.412566	1.906954	-2.406381
C	3.301464	-2.397390	-3.881119
H	4.112761	-3.066601	-4.221966
H	3.472603	-1.402345	-4.332227
H	2.350724	-2.792111	-4.288094
C	-0.795293	4.157767	1.274447
H	-0.289747	3.818001	2.195912
H	-1.877322	4.204348	1.490876
H	-0.450790	5.186141	1.051516
C	3.083580	4.235048	-1.160072
H	3.577748	4.363591	-0.174046
H	3.400316	5.092746	-1.785407
C	2.135251	-1.371069	-1.863367
H	1.159291	-1.758391	-2.214347
H	2.258362	-0.390510	-2.357220
C	0.088801	0.858557	4.155736
H	-0.753629	0.794050	4.850999
C	0.953396	-4.025853	2.285267
H	1.558809	-4.921263	2.047889
H	-0.028744	-4.126154	1.790158
H	0.774512	-4.031334	3.375753
C	-3.523521	-1.407301	-0.535832
O	-3.833835	-2.579577	-0.499719
C	-4.592605	-0.298881	-0.456404
H	-5.473004	-0.660953	-1.020236
H	-4.872232	-0.262479	0.613071
C	-4.179931	1.123799	-0.886823
H	-5.073977	1.771129	-0.912067
H	-3.487036	1.506185	-0.108995
C	-3.442228	1.169304	-2.207518
O	-2.372954	0.550250	-2.361249
C	-4.012696	1.988543	-3.342717
H	-5.027414	1.624658	-3.594608
H	-4.128120	3.040896	-3.021133
H	-3.365636	1.938620	-4.231364
C	-1.226312	-3.181781	-2.049665
H	-2.001419	-3.685567	-1.464947
H	-0.190521	-3.485334	-1.867164
C	-1.533665	-2.260368	-2.991181
H	-0.766825	-1.760007	-3.593540
H	-2.573019	-2.010586	-3.227651

2-Coor-E-C

SCF done: -2531.193287 A.U.

Pd	-0.487507	5.040865	8.703392
S	-3.244660	5.533260	7.240794
P	-1.331862	2.752485	7.951477
O	-1.725781	5.868522	7.106527
C	-3.985891	1.077659	10.941014
H	-3.287062	0.713290	11.720325
H	-4.835647	1.537437	11.477967
C	1.159541	2.718978	6.504423
H	1.514761	3.417209	7.294119
C	-2.569938	2.848585	6.540748
C	-2.166855	1.536975	9.198152
H	-1.323133	1.254336	9.859609
O	-3.625997	5.216153	8.650524
C	0.571322	3.617352	5.367959
H	-0.380649	4.041471	5.742255
O	-4.050703	6.558428	6.527117
C	-3.277438	2.177336	10.100159
H	-4.030517	2.634428	9.427038
C	0.135454	1.765131	7.207773
H	-0.264581	1.061409	6.452137
C	-2.741439	1.747790	5.667367
H	-2.132760	0.848883	5.799066
C	2.062757	0.132673	7.798400
H	1.704578	-0.574499	7.018805
C	0.266327	2.858389	4.059876
H	-0.296504	3.508973	3.366854
H	-0.345078	1.953075	4.225177
H	1.193755	2.550805	3.542407
C	3.062580	1.094221	7.135305
H	3.924210	0.529006	6.729643
H	3.472991	1.780204	7.906521
C	-4.477755	2.889684	4.411909
H	-5.212657	2.910892	3.599587
C	-3.377791	3.990006	6.286994
C	-3.293387	-0.797749	9.404535
H	-2.538411	-1.103468	10.160957
C	-2.758910	3.322541	11.031881
H	-2.187652	4.016539	10.383154
C	2.385778	1.904238	6.018533
H	2.067511	1.204422	5.219464
H	3.117489	2.591462	5.555289
C	-1.829227	2.838594	12.167840
H	-1.326571	3.699157	12.646859
H	-1.038941	2.145584	11.820850
H	-2.392821	2.317341	12.963357
C	-2.662515	0.259541	8.473710
H	-3.432024	0.543878	7.731166
H	-1.833011	-0.203294	7.903584
C	-3.680428	1.757715	4.624092
H	-3.780330	0.876793	3.980034
C	-3.719961	-2.045605	8.611570

H	-4.473721	-1.786463	7.843806
H	-4.168352	-2.805342	9.277546
H	-2.860913	-2.513711	8.095655
C	2.704077	-0.686353	8.932398
H	3.562191	-1.275057	8.559995
H	1.981911	-1.391088	9.384637
H	3.077483	-0.024749	9.737382
C	-3.919275	4.152094	11.620886
H	-4.556852	4.558703	10.819028
H	-3.522658	5.007414	12.199150
H	-4.545327	3.557321	12.312151
C	-4.470797	-0.141531	10.143758
H	-5.239080	0.161681	9.402060
H	-4.956209	-0.868170	10.824280
C	0.839525	0.926413	8.311077
H	1.184096	1.597824	9.124634
H	0.130799	0.213756	8.770808
C	-4.314891	4.007688	5.240381
H	-4.896475	4.921501	5.085902
C	1.485239	4.829013	5.083974
H	2.472051	4.521370	4.688754
H	1.646703	5.432875	5.994787
H	1.020888	5.488374	4.328531
C	0.927933	4.202558	10.136446
H	0.468942	3.296090	10.542213
H	1.887205	4.070669	9.621386
C	0.568150	5.456576	10.647054
C	0.018193	7.023758	8.567156
O	1.021131	7.317851	7.959988
H	1.255705	6.303178	10.555866
H	-0.221433	5.553986	11.400420
C	-1.033961	7.977336	9.109913
H	-1.891744	7.405750	9.498613
H	-0.577059	8.516232	9.965303
C	-1.489322	9.006979	8.060805
H	-0.614466	9.384460	7.492502
H	-2.158208	8.532224	7.320598
C	-2.171260	10.216402	8.710413
O	-2.035864	10.475194	9.904181
C	-3.024037	11.074005	7.785124
H	-2.484270	11.311816	6.849742
H	-3.927181	10.504889	7.492377
H	-3.326216	12.001124	8.295834

TS-2$_{Ins-E-C}$

SCF done: -2531.185482 A.U.

Pd	-0.509275	4.912070	8.049373
S	-3.153262	5.383593	6.393397
P	-1.295343	2.723234	7.652111
O	-1.635633	5.690049	6.334647
C	-4.037478	1.592845	10.812811
H	-3.357242	1.400414	11.666210
H	-4.911742	2.125383	11.229117
C	1.244772	2.463981	6.314614
H	1.585069	3.279588	6.989255
C	-2.483819	2.596032	6.212516
C	-2.163193	1.765549	9.079776
H	-1.339854	1.631940	9.810465
O	-3.652490	5.316437	7.806633
C	0.694833	3.169612	5.033516
H	-0.259556	3.660117	5.305930
O	-3.910913	6.245615	5.452326
C	-3.323022	2.534824	9.800531
H	-4.059320	2.825769	9.024429
C	0.201213	1.641048	7.144918
H	-0.174397	0.808872	6.517179
C	-2.636892	1.355867	5.545729
H	-2.047568	0.490599	5.861590
C	2.123906	0.177549	8.089934
H	1.800506	-0.679050	7.459439
C	0.417622	2.210098	3.858088
H	-0.095177	2.750060	3.042220
H	-0.231244	1.363594	4.146364
H	1.354160	1.795052	3.441456
C	3.132063	1.004218	7.272491
H	4.008467	0.381596	7.006787
H	3.515287	1.835136	7.902237
C	-4.300168	2.273553	4.036398
H	-5.000045	2.157771	3.201332
C	-3.259723	3.685237	5.733204
C	-3.264058	-0.517437	9.672755
H	-2.528562	-0.665945	10.492943
C	-2.878937	3.852988	10.517308
H	-2.286928	4.428005	9.776842
C	2.475271	1.573533	6.006435
H	2.167448	0.733529	5.351777
H	3.213436	2.160905	5.429607
C	-1.995623	3.626465	11.763761
H	-1.560470	4.586031	12.100843
H	-1.155602	2.930069	11.579431
H	-2.575854	3.225577	12.615365
C	-2.617822	0.366440	8.584140
H	-3.367620	0.495789	7.780388
H	-1.764815	-0.177201	8.132071
C	-3.533316	1.188055	4.479325
H	-3.621885	0.207892	3.997372
C	-3.643829	-1.896819	9.106560
H	-4.375097	-1.798064	8.281823
H	-4.102823	-2.532084	9.886054
H	-2.760709	-2.432863	8.711677
C	2.742946	-0.383352	9.382227
H	3.622129	-1.015190	9.159828
H	2.018688	-0.999516	9.946527
H	3.078729	0.435012	10.047478
C	-4.089715	4.737821	10.879787
H	-4.682675	4.974881	9.981584
H	-3.745803	5.693623	11.317692
H	-4.745890	4.258368	11.630318
C	-4.474862	0.235546	10.245946
H	-5.227519	0.376933	9.442387
H	-4.965363	-0.363953	11.037632
C	0.870526	1.027804	8.403809
H	1.161471	1.842128	9.098093
H	0.152108	0.392936	8.952176
C	-4.151710	3.519367	4.659611
H	-4.709220	4.397521	4.320175

C	1.629921	4.313546	4.583893
H	2.624882	3.942918	4.272698
H	1.772759	5.053315	5.392461
H	1.191772	4.848353	3.722557
C	0.820900	4.660368	9.609925
H	0.313453	4.464290	10.563125
H	1.599564	3.926670	9.362855
C	1.089666	6.054184	9.244752
C	-0.188282	7.142135	8.373294
O	0.303563	7.824600	7.502712
H	1.957500	6.232790	8.597222
H	1.001724	6.797610	10.058578
C	-1.405991	7.517140	9.213965
H	-2.290572	7.087055	8.702969
H	-1.338297	7.029221	10.200421
C	-1.556733	9.037993	9.360446
H	-1.535828	9.524834	8.366585
H	-2.547008	9.282440	9.793639
C	-0.471387	9.661960	10.239694
O	0.398513	8.977006	10.779283
C	-0.513242	11.174455	10.409426
H	-0.296435	11.662402	9.439913
H	-1.519109	11.514315	10.717929
H	0.235707	11.492930	11.150341

2-cycle-5-T

SCF done: -2531.2131 A.U.

Pd	-1.137189	4.925188	8.351432
S	-3.738358	5.215086	6.672876
P	-1.542790	2.740128	7.816561
O	-2.253589	5.672241	6.669976
C	-4.078169	0.911202	10.820926
H	-3.369995	0.815360	11.667844
H	-5.040413	1.233774	11.258075
C	0.994492	2.932177	6.438991
H	1.240660	3.732384	7.171125
C	-2.710890	2.547507	6.362436
C	-2.252667	1.557709	9.160180
H	-1.442122	1.533000	9.917079
O	-4.274300	5.022895	8.053354
C	0.327896	3.647625	5.219969
H	-0.679064	3.979533	5.534936
O	-4.541074	6.033044	5.730172
C	-3.569297	2.032518	9.869357
H	-4.328275	2.178537	9.073370
C	0.085872	1.923776	7.222033
H	-0.195384	1.099376	6.537894
C	-2.697715	1.334286	5.629790
H	-1.990664	0.543029	5.893581
C	2.182451	0.609976	7.984280
H	1.921062	-0.212254	7.283344
C	0.163355	2.749729	3.976009
H	-0.461127	3.260366	3.221458
H	-0.329345	1.788943	4.210090
H	1.136757	2.525087	3.501314
C	3.070072	1.612188	7.225654
H	3.996428	1.115499	6.876764
H	3.387067	2.414495	7.925370
C	-4.483205	2.091949	4.172398
H	-5.168603	1.924079	3.334079
C	-3.625099	3.547587	5.934694
C	-2.889097	-0.931107	9.584022
H	-2.143859	-0.981427	10.407341
C	-3.466772	3.384820	10.649165
H	-2.967503	4.108285	9.975586
C	2.315219	2.225677	6.036581
H	2.088767	1.421265	5.308337
H	2.966402	2.946354	5.507912
C	-2.650307	3.293674	11.957424
H	-2.455611	4.308341	12.351944
H	-1.670662	2.796743	11.825115
H	-3.197817	2.745620	12.746459
C	-2.428756	0.133067	8.564953
H	-3.196786	0.175775	7.769316
H	-1.493636	-0.208441	8.080468
C	-3.572091	1.100171	4.557762
H	-3.529003	0.143192	4.025648
C	-2.980279	-2.319322	8.926315
H	-3.712639	-2.316514	8.096645
H	-3.306101	-3.082326	9.656742
H	-2.005480	-2.640970	8.514544
C	2.902855	-0.001622	9.198578
H	3.832970	-0.514044	8.892055
H	2.266063	-0.739803	9.720122
H	3.177836	0.781913	9.930435
C	-4.863758	3.972597	10.940002
H	-5.428656	4.118583	10.005267
H	-4.765684	4.962167	11.422719
H	-5.452711	3.332388	11.624305
C	-4.230113	-0.474467	10.179953
H	-4.993517	-0.447592	9.374461
H	-4.588930	-1.203357	10.932842
C	0.870699	1.307288	8.412374
H	1.133272	2.109163	9.130252
H	0.247092	0.582650	8.966027
C	-4.495009	3.314813	4.855178
H	-5.163064	4.128648	4.556941
C	1.085644	4.941567	4.851346
H	2.119961	4.740728	4.512799
H	1.128576	5.632472	5.713148
H	0.565555	5.469105	4.032006
C	0.032243	4.603411	10.014132
H	-0.667649	4.744448	10.857142
H	0.513627	3.620981	10.131850
C	1.090933	5.728264	10.002963
H	1.630856	5.837273	10.967741
H	1.872149	5.516893	9.240823
C	0.449735	7.027773	9.585548
O	-0.542272	6.978798	8.822813
C	0.971784	8.350874	10.086500
H	0.751513	8.411754	11.176021
H	2.078386	8.338838	10.034041
C	0.370753	9.585831	9.408940
H	-0.658773	9.356130	9.065354
H	0.921554	9.867583	8.491463
C	0.283786	10.788099	10.357556

O	0.462232	10.667750	11.567635
C	-0.055361	12.127362	9.718834
H	0.801737	12.479499	9.112868
H	-0.914380	12.033287	9.029318
H	-0.275165	12.873208	10.497636

Alternating pathway, path B

1cycle-6-T

SCF done: -2452.640055 A.U.

Pd	-0.554277	5.007841	7.859698
S	-3.088685	5.416543	6.041773
P	-1.246002	2.818515	7.561983
O	-1.555860	5.639068	6.025084
C	-3.812729	1.781120	10.893383
H	-3.060413	1.792315	11.706665
H	-4.722153	2.254154	11.307108
C	1.240613	2.504477	6.108353
H	1.648427	3.329254	6.733832
C	-2.485221	2.599355	6.175008
C	-2.030824	1.962261	9.088893
H	-1.200594	2.002669	9.822485
O	-3.659530	5.540504	7.416724
C	0.621172	3.181104	4.843844
H	-0.296991	3.710782	5.160620
O	-3.752292	6.176897	4.953157
C	-3.288960	2.659916	9.720437
H	-4.070882	2.676057	8.932405
C	0.244788	1.731491	7.039712
H	-0.176056	0.879826	6.469404
C	-2.690274	1.293594	5.662873
H	-2.105200	0.455894	6.051877
C	2.176304	0.234446	7.879024
H	1.761614	-0.619256	7.299657
C	0.234934	2.192134	3.724731
H	-0.334447	2.717270	2.937167
H	-0.402558	1.367928	4.091755
H	1.126658	1.746146	3.246027
C	3.151544	0.994021	6.962827
H	3.973569	0.325807	6.639288
H	3.622885	1.816542	7.541260
C	-4.382515	2.074686	4.110894
H	-5.114781	1.882241	3.318716
C	-3.243023	3.649406	5.590466
C	-2.842580	-0.349483	9.958371
H	-2.050152	-0.324852	10.737106
C	-3.109500	4.136858	10.205516
H	-2.549579	4.674679	9.415042
C	2.421736	1.568929	5.739288
H	2.041103	0.728258	5.124949
H	3.134813	2.120835	5.099028
C	-2.327981	4.282848	11.528882
H	-2.085137	5.349124	11.698229
H	-1.376486	3.725143	11.525556
H	-2.925766	3.950078	12.397874
C	-2.366137	0.482699	8.747759
H	-3.182852	0.471352	8.000263
H	-1.502758	-0.024170	8.277085
C	-3.628764	1.026646	4.654762
H	-3.757048	-0.000428	4.294967
C	-3.085754	-1.815157	9.557884
H	-3.863004	-1.886918	8.773222
H	-3.429407	-2.410434	10.423614
H	-2.166427	-2.288982	9.166091
C	2.873352	-0.326670	9.130994
H	3.702834	-1.003229	8.854612
H	2.170091	-0.895688	9.766594
H	3.295818	0.489766	9.746926
C	-4.474843	4.848300	10.316766
H	-4.999735	4.850935	9.347932
H	-4.330427	5.903151	10.614047
H	-5.124841	4.379522	11.080690
C	-4.101918	0.317654	10.533766
H	-4.916701	0.259611	9.781879
H	-4.457104	-0.230523	11.428460
C	1.003624	1.163069	8.270519
H	1.402433	1.996963	8.876330
H	0.319361	0.605938	8.933968
C	-4.175500	3.380550	4.572933
H	-4.715846	4.229578	4.143057
C	1.552239	4.281260	4.289553
H	2.506402	3.871219	3.907477
H	1.785266	5.032758	5.066461
H	1.061211	4.812355	3.454885
C	0.662054	4.616355	9.369931
O	0.609889	3.753683	10.236559
C	1.832196	5.643771	9.390991
H	2.612717	5.232108	10.054761
H	2.251079	5.751406	8.373386
C	1.394936	7.022138	9.918816
H	0.905313	6.921026	10.910583
H	2.276282	7.669005	10.092850
C	0.428511	7.760409	9.007998
O	-0.200579	7.180790	8.108144
C	0.251284	9.248564	9.203692
H	0.131237	9.491119	10.275511
H	1.161283	9.775213	8.856270
H	-0.615373	9.609340	8.630107

TS-2$_{Ins\text{-}E\text{-}T}$

SCF done: -2531.16091117 A.U.

Pd	-0.484841	5.079836	7.648541
S	-2.902364	5.357014	5.799963
P	-1.096696	2.735580	7.395777
O	-1.339760	5.489134	5.749574
C	-3.441580	1.673130	10.871957
H	-2.644873	1.824792	11.626942
H	-4.365478	2.097382	11.306639
C	1.203002	2.295076	5.678698
H	1.639325	3.204541	6.144681
C	-2.448232	2.507536	6.116817
C	-1.781268	1.848519	8.961339
H	-0.946418	1.985058	9.679896
O	-3.447257	5.698873	7.146803
C	0.438813	2.790384	4.410524
H	-0.459336	3.332545	4.757186

O	-3.482277	6.050050	4.623410
C	-3.072739	2.474845	9.590946
H	-3.885449	2.316831	8.850057
C	0.336863	1.619888	6.796148
H	-0.102481	0.686652	6.389139
C	-2.764035	1.193116	5.692631
H	-2.179088	0.350648	6.070826
C	2.426461	0.335388	7.633041
H	2.007223	-0.613424	7.232026
C	-0.018036	1.656997	3.469366
H	-0.681136	2.060427	2.683220
H	-0.582226	0.868736	3.999258
H	0.840404	1.177335	2.963573
C	3.261357	0.989469	6.518311
H	4.074735	0.306597	6.203328
H	3.753400	1.902985	6.910826
C	-4.542334	1.982536	4.246842
H	-5.351162	1.791468	3.532870
C	-3.190642	3.567194	5.524133
C	-2.342777	-0.444246	10.033959
H	-1.505314	-0.299493	10.750245
C	-3.040937	4.007563	9.893993
H	-2.594054	4.511318	9.012421
C	2.380397	1.353517	5.313357
H	1.974739	0.422469	4.868061
H	2.997072	1.830449	4.529509
C	-2.201632	4.393461	11.130199
H	-2.171657	5.495839	11.226203
H	-1.158635	4.035736	11.065327
H	-2.644761	4.010762	12.068087
C	-2.013292	0.329131	8.737690
H	-2.874278	0.202415	8.053368
H	-1.143737	-0.142843	8.243707
C	-3.798420	0.924459	4.784156
H	-4.008853	-0.110993	4.493585
C	-2.490574	-1.950896	9.758563
H	-3.309472	-2.142735	9.039050
H	-2.725803	-2.503209	10.686791
H	-1.563360	-2.380422	9.335872
C	3.274609	-0.000963	8.872280

H	4.108593	-0.677607	8.610303
H	2.671892	-0.494167	9.657367
H	3.712362	0.915897	9.310346
C	-4.470603	4.575340	10.014921
H	-5.054954	4.381460	9.099212
H	-4.436902	5.670682	10.153333
H	-5.015713	4.148575	10.878707
C	-3.613380	0.162912	10.652617
H	-4.470668	-0.028565	9.973546
H	-3.853521	-0.335627	11.612167
C	1.237536	1.250160	8.009356
H	1.632769	2.176471	8.472182
H	0.648883	0.749021	8.797836
C	-4.221541	3.298007	4.604985
H	-4.746806	4.147012	4.157524
C	1.269935	3.832073	3.630143
H	2.199412	3.400302	3.213663
H	1.545317	4.685700	4.275523
H	0.683140	4.233201	2.784451
C	1.234315	5.014337	9.171170
O	1.126545	4.208737	10.093410
C	2.515751	5.138438	8.342081
H	2.620665	4.223987	7.729642
H	2.455228	5.987007	7.638922
C	3.759956	5.245164	9.245888
H	3.860474	6.251136	9.698638
H	3.643619	4.539426	10.094990
C	5.060629	4.865550	8.524102
O	5.059057	4.194092	7.496115
C	6.352274	5.350998	9.169267
H	6.440672	6.447392	9.042585
H	6.353795	5.155596	10.257682
H	7.218212	4.864093	8.695765
C	-0.193370	7.089863	8.074986
C	0.356682	6.515242	9.314168
H	-1.209704	7.503632	8.091716
H	0.489485	7.575122	7.363887
H	-0.343347	6.398522	10.151904
H	1.284682	7.021172	9.652934

6. References

(1) Ortmann, P.; Wimmer, F. P.; Mecking, S. Long-Spaced Polyketones from ADMET Copolymerizations as Ideal Models for Ethylene/CO Copolymers. *ACS Macro Lett.* **2015**, *4* (7), 704–707. https://doi.org/10.1021/acsmacrolett.5b00324.

(2) Drent, E.; van Dijk, R.; van Ginkel, R.; van Oort, B.; Pugh, R. I. The First Example of Palladium Catalysed Non-Perfectly Alternating Copolymerisation of Ethene and Carbon Monoxide. *Chem. Commun.* **2002**, No. 9, 964–965. https://doi.org/10.1039/b111629k.

(3) Skupov, K. M.; Marella, P. R.; Simard, M.; Yap, G. P. A.; Allen, N.; Conner, D.; Goodall, B. L.; Claverie, J. P. Palladium Aryl Sulfonate Phosphine Catalysts for the Copolymerization of Acrylates with Ethene. *Macromol. Rapid Commun.* **2007**, *28* (20), 2033–2038. https://doi.org/10.1002/marc.200700370.

(4) Neuwald, B.; Caporaso, L.; Cavallo, L.; Mecking, S. Concepts for Stereoselective Acrylate Insertion. *J. Am. Chem. Soc.* **2013**, *135* (3), 1026–1036. https://doi.org/10.1021/ja3101787.

(5) Allen, N. T.; Goodall, B. L.; McIntosh III, L. H.; Rohm and Haas Company. Substaintally Linear Polymers and Methods of Making and Using Them, July 17, 2006.

(6) Wucher, P.; Goldbach, V.; Mecking, S. Electronic Influences in Phosphinesulfonato Palladium(II) Polymerization Catalysts. *Organometallics* **2013**, *32* (16), 4516–4522. https://doi.org/10.1021/om400297x.

(7) Ota, Y.; Ito, S.; Kuroda, J.; Okumura, Y.; Nozaki, K. Quantification of the Steric Influence of Alkylphosphine-Sulfonate Ligands on Polymerization, Leading to High-Molecular-Weight Copolymers of Ethylene and Polar Monomers. *J. Am. Chem. Soc.* **2014**, *136* (34), 11898–11901. https://doi.org/10.1021/ja505558e.

(8) Newsham, D. K.; Borkar, S.; Sen, A.; Conner, D. M.; Goodall, B. L. Inhibitory Role of Carbon Monoxide in Palladium(II)-Catalyzed Nonalternating Ethene/Carbon Monoxide Copolymerizations and the Synthesis of Polyethene- Block-Poly(Ethene-Alt-Carbon Monoxide). *Organometallics* **2007**, *26* (15), 3636–3638. https://doi.org/10.1021/om700523m.

(9) Luo, R.; Newsham, D. K.; Sen, A. Palladium-Catalyzed Nonalternating Copolymerization of Ethene and Carbon Monoxide: Scope and Mechanism. *Organometallics* **2009**, *28* (24), 6994–7000. https://doi.org/10.1021/om9008235.

(10) Voccia, M.; Odenwald, L.; Baur, M.; Lin, F.; Falivene, L.; Mecking, S.; Caporaso, L. Mechanistic Insights into Ni(II)-Catalyzed Nonalternating Ethylene-Carbon Monoxide Copolymerization. *J. Am. Chem. Soc.* **2022**, *144* (33), 15111–15117. https://doi.org/10.1021/jacs.2c04563.

(11) Haras, A.; Michalak, A.; Rieger, B.; Ziegler, T. Theoretical Analysis of Factors Controlling the Nonalternating CO/C(2)H(4) Copolymerization. *J. Am. Chem. Soc.* **2005**, *127* (24), 8765–8774. https://doi.org/10.1021/ja050861d.

(12) Baur, M.; Lin, F.; Morgen, T. O.; Odenwald, L.; Mecking, S. Polyethylene Materials with In-Chain Ketones from Nonalternating Catalytic Copolymerization. *Science* **2021**, *374* (6567), 604–607. https://doi.org/10.1126/science.abi8183.

(13) Soomro, S. S.; Cozzula, D.; Leitner, W.; Vogt, H.; Müller, T. E. The Microstructure and Melt Properties of CO–Ethylene Copolymers with Remarkably Low CO Content. *Polym. Chem.* **2014**, *5* (12), 3831–3837. https://doi.org/10.1039/C3PY01637D.

(14) Pingen, D.; Klinkenberg, N.; Mecking, S. Single-Step Catalytic Upgrading of Microalgae Biomass. *ACS Sustainable Chem. Eng.* **2018**, *6* (9), 11219–11221. https://doi.org/10.1021/acssuschemeng.8b02939.

(15) Yonezaki, G.; Seidel, F. W.; Takahashi, K.; Nozaki, K. Nickel-Catalyzed Selective Incorporation of Isolated In-Chain Carbonyls into Ethylene/Carbon Monoxide Copolymer Using Metal Carbonyls as a Carbonyl Source. *BCSJ* **2023**, *96* (6), 545–549. https://doi.org/10.1246/bcsj.20230073.

(16) Tang, S.; Seidel, F. W.; Nozaki, K. High Density Polyethylenes Bearing Isolated In-Chain Carbonyls. *Angew. Chem. Int. Ed* **2021**, *60* (51), 26506–26510. https://doi.org/10.1002/anie.202110957.

(17) Baur, M.; Mecking, S. Polyethylenes with Combined In-Chain and Side-Chain Functional Groups from Catalytic Terpolymerization of Carbon Monoxide and Acrylate. *ACS Macro Lett.* **2022**, No. 11, 1207–1211. https://doi.org/10.1021/acsmacrolett.2c00459.
(18) Perdew, J. P. Density-Functional Approximation for the Correlation Energy of the Inhomogeneous Electron Gas. *Phys. Rev. B* **1986**, *33* (12), 8822–8824. https://doi.org/10.1103/PhysRevB.33.8822.
(19) Perdew, J. P. Erratum: Density-Functional Approximation for the Correlation Energy of the Inhomogeneous Electron Gas. *Phys. Rev. B* **1986**, *34* (10), 7406–7406. https://doi.org/10.1103/PhysRevB.34.7406.
(20) Becke, A. D. Density-Functional Exchange-Energy Approximation with Correct Asymptotic Behavior. *Phys. Rev. A* **1988**, *38* (6), 3098–3100. https://doi.org/10.1103/PhysRevA.38.3098.
(21) Frisch, M. J.; Trucks, G. W.; Schlegel, H. B.; Scuseria, G. E.; Robb, M. A.; Cheeseman, J.; Scalmani, G.; Barone, V.; Mennucci, B.; Petersson, G. A.; Nakatsuji, H.; Caricato, M.; Li, X.; Hratchian, H. P.; Izmaylov, A. F.; Bloino, J.; Zheng, G.; Sonnenberg, J.; Hada, M.; Ehara, M.; Toyota, K.; Fukuda, R.; Hasegawa, J.; Ishida, M.; Nakajima, T.; Honda, Y.; Kitao, O.; Nakai, H.; Vreven, T.; Montgomery, J. A.; Peralta, J. E.; Ogliaro, F.; Bearpark, M.; Heyd, J. J.; Brothers, E.; Kudin, K. N.; Staroverov, V. N.; Kobayashi, R.; Normand, J.; Raghavachari, K.; Rendell, A.; Burant, J. C.; Iyengar, S. S.; Tomasi, J.; Cossi, M.; Rega, N.; Millam, J. M.; Klene, M.; Knox, J. E.; Cross, J. B.; Bakken, V.; Adamo, C.; Jaramillo, J.; Gomperts, R.; Stratmann, R. E.; Yazyev, O.; Austin, A. J.; Cammi, R.; Pomelli, C.; Ochterski, J. W. R.; Martin, L.; Morokuma, K.; Zakrzewski, V. G.; Voth, G. A.; Salvador, P.; Dannenberg, J. J.; Dapprich, S.; Daniels, A. D.; Farkas, Ö.; Foresman, J. B.; Ortiz, J. V.; Cioslowski, J.; Fox, D. J. Gaussian 09 Revision A.1. Gaussian Inc. **2009**.
(22) Weigend, F.; Ahlrichs, R. Balanced Basis Sets of Split Valence, Triple Zeta Valence and Quadruple Zeta Valence Quality for H to Rn: Design and Assessment of Accuracy. *Phys. Chem. Chem. Phys.* **2005**, *7* (18), 3297–3305. https://doi.org/10.1039/B508541A.
(23) Häussermann, U.; Dolg, M.; Stoll, H.; Preuss, H.; Schwerdtfeger, P.; Pitzer, R. m. Accuracy of Energy-Adjusted Quasirelativistic Ab Initio Pseudopotentials. *Molecular Physics* **1993**, *78* (5), 1211–1224. https://doi.org/10.1080/00268979300100801.
(24) Küchle, W.; Dolg, M.; Stoll, H.; Preuss, H. Energy-adjusted Pseudopotentials for the Actinides. Parameter Sets and Test Calculations for Thorium and Thorium Monoxide. *The Journal of Chemical Physics* **1994**, *100* (10), 7535–7542. https://doi.org/10.1063/1.466847.
(25) Leininger, T.; Nicklass, A.; Stoll, H.; Dolg, M.; Schwerdtfeger, P. The Accuracy of the Pseudopotential Approximation. II. A Comparison of Various Core Sizes for Indium Pseudopotentials in Calculations for Spectroscopic Constants of InH, InF, and InCl. *The Journal of Chemical Physics* **1996**, *105* (3), 1052–1059. https://doi.org/10.1063/1.471950.
(26) Tomasi, J.; Persico, M. Molecular Interactions in Solution: An Overview of Methods Based on Continuous Distributions of the Solvent. *Chem. Rev.* **1994**, *94* (7), 2027–2094. https://doi.org/10.1021/cr00031a013.
(27) Barone, V.; Cossi, M. Quantum Calculation of Molecular Energies and Energy Gradients in Solution by a Conductor Solvent Model. *J. Phys. Chem. A* **1998**, *102* (11), 1995–2001. https://doi.org/10.1021/jp9716997.
